# A comparison of missing data approaches for linear regression with missing not at random outcome and predictors

**DOI:** 10.1177/09622802261458074

**Published:** 2026-07-15

**Authors:** Tetiana Gorbach, Tim P Morris, James R Carpenter

**Affiliations:** 1 Department of Statistics, Umeå School of Business, Economics and Statistics, 8075 Umeå University, Umeå, Sweden; 2 MRC Clinical Trials Unit at University College London, London, UK; 3 London School of Hygiene and Tropical Medicine, London, UK

**Keywords:** Heckman selection model, uncertainty intervals, multiple imputation, imputation using NARFCS, imputation stacking, random indicator imputation

## Abstract

While most methods for missing not at random (MNAR) data in regression models address MNAR outcomes assuming fully observed predictors, real-world observational health and longitudinal studies often violate this assumption. This paper compares several approaches for handling MNAR data in linear regression when missingness depends on both partially observed outcomes and predictors. Through extensive simulations, we evaluate complete-case analysis, multiple imputation assuming missing at random, maximum likelihood estimation via the Heckman selection model, uncertainty intervals, multiple imputation under the Heckman selection model, not-at-random fully conditional specification, imputation stacking, and random indicator imputation. None of the methods consistently produced unbiased estimates or nominal coverage across all scenarios. However, not-at-random fully conditional specification was straightforward to implement and yielded coverage close to the nominal level in most scenarios, provided that the sensitivity parameters were specified near their true values. Our results highlight the importance of sensitivity analyses exploring various full-data models and careful parameter specification when addressing MNAR in both outcomes and predictors. We illustrate such sensitivity analyses using Betula study data on the relationship between longitudinal memory change and grey matter volume in aging. The association remained significant across most considered MNAR scenarios, reinforcing existing evidence for this relationship.

## 1. Introduction

Missing data are inevitable in research and may arise from equipment failure, participant dropout, or unwillingness to provide certain data, among others.^1^ For example, consider an investigation of the relationship between brain structural and cognitive changes based on the Betula study of aging.^2,3^ Of the 264 healthy participants who completed baseline brain imaging and cognitive testing, approximately 40% lacked follow-up brain and/or cognitive measurements, primarily because they dropped out of the study or opted out of brain imaging,^4^ making it impossible to observe their changes between the two measurement occasions. If missing information on longitudinal change is more common among less healthy individuals, the observed data will disproportionately represent healthier participants. Consequently, naive analyses of the observed data without accounting for the nature of missing data may yield conclusions that do not reflect the true state of the full population.

In statistical analyses, the nature of missingness is described by the missing data mechanism, defined as the probability that data are missing given all observed and unobserved values. Rubin^5^ introduced a widely used taxonomy for these mechanisms. Under the missing at random (MAR) mechanism,^5^ the probability that data are missing is unrelated to the unobserved values conditional on the observed data. The data would be MAR in the empirical study of the brain-cognitive relationship if dropout was related to a participant’s available measurements but not to their unobserved ones. When data are MAR, and the parameters of the missing data mechanism are distinct from those of the data model (i.e. the mechanism is ignorable), likelihood-based inferences based on the observed data are equivalent to those based on the full data, observed and unobserved (see Chapter 6.2 in Little and Rubin^6^). In this sense, the exact distribution of the missingness can be “ignored” for inferences if data are MAR.

Another scenario in longitudinal health studies is that a participant’s current health status influences their decision to drop out or decline some measurements. Since general health might be associated with unobserved structural brain and cognitive measures, and thus with their changes, the missingness may depend on unobserved data even after conditioning on the observed data. In such cases, the data are called missing not at random (MNAR). Analyses that ignore the MNAR nature of data may lead to biased and potentially misleading inferences. The challenge is that it is impossible to determine whether data are MNAR based solely on the observed data; under MNAR, the missingness depends on the unobserved data, which cannot be compared with the observed data, given that it is unobserved.

Consider linear regression to study the relationships between phenomena such as cognitive and brain changes, where one phenomenon is treated as an outcome, and the other as one of the predictors in the regression model. As in the brain structure–cognition relationship, some data for the outcome and the predictor may be missing. The most widely-used approaches for the regression analyses with missing data include complete-case analysis, which discards data from participants with any missing in the variables used in the regression,^6^ and multiple imputation,^7^ which fills in missing values multiple times. Complete-case analysis yields unbiased estimators of regression coefficients when the missingness is unrelated to any data or depends on predictors but not the outcome (see Example 3.3 in Little and Rubin^6^ and the discussion in Carpenter and Smuk^8^). Thus, under MNAR, complete-case analysis can be unbiased if missingness is related only to incomplete predictors. Standard multiple imputation techniques, which impute missing values based on the distribution of missing data given the observed data, are unbiased under MAR, since the observed data contain all relevant information about the missingness. However, if data are MNAR, these techniques may yield biased estimates of regression coefficients, even when missingness does not depend on the outcome.^1,9,10^

Multiple imputation has been extended to MNAR data based on the Heckman selection (i.e. probit) model by Galimard et al.^11,12^ and imputation stacking by Beesley and Taylor.^13^ The Heckman-based imputation method of Galimard et al.^12^ has only been evaluated for MNAR outcomes, while the imputation stacking method of Beesley and Taylor^13^ was evaluated in the case of either MNAR outcome or multiple MNAR predictors with outcome fully observed. Pattern-mixture modeling-based multiple imputation based on not-at-random fully conditional specification (NARFCS) is designed to handle multiple MNAR variables.^14^ This is a form of delta-adjustment,^9^ where imputations under MAR are adjusted to account for MNAR data. Another recently developed method, called the random indicator method^15^ is yet to be compared with any methods apart from complete-case analyses or imputation stacking.^13^ Neither NARFCS nor the random-indicator method has been evaluated for cases where both the regression outcome and predictors are missing. Other approaches to linear regression analyses with MNAR data, such as the maximum likelihood estimation based on the Heckman selection model,^16^ are designed for MNAR outcome but anticipate that predictors will be fully observed.

This paper aims to address gaps in the evaluation of the performance of the aforementioned methods, designed to handle MNAR data in regression. We investigate data-generating mechanisms where missingness depends on the incomplete predictor, the incomplete outcome, or both. Motivated by our example, where missingness may be related to unobserved changes in both brain structure and cognition, we are particularly interested in the performance of these methods when the probability of missingness depends on both partially observed outcome and predictors in linear regression. Such scenarios may arise not only in longitudinal studies of aging, as in our example, but in clinical trials, economic studies, or any other studies, where missingness is related to an unobserved factor that is also associated with the unmeasured variables used in the analyses. Moreover, since it is impossible to test from the observed data the exact relationship between the missingness and the unobserved values, the investigation of the MNAR mechanism where missingness depends on both regression outcome and predictors is a natural step in the sensitivity analyses that assesses how robust inferences are to different assumptions about the nature of missing data.

The paper is structured as follows. Section 2 introduces notation and describes methods that might handle missing not-at-random data in linear regression. Section 3 describes simulation studies that investigate the performance of the considered methods. An example of analyses using the considered methods to study the relationship between brain and cognitive changes is presented in Section 4. We finish with the discussion of the results and recommendations for the researchers working with regression analyses of incomplete data in Section 5.

## 2. Methods for handling MNAR: benchmarks, selection- and pattern mixture-based

This section introduces notation, the considered analysis model, and methods that might handle missing not-at-random data in linear regression. Before delving into specific methods, we will discuss our benchmark methods of complete-case analysis and multiple imputation under the assumption of MAR data. We then describe methods for handling MNAR data in regression analyses based on selection modeling and continue with those based on pattern-mixture modeling. For each method, we provide a brief description, the assumptions about the missing data (and other assumptions) required for its validity, the availability of extensions for situations with missing predictors and outcomes, and the method’s advantages and disadvantages. We note that the missing data problem we consider is one that some of the methods were never designed to address. Nevertheless, we explore how these methods can still be applied in such scenarios and what results they provide.

We consider linear regression (1) as the analysis model in this paper (also referred to in the literature as the model of interest, outcome model, or substantive model)

(1) $$E(Y|X,Z)=X\beta_{x}+Z\beta_{z},$$

where Y denotes a partially observed continuous outcome, X represents partially observed (possibly multivariate) predictors, and Z denotes fully observed (possibly multivariate) predictors. We assume that the model is correctly specified and that the goal is to recover the inference one would obtain if no values were missing.

**Figure 1.**
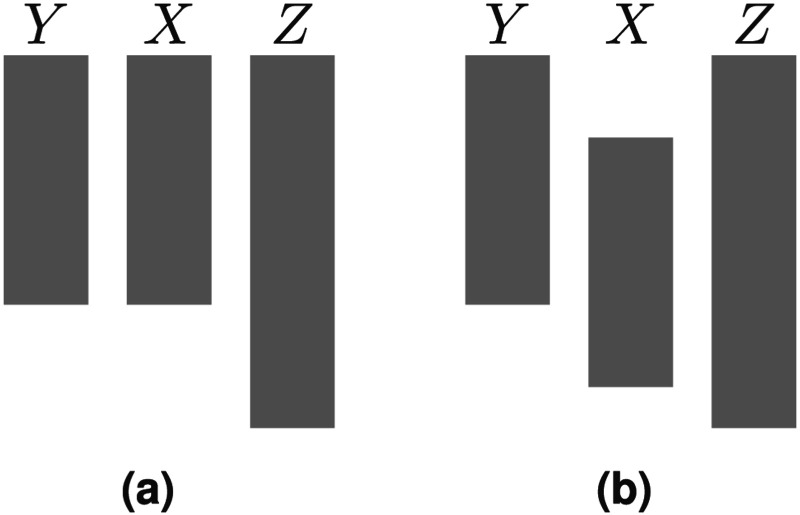
Schematic depiction of missing data patterns with partially observed univariate Y and X and fully observed Z. Imagine that case identifiers are on the vertical axis and dark shading indicates data observed. (a) Simultaneous monotone and (b) Non-monotone.

The choice of statistical methods for analyzing data with missing values depends, among other factors, on the missingness pattern, which indicates which values were observed and which were not.^1^ In this paper, we consider two patterns: the simultaneous monotone pattern, motivated by dropout in longitudinal studies, and the non-monotone pattern, which commonly arises in diverse observational datasets. A simultaneous monotone pattern (see Figure 1(a)), where data on some variables are missing for the same subset of participants, would appear if all missing data were due to dropout before follow-up in the memory-brain relationship example. Such a pattern might also appear in surveys, where respondents skip entire sections of a questionnaire. In contrast, the non-monotone pattern (also called general pattern;^1^ see Figure 1(b)), does not follow any such structure. The variables cannot be arranged in a monotone manner based on missingness. This is the most general missing data pattern and the one typically found in observational data across various fields.

After describing the patterns of missingness, it is essential to consider the mechanisms that generate these patterns. Typically, one distinguishes between the three missing data mechanisms: missing completely at random, MAR, and MNAR, as discussed in the introduction. Because data that are missing completely at random also satisfy the conditions for MAR, it will not be considered further. To define the missing data mechanisms of MAR and MNAR, some additional notation should be introduced. Consider J variables relevant to the analysis for each unit of a sample of size n, arranged into a vector-valued random variable V of dimension nJ. Similarly, let R be a vector-valued random variable representing the response indicator for V (also known as the observed data indicator). Each element of R is equal to one if the corresponding element of V is observed, and 0 if it is missing; that is, $R_{i}=1$ if $V_{i}$ is observed and $R_{i}=0$ if $V_{i}$ is missing, i=1,…,nJ. The definitions of MAR and MNAR data rely on the properties of the missing data mechanism, which is the conditional distribution of the response indicator given the data, P(R|V) .

The data are missing at random (MAR) if the probability of the actually observed response pattern r does not depend on the values of the unobserved data, given that the observed data are identical.^17^ Mathematically, this is expressed as:

(2) $$P(R=r|V=v)=P(R=r|V=v^{*}),$$

where r,v, and $v^{*}$ are any values such that the observed data are the same, that is $\left\{v_{i}:\;r_{i}=1\}=\{v_{i}^{*}:\;r_{i}=1\right\}$ .

On the other hand, data are MNAR if there exists a combination of values $r,v,v^{*}$ with the same observed data $\left\{v_{i}:\;r_{i}=1\}=\{v_{i}^{*}:\;r_{i}=1\right\}$ , but different conditional probability of the response indicator:

(3) $$P(R=r|V=v)\neq P(R=r|V=v^{*}).$$

The definition of MAR in (2) is also known as *everywhere MAR*.^17^ Rubin’s definition^5^ is more specific, focusing on a particular realization of r,v, and $v^{*}$ . Definition (2) is the one that is typically used in most missing data literature. In our setting, it follows from (2) that when the response indicator R is independent of variables X and Y given a fully observed predictor Z (expressed as R⊥⊥X,Y|Z ), X and Y are missing at random. Conversely, if R depends on X or/and Y given Z , then Y and X are called MNAR.

### 2.1. Complete-case analysis

The most common approach to deal with incomplete data sets is so-called *complete-case analysis*, also known as *listwise deletion* and *complete records analysis*. Complete-case analyses use only the cases for whom the values of all variables in the model are observed (Section 3.2 of Little and Rubin^1^). The complete-case estimator is unbiased when the probability of missing data does not depend on the regression outcome and the model is correctly specified.^10^ The method is readily applicable to multivariate missing data as the cases with missing data for *any* variable in the analysis model are discarded. Complete-case is the default in most software for analyses of incomplete data sets.

The primary advantage of this method is its simplicity. However, a notable drawback is the information loss, leading to reduced precision, and potential bias, especially when the probability of missing data depends on the regression outcome.

### 2.2. Multivariate multiple imputation assuming MAR (MI MAR)

Multiple imputation^7,9,18^ is a popular and well-developed approach for addressing missing data which easily handles multiple incomplete variables. It consists of three steps:

1. impute: Fill in each missing value K times from an estimate of the conditional predictive distribution of the missing given observed data based on the selected imputation model. This creates K ‘completed’ data sets that contain the observed and the imputed data.
2. analyse: Estimate the parameters of interest by fitting the analysis model to each of K completed data sets.
3. pool: For inference, combine the results of the K analyses using *Rubin’s rules*.^7,19^

Step 1 involves selecting an imputation model, which includes choosing the model type and selecting the variables to be used in the imputation. In practice, the default approach at step 1 in software implementations is to assume the data are MAR to estimate the conditional predictive distribution and then use this distribution to draw (i.e. impute) the missing values given the observed. However, many implementations permit the possibility of injecting extra information that reflects MNAR, as we will describe below.

A notable advantage of multiple imputation lies in its intuitive and straightforward implementation through available software, for example, using multivariate imputation using chained equations (MICE) algorithm^20^ that utilizes fully conditional specification. However, there are drawbacks to multiple imputations *via* the MICE algorithm. For example, van Buuren^9^ in Chapter 12.1 states, “The major danger of the technique is that it may provide nonsensical or even misleading results if applied without appropriate care or insight.” MICE has a less clear-cut theoretical basis.^21^ Furthermore, the imputation model might be incompatible with the analysis model, leading to biased estimators.^22^ Additionally, as highlighted by Wulff and Jeppesen,^23^ navigating around potential pitfalls of multiple imputation might be challenging.

### 2.3. Approaches for the analysis of MNAR data based on selection models

Analyses of data sets under the assumption of MNAR data require specification of the full-data distribution P(V,R) . The two most popular specifications are pattern-mixture models and selection models. Pattern-mixture models, introduced by Little,^24^ express the full-data distribution P(V,R) as a product P(V,R)=P(R)P(V|R) . In this formulation, the distribution of data V is conditioned on the observed data pattern R . Selection models characterize the joint distribution of full data using the expression P(V,R)=P(V)P(R|V), specifying a marginal model for data P(V), and the distribution of response indicator given the data P(R|V).

#### 2.3.1. Estimation based on the Heckman selection model

The well-known Heckman selection model^16^ assumes missingness in the regression outcome Y. It employs a probit regression for the response indicator:

(4) $$\begin{array}{r} \left\{ \begin{array}{l} Y=X\beta_{x}+Z\beta_{z}+\varepsilon ,\\ R^{*}=W\gamma +\varepsilon_{r},\\ \left(\begin{array}{c} \varepsilon_{r}\\ \varepsilon \end{array}\right)∼N\left(\left(\begin{array}{c} 0\\ 0 \end{array}\right),\left(\begin{array}{c} 1\;\rho \sigma \\ \rho \sigma \;\sigma^{2} \end{array}\right)\right), \end{array}\right. \end{array}$$

where Y is observed and R=1 if $R^{*}\geq 0;$ Y is missing and R=0 if $R^{*}<0.$ The predictor W may include variables from X and Z, as well as additional variables associated with $R^{*}$ but not with Y . The method is developed under the assumption that the predictors {X,Z}, and W have no missing values. When the correlation between the error of the analysis and response (also called selection) model is not zero, ρ≠0, the response indicator depends on possibly missing Y and the data are MNAR. The Heckman selection model can be fitted using full-information maximum likelihood (FIML) or a two-step procedure.^16^ For comparisons in this article, we use maximum likelihood estimation, as it is considered to be more precise than the two-step estimation.^16,25^

The maximum likelihood estimates from the Heckman selection model are consistent when the model is identified and the outcome and the response indicator models are correctly specified. The identifiability of the model relies on the bivariate normality of the errors and the existence of global maxima of the likelihood function.

The method is developed only for missingness in the outcome, Y. When data are missing in the outcome Y and predictors X , there are two possible adaptations to the Heckman selection model. The first is to exclude all incomplete predictors from the response model. However, this can lead to misspecification of the response model if the excluded predictors are actually relevant to the model. Another option is to exclude cases with missing predictors – similar to complete case analysis but exclusion based only on predictors. If both the outcome and predictors are missing for the same cases, only complete cases remain, rendering the Heckman selection model irrelevant.

The disadvantages of the method are discussed in Puhani,^25^ for example. First, when the sets {X,Z}, and W are the same, identification in the Heckman selection model relies on the non-linearity of the inverse Mills ratio λ=ϕ(−Wγ)/Φ(Wγ), where ϕ and Φ denote the probability density function and cumulative distribution function of a standard normal variable, respectively. However, the inverse Mills ratio is approximately linear over a wide range of its argument Wγ . To avoid multicollinearity that leads to identification issues, it is therefore recommended to include variables {W} in the model for the response indicator that are not among the predictors {X,Z} of the outcome model (strategy known as exclusion restriction). Additionally, maximum likelihood estimation might perform poorly when the errors are not bivariate normal. Pigini^26^ discusses the non-normality of errors due to incorrect specification of outcome and response indicator models, for example, overlooking non-linear or interaction terms. The assumption of bivariate normality of the errors has been relaxed in multiple ways.^25,27,28^ Toomet and Henningsen^29^ extended the Heckman model to analyze binary outcomes and general linear models.

The Heckman selection model, which uses a probit link function, is only one of the selection models used in regression analyses. One alternative is a logistic selection model that directly includes the outcome variable from the analysis model as one of the predictors of the response indicator, rather than linking the analysis and response models through the correlation of the error terms.

#### 2.3.2. Uncertainty intervals based on the Heckman selection model (UI)

Instead of estimating the correlation of errors ρ in the Heckman selection model (4), which heavily relies on the model’s identifiability, Genbäck et al.^30^ proposed to use subject-matter knowledge to specify a plausible range for ρ . For each value of ρ, the analysis model parameters can be estimated by bias-adjusting the ordinary least squares estimates based on the complete cases by a factor that depends on ρ and the response indicator model. The union of confidence intervals for regression coefficients constructed for each specific value of ρ forms an uncertainty interval. The lower bound of the uncertainty interval of Genbäck et al.^30^ is the minimum of the lower bounds of confidence intervals and the upper bound is the maximum of the upper bounds. An uncertainty interval covers the true value of the parameter with at least a pre-specified probability if the true value of ρ is within the considered range of ρ (is “confidence valid” as described by Rubin^31^), given that the considered analysis and response models are correctly specified.

The uncertainty intervals estimation method of Genbäck et al.,^30^ like the Heckman selection model, is developed for missing data only in the analysis model outcome, Y. In case of multivariate missingness, the options are to either exclude incomplete predictors from the response and analysis models or to exclude subjects with incomplete predictors. The impact of these exclusions on the properties of uncertainty intervals remains to be investigated.

Uncertainty intervals of Genbäck et al.^30^ do not rely on the exclusion-restriction assumption, unlike the estimation of the Heckman selection model. Therefore, the method is expected to perform well even if predictors in the outcome and missing models are the same. Uncertainty intervals, however, rely on the specified range of ρ and the correct specification of the analysis and response models. If the true value of ρ lies outside the considered range, the uncertainty intervals may not provide reliable coverage and are not confidence valid.^31^ Furthermore, the impact of model misspecification on the uncertainty intervals has not been investigated yet. Additionally, like the Heckman selection model, uncertainty intervals rely on the assumption of bivariate normality of the errors; some relaxation of this was discussed in Genbäck et al.^30^

#### 2.3.3. Multiple imputation under the Heckman selection model (MI HE)

Galimard et al.^11,12^ considered multiple imputation of the outcome under the Heckman selection model (4) using the fact that the expectation of outcome Y conditional on the outcome being missing is

$$E(Y|X,Z,W,R=0)=X\beta_{x}+Z\beta_{z}+\frac{-\phi (W\gamma )}{1-\Phi (W\gamma )}\beta_{\lambda },$$

Galimard et al.^11,12^ provide algorithms to properly impute missing continuous outcome from to the imputation model

$$Y=X\beta_{x}+Z\beta_{z}+\frac{-\phi (W\gamma )}{1-\Phi (W\gamma )}\beta_{\lambda }+\eta ,\;\eta ∼N(0,\sigma_{\eta }^{2}).$$

The imputation in Galimard et al.^11^ is based on Heckman’s two-step model estimates of $\beta_{x},\beta_{z},\beta_{\lambda },\sigma_{\eta }^{2},\gamma ,$ while Galimard et al.^12^ uses Heckman’s one-step maximum likelihood estimates and also considers binary outcomes.

Multiple imputation under the Heckman selection model was developed for MNAR analysis model outcome and can be used in case of multivariate missing *via* multiple imputation using mice R package. While the tool is available, the properties of the method when data are MNAR in *multiple* variables and are imputed sequentially using the Heckman model have yet to be investigated.

#### 2.3.4. Multiple imputation using imputation stacking (MI stacking)

This method begins by producing multiple imputations under MAR. The imputed data sets are then ‘‘stacked’’ on each other.^13,32^ In this stacked data set, each row represents a vector of imputed and observed values for one imputation for one subject. Each row in the stacked data set is assigned a weight based on the concept of importance sampling^33^ aiming to ensure that the weighted distribution of imputations represents the desired distribution of the missing data. The analysis model is fitted to this weighted stacked data set.

When only one variable is MNAR or several variables are missing for the same people, the weights are proportional to the odds of data being missing, P(R=0|V)/P(R=1|V).

When data are MNAR in two variables, for example, the data should be imputed from different distributions for different missing data patterns. Consider model (1) with partially observed outcome Y and partially observed predictor X . According to Appendix A.3 in Beesley and Taylor,^13^ for a case with missing predictor X and observed outcome Y , predictor X should be imputed from $f(X|Y,Z,R_{x}=1)$ and weighted by $P(R_{y}=1|X,Y,Z,R_{x}=0)(P(R_{x}=0|X,Y,Z))/(P(R_{x}=1|X,Y,Z)).$ Here, $R_{x}$ is the response indicator for X and is equal to 1 if X is observed and 0 otherwise, $R_{y}$ is the response indicator for Y. Similarly, for a case with missing Y and observed X, the missing Y should be imputed from $f(Y|X,Z,R_{y}=1)$ and weighted by $P(R_{x}=1|X,Y,Z,R_{y}=0)(P(R_{y}=0|X,Y,Z))/(P(R_{y}=1|X,Y,Z)).$ For a case with missing X and Y, they should be imputed from $f(X,Y|Z,R_{x}=1,R_{y}=1)$ and weighted by $(P(R_{x}=0,R_{y}=0|Y,X,Z))/(P(R_{x}=1,R_{y}=1|Y,X,Z)).$ Parametric assumptions in selection models can make all parameters identifiable from observed data (see Section 8.3.2 in Daniels and Hogan^34^), but their estimation, for example through numerical optimization of maximum likelihood,^32^ requires substantial effort. The stacking method offers an alternative: using analyst-chosen parameter values for the incomplete variables in these models, users manually define functions that compute weights based on imputed values.

The method’s implementation is straightforward when data are MNAR only in the outcome. When data are MNAR in several variables, imputation from different distributions for different missing data patterns requires care. The method’s implementation complexity stems from weight specification: the number of parameters to specify and functions to calculate the weights grows very fast with the number of MNAR variables. For example, with p MNAR variables in the non-monotone missing data pattern, one has to assign $2^{p}$ functions to calculate the weights (one function for each missing data pattern), and each function may depend on multiple parameters that should be specified by a user.

### 2.4. Approaches for the analysis of MNAR data based on pattern-mixture models

#### 2.4.1. Multiple imputation using not-at-random fully conditional specification

Multiple imputation using not-at-random fully conditional specification (MI NARFCS) was introduced by Leacy^35^ and discussed in Tompsett et al.^14^ It is a form of multivariate δ -adjustment method^9,36^ and, in essence, involves adjustment of the imputation models for some assumed discrepancy in the conditional predictive distributions of the missing and observed data. As in multiple imputation of MAR data using fully conditional specification,^37^ the missing values are imputed one variable at a time from the conditional distribution of missing data given the observed data; the difference is that response indicators for all incomplete variables are included in each conditional imputation model.

For example, in the case of missing outcome Y and predictor X in the regression, they are imputed from $f(Y|X,Z,R_{x},R_{y}=0,\delta_{y})$ and $f(X|Y,Z,R_{x}=0,R_{y},\delta_{x}),$ respectively. These imputation distributions in MI NARFCS are parameterized so that the observed-data likelihood is a constant function of the parameters $\delta_{y}$ associated with terms involving $R_{y}$ in $f(Y|X,Z,R_{x},R_{y},\delta_{y})$ and the parameters $\delta_{x}$ associated with $R_{x}$ in $f(X|Y,Z,R_{x},R_{y},\delta_{x}),$ while varying with other parameters. The parameters $\delta_{y}$ and $\delta_{x}$ are then, by definition,^34^ sensitivity parameters, which are not identifiable from the observed data. Analysts must specify plausible values for these parameters to conduct meaningful sensitivity analyses. However, because these sensitivity parameters reflect conditional differences: differences between observed and missing data within strata defined by other variables, they can be difficult to elicit from experts. To address this, Tompsett et al.^14^ describe calibration to derive plausible conditional sensitivity parameters from marginal sensitivity parameters, that is, the sensitivity parameters in distributions marginalized over at least some variables, which are easier for domain experts to provide. Finally, note that setting all sensitivity parameters to zero does not yield the standard MAR fully conditional specification, since response indicators for other variables remain included in the imputation models.

The advantage of NARFCS is its straightforward implementation, for example using the mice R package. However, NARFCS requires the specification of conditional models, as in the standard MICE algorithm, and its major challenge is specifying plausible values of the sensitivity parameters in these conditional models. Nevertheless, if the objective is unbiased estimation of the analysis model parameters, the accuracy of NARFCS may be compromised when marginal sensitivity parameters are used as proxies for conditional ones or when the specified values deviate substantially from their true values implied by the model.

#### 2.4.2. Random indicator imputation (MI RI)

Most of the methods mentioned above require analysts to specify the values of parameters describing the difference between missing and observed data. Because the missing data are unobserved, this process relies heavily on subject-matter knowledge and can be challenging. Also, the conditional nature of the parameters can prove challenging for determining the values of the parameters, even for subject-matter experts, as highlighted in Tompsett et al.^14^ The random indicator (RI) method^15^ avoids specifying the differences between the observed and missing data and instead estimates these differences from the observed data under additional assumptions.

Specifically, the random indicator method assumes that the missing part of an incomplete variable X has the same variance as the observed part but a shifted mean. The probability of X being observed is assumed to follow a logistic function. The method involves drawing another response indicator from this logistic function. The means of X given the initial response indicator and the newly drawn one are shifted in relation to the conditional mean of the observed data (see Proposition 2 in Jolani et al.).^15^ The missing values of X are imputed using the fact that these shifts can be estimated from the observed data identified due to the assumptions of the RI method.

This method is developed for missingness in one variable, but it can be extended to handle multivariate missingness *via* joint modeling or fully conditional specification.

The advantage of the RI method is that it does not require specification of any parameters, unlike other methods, and it is easy to implement. However, as noted by Jolani et al.,^15^ the estimates based on the RI method might have a slightly larger variance than the complete cases method. Additionally, the method assumes a logistic distribution for the response indicator, and models the difference between observed and missing values of a variable solely as a shift in the mean. The RI method’s sensitivity to these assumptions and the method’s properties when data for multiple variables are MNAR have not yet been thoroughly studied.

### 2.5. Methods not used in the comparisons

The expectation–maximization algorithm, Chapter 8 in Little & Rubin,^1^ applied to the missing data problem necessitates distinct manual implementation in software for each distribution. While the method is suitable for specific studies, we chose to exclude it from the comparisons.

Inverse probability weighting methods^38^ use weighted complete case analyses of the analysis model. The weights for each complete case are the inverse of the fitted probability of being complete. When predictors in these selection model(s) are unobserved, as in our studies, the estimators of the weights are expected to be biased.

Reference-based imputation approaches, as described by Carpenter et al.,^18^ assumes that the distribution of missing measures mirrors that of observations within a specified reference group. Initially designed for longitudinal studies, the method operates under the assumption that participants continue to behave similarly to a reference group even after dropout. However, in our comparisons, we examine studies where a clear reference group is absent (as we do not consider trial studies), so reference-based approaches are not applicable.

Ogundimu and Collins^39^ provided an imputation algorithm similar to the Heckman model but with a bivariate Student’s *t*-distribution of errors, contrasting with the imputations based on the normality of the errors in Galimard et al.^12^ Additional file 2 in Galimard et al.^12^ compares these two imputation methods in the case of MNAR outcome and MAR predictor, concluding that results are similar.

Note that shared-parameter models, another approach for MNAR data modeling in addition to the selection and pattern-mixture models, are not considered in this paper.

## 3. Simulation studies

Table 1 gives an overview of the evaluations of the considered methods in the papers that introduced the methods. We conduct two simulation studies in Sections 3.1 and 3.2 that aim to compare the performance of MNAR methods in terms of coverage, bias, and precision of linear regression parameter estimators when some data are missing in the outcome and predictors, and the probability of data being missing depends on missing outcome’s and/or predictor ’s values. Simulation study 1, motivated by the empirical study, considers simultaneous missing data in both the outcome and the predictor, as depicted in Figure 1(a). Simulation study 2 considers the non-monotone missing data pattern illustrated in Figure 1(b). The detailed software implementations of the considered methods in the simulation studies are provided in the supplementary material.

**Table 1.** Summary of simulation studies that evaluate the performance of the considered methods.

Feature/Method of primary focus	UI	MI HE	MI stacking	MI NARFCS	MI RI	This paper
Reference:	Genbäck et al.^30^	Galimard et al.^12^	Beesley and Taylor^13^ Appendix D	Tompsett et al.^14^	Jolani and van Buuren^15^	
Repetitions:	10,000	1,000	1,000	1,000	100	1,000
Observations:	100, 350, 753	500	2,000	1,000	200, 1,000	1,000
Imputations:	not applicable	50	50	10	5	20
MI iterations:	not applicable	20	not specified	not specified	10	10
Analysis model:	Y∼X linear	Y=f(X,Z) probit, linear	$Y=f(X_{1},X_{2},Z)$ linear	$(Y_{1},Y_{2})∼1$	$Y=f(Z_{1},Z_{2})$ linear	$Y=f(X,Z_{1})$ linear
Missing in:	Y	Y, MAR in X	$X_{1}$ , MCAR in $X_{2}$	$Y_{1},Y_{2}$	Y	Y,X
% missing	not specified	∼30%	$∼50\%\;X_{1}$ and $∼25\%\;X_{2}$	$∼30\%\;Y_{1}$ and $∼25\%\;Y_{2}$	not specified	not specified
Missingness modeling:	$P(R_{Y}=1)=f(X)$	$P(R_{Y}=1)=f(Y,X,Z,W)$ $P(R_{X}=1\;)=f(Z,W)$ or $P(R_{X}=1)=f(Z,Y)$	$P(R_{X_{1}}=1)=f(X_{1},Y)$ $P(R_{X_{2}}=1)=0.5$	$Y_{1}∼Y_{2}+R_{Y_{1}}+R_{Y_{2}}$ $Y_{2}∼Y_{1}+R_{Y_{1}}+R_{Y_{2}}$	$P(R_{Y}=1)=f(Y,Z_{1})$	Described in Section 3
Methods evaluated in simulations:	Complete cases UI HE 2step	All cases Complete cases ML HE (using complete cases in Z ) MI HE ml ( Z imputed via “norm”) HE 2step (using complete cases in Z ) MI HE 2step ( Z imputed via “norm”)	Complete cases MI Stacking MI NARFCS MI RI	MI NARFCS	Complete cases MI MAR MI RI	All cases Complete cases MI MAR ML HE MI HE ml UI MI Stacking MI NARFCS MI RI
Performance measures:	CI cov. reg. coef Width of UIs	CI cov. reg coef Emp. SE % relative bias RMSE RMS estimated SE	Bias CI cov. reg coef. Average estimated variance	Bias Emp. SE CI cov. $EY_{1}$ , $EY_{2}$	CI cov. reg coef Regression coef.	Bias CI cov. reg coef Emp. SE MSE

UI: uncertainty intervals; MI HE: Multiple imputation under the Heckman selection model; MI Stacking: Multiple imputation using imputation stacking; MI NARFCS: Multiple imputation using not-at-random fully conditional specification; MI RI: Random Indicator imputation; ML HE: Maximum likelihood estimation based on the Heckman selection model; HE 2step: 2-step estimation based on the Heckman selection model; MI HE ml: Multiple imputation using Heckman’s one-step ML; CI cov. reg. coef: empirical coverage of the intervals for regression coefficients; Emp.SE: empirical standard error; RMSE: root mean squared error; RMS estimated SE: root mean square of the estimated standard error..

### 3.1. Simulation study 1: simultaneous monotone missing

Some of the considered analysis methods are based on the Heckman or logistic models, others on the pattern-mixture models. To ensure that our data-generating mechanisms did not favor one of these groups, the data was simulated using both the Heckman and logistic selection models and a pattern-mixture model.

**Figure 2.**
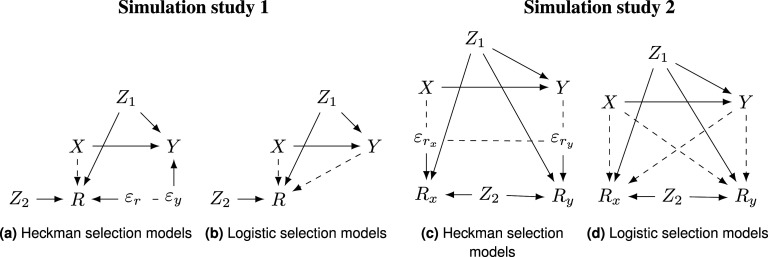
Data-generating mechanisms for selection models in the simulation studies. Edges indicate an inclusion of a variable in the outcome or response model as a main effect. Undirected edges represent correlations. Dashed edges denote relationships present in some scenarios.

For the Heckman and logistic selection models, the outcome data with a sample size of 1,000 was generated using the following parametric analysis model:

(5) $$Y=\beta_{0}+\beta_{x}X+\beta_{z}Z_{1}+\varepsilon_{y}.$$

We followed the simulation study in Beesley and Taylor^13^ and used $\beta_{0}=0,$ $\beta_{x}=\beta_{z}=0.5.$ Under the Heckman selection model, the response indicator was generated as follows (see Figure 2(a)):

$$R=I(\gamma_{x}X-Z_{1}+Z_{2}+\varepsilon_{r}>0),$$

where I denotes the indicator function, and $\left(X,Z_{1},Z_{2},\varepsilon_{y},\varepsilon_{r}\right)$ follows a multivariate normal distribution with mean zero and variances equal to 1, a covariance matrix where all variables are independent except for $\varepsilon_{y},$ $\varepsilon_{r}$ with correlation ρ. The predictor X and the outcome Y were observed if R=1, and missing otherwise, while covariates $Z_{1}$ and $Z_{2}$ were fully observed. When $\rho =\gamma_{x}=0,$ the data are MAR, otherwise data are MNAR. The variable $Z_{2}$ was introduced in the Heckman selection model to satisfy the exclusion criterion and facilitate convergence of the estimators that use the inverse Mills ratio. In the response indicator model, we fixed the regression coefficients for the “exclusion” predictor of less interest $Z_{1}$ to −1 and $Z_{2}$ to 1 to have different signs for these parameters. The intercept is set to 0 to get an average of 50% of outcome and predictor values missing.

For the logistic selection model, the outcome was generated using (5), while the response indicator was generated following a simulation study of Beesley and Taylor,^13^ Supplementary Section E (see Figure 2(b) for an illustration):

$$\begin{array}{rl} & \text{logit}(P(R=1))=\gamma_{x}X+\gamma_{y}Y-Z_{1}+Z_{2},\\ & (X,Z_{1},Z_{2},\varepsilon_{y}{)}^{⊤}∼N(0,I), \end{array}$$

where I denotes an identity matrix. We consider combinations of $\gamma_{x}$ and $\gamma_{y}$ taking values from {0, 0.5, − 0.5}. When $\gamma_{x}=\gamma_{y}=0,$ the data are MAR, otherwise the data are MNAR. Similar to the Heckman selection model, the intercept in the response indicator model is set to 0 to get on average 50% of outcome and predictor values missing.

We also considered a response model with the interaction between X and Y to investigate how the methods perform when the missing data mechanism is misspecified:

$$\begin{array}{r} R=I(XY+\varepsilon_{r}>0), \end{array}$$

where $\varepsilon_{r}∼N(0,0.05^{2})$ independent of X, $Z_{1}$ , $Z_{2},$ and $\varepsilon_{y}.$

For the pattern-mixture model, the outcome was generated using (5) and we consider

$$\begin{array}{r} \left\{ \begin{array}{l} R=Bern(0.5)\\ \left(X,Z_{1},Z_{2},\varepsilon_{y}{)}^{⊤}∼N(0,I\right)\\ Y=\left\{ \begin{array}{l} 1.5X+1.5Z_{1}+\varepsilon_{y},\text{if }R=1\\ -0.5X-0.5Z_{1}+\varepsilon_{y},\text{if }R=0. \end{array}\right. \end{array}\right. \end{array}$$

Here, X does not depend on the response indicator R, since otherwise, the mean $E(Y|X,Z_{1})$ marginalized over R is not a linear function of X, $E(Y|X,Z_{1})=(1.5X+1.5Z_{1})P(R=1|X,Z_{1})+(-0.5X-0.5Z_{1})P(R=0|X,Z_{1}).$

The estimands, that is, the targets of analyses, are the regression coefficients of the outcome analysis model, $\beta_{0}=0,\beta_{x}=0.5,\beta_{z}=0.5$ .

We consider the following methods of analysis (with some comments on implementations, please see the supplementary material for more details on the implementation): all-case analysis, which assumes all data are observed; complete-case analysis; and multiple imputation under the MAR assumption, referred to in the results as “MI MAR.” For MI MAR, missing predictor and outcome are imputed using a fully conditional specification, where the conditional models are fitted using Bayesian linear regression.

We then consider maximum likelihood estimation based on the Heckman selection model, where the incomplete predictor X is excluded from the selection model, referred to below as “ML HE X excluded.” The exclusion of X is necessary because the implementation does not support missing predictors, and restricting to observed X yields only complete cases in the considered pattern, making Heckman estimation irrelevant. Multiple imputation under the Heckman model,^12^ using a fully conditional specification, where the conditional model for the outcome follows the Heckman model structure, and the conditional models for the predictor are fitted via Bayesian linear regression, this approach is referred to as “MI HE.”

Uncertainty intervals’ software implementation *via* UI R-package^40^ also does not support missing predictors. We exclude X from the response model in the estimation and refer to this method as “UI X excluded.” We also assume ρ=0.3 which corresponds to the true value for some of data-generating mechanisms. However, since X is excluded, the selection model is misspecified.

Multiple imputation using imputation stacking, where in “MI Stacking SP” we define weights proportional to $\exp\;(-(0.5X_{im}+0.5Y_{im})),$ where $X_{im}$ and $Y_{im}$ denote (imputed or observed) values of $\left\{X_{i},Y_{i}\right\}$ in the m th completed data set, following equation (8) in Beesley and Taylor,^13^ assuming a logistic model for the response indicator. We set the values of the parameters for X and Y to 0.5 to represent the dependency of the weights on incomplete variables. The value 0.5 is an arbitrary (though reasonable) choice. Making such a choice is unavoidable because the observed data does not inform the strength of the dependence between the response indicator and incomplete variables; this assumed missing data mechanism corresponds to the one under $\gamma_{x}=\gamma_{y}=0.5.$ In the “MI stacking full data” method, we use full data to estimate the weights proportional to $\exp\;(-({\hat{\gamma }}_{x}X_{im}+{\hat{\gamma }}_{y}Y_{im})),$ using ${\hat{\gamma }}_{x}$ and ${\hat{\gamma }}_{y}$ from the logistic model $\text{logit}(P(R=1))=\gamma_{x}X+\gamma_{y}Y+\gamma_{z_{1}}Z_{1}+\gamma_{z_{2}}Z_{2}$ . The standard errors are estimated using the Jackknife method described by Beesley and Taylor.^13^

We implement three NARFCS methods. In “MI NARFCS Shift SP” we assume in the imputation models that those with missing Y or X have twice as large an intercept as those with the observed Y or X: $E(X|Y,Z_{1},Z_{2},R)=\mu_{0}+\mu_{1}Y+\mu_{2}Z_{1}+\mu_{3}Z_{2}+\mu_{0}(1-R)$ and $E(Y|X,Z_{1},Z_{2},R)=\nu_{0}+\nu_{1}X+\nu_{2}Z_{1}+\nu_{3}Z_{2}+\nu_{0}(1-R).$ To define input values of the shifts $\mu_{0}$ and $\nu_{0},$ we fit linear regressions of X on $Y,Z_{1},Z_{2}$ and of Y on $X,Z_{1},Z_{2}$ using the observed data to get the estimates ${\hat{\mu }}_{0}$ and ${\hat{\nu }}_{0}$ .

In “MI NARFCS Shift full data,” we also assume that the conditional distributions of X and Y for missing data differ from the conditional distribution for the observed data only by a shift in the intercept: $\mu_{4}$ in $E(X|Y,Z_{1},Z_{2},R)=\mu_{0}+\mu_{1}Y+\mu_{2}Z_{1}+\mu_{3}Z_{2}+\mu_{4}(1-R)$ and $\nu_{4}$ in $E(Y|X,Z_{1},Z_{2},R)=\nu_{0}+\nu_{1}X+\nu_{2}Z_{1}+\nu_{3}Z_{2}+\nu_{4}(1-R).$ However, now we use full data to estimate the shifts $\mu_{4}$ and $\nu_{4},$ which is unrealistic in practice but can be seen as a scenario when the specified value of the sensitivity parameter is close to the true value of the sensitivity parameter.

In “MI NARCFS Interactions full data” we allow the coefficients conditional distributions of X on $Y,Z_{1},Z_{2}$ and Y on $X,Z_{1},Z_{2}$ to vary across missingness patterns by including the interaction terms between the variables and the response indicator. $E(X|Y,Z_{1},Z_{2},R)=\mu_{0}+\mu_{1}Y+\mu_{2}Z_{1}+\mu_{3}Z_{2}+(\mu_{4}+\mu_{5}Y+\mu_{6}Z_{1}+\mu_{7}Z_{2})(1-R)$ and $E(Y|X,Z_{1},Z_{2},R)=\nu_{0}+\nu_{1}X+\nu_{2}Z_{1}+\nu_{3}Z_{2}+(\nu_{4}+\nu_{5}X+\nu_{6}Z_{1}+\nu_{7}Z_{2})(1-R).$ Here, we again unrealistically use full data to estimate the values of the coefficients for the interaction terms. Note that this is slightly different from using the true values.

Random indicator imputation is referred to below as “MI RI.”

For all methods, we fit the linear regression of Y on X and $Z_{1}$ as the analysis model. All analyses within simulation studies were performed using R Statistical Software, version 4.2.1.^41^

We generate 1,000 repetitions of data with sample size 1,000 for each data-generating mechanism, use 20 imputations, and 10 iterations within each imputation. We use the following performance measures: bias, mean squared error, empirical SE, model SE, and empirical coverage of 95% confidence intervals.

**Figure 3.**
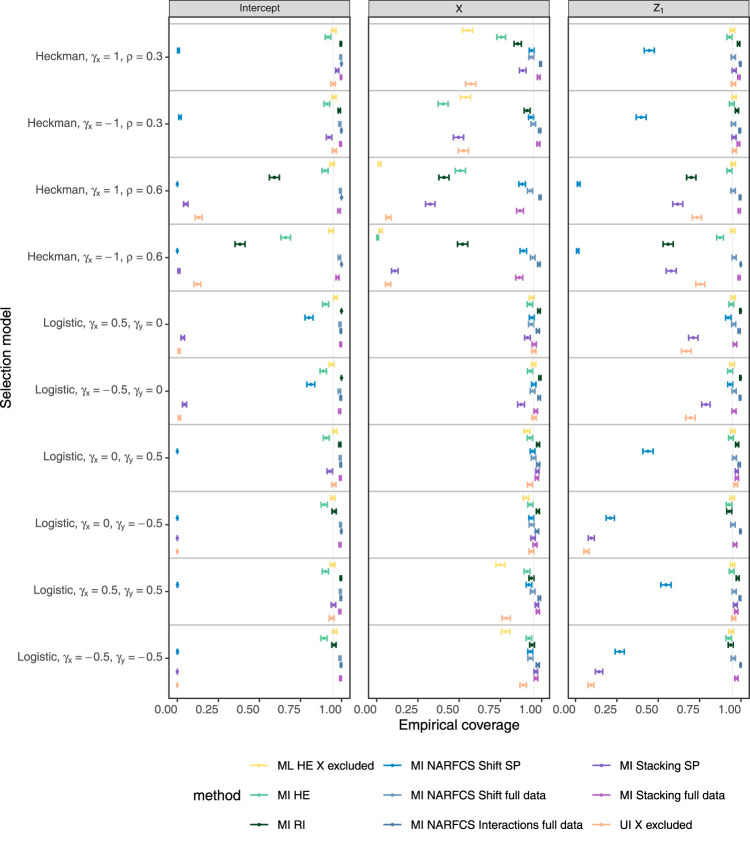
Empirical coverage of 95% confidence intervals in simulation study I, where X and Y are missing for the same participants. The whiskers represent the Monte Carlo standard error of the coverage estimate. The vertical-axis labels represent different selection models, and within each panel are the results of the various analysis methods.

Results for simulation study 1 are presented in Figure 3, Supplementary Tables S3–S6 and discussed in Section 3.3.

### 3.2. Simulation study 2: non-monotone missing

Simulation study 2 has the same aim, estimands, and performance measures as simulation study 1. However, two separate response indicators, one for X and another for Y, are necessary to describe the missingness. The covariate and outcome data was generated using equation (5) for a sample size of 1,000. The response indicators under the Heckman selection model were generated as follows:

$$\begin{array}{r} \left\{ \begin{array}{l} R_{x}=I(-Z_{1}+Z_{2}+\varepsilon_{r_{x}}>0)\\ R_{y}=I(-Z_{1}+Z_{2}+\varepsilon_{r_{y}}>0). \end{array}\right. \end{array}$$

We generate $\left(X,Z_{1},Z_{2},\varepsilon_{y},\varepsilon_{r_{x}},\varepsilon_{r_{y}}\right)$ from a multivariate normal distribution with mean zero and unit variance for each component. The variables are mutually independent except for specific correlations that define the missing data mechanism. The correlation $\text{Corr}(\varepsilon_{r_{x}},X)$ is set to 0.3 if the response indicator for X, denoted $R_{x},$ depends on X and 0 otherwise. The correlations $\text{Corr}(\varepsilon_{r_{x}},Y),$ $\text{Corr}(\varepsilon_{r_{y}},X),$ $\text{Corr}(\varepsilon_{r_{y}},Y)$ are each set to either 0 or 0.3 depending on whether the corresponding response indicator depends on the respective variable. When all four correlations are zero, the missingness mechanism is missing at random. For all considered scenarios, we fix $\text{Corr}(\varepsilon_{r_{x}},\varepsilon_{r_{y}})=0.3.$ This setup yields a total of 16 distinct data-generating mechanisms. As in simulation study 1, we fix the response model coefficients for predictors of less interest, $Z_{1}$ and $Z_{2},$ as well as fix the intercept to 0. This setup yields an average of 50% of outcome and predictor values missing.

Under the logistic selection model, we consider a logistic model for the response indicator following a simulation study of Beesley and Taylor,^13^ Supplementary Section E:

$$\begin{array}{r} \left\{ \begin{array}{l} \text{logit}(P(R_{x}=1))=\gamma_{xr_{x}}X+\gamma_{yr_{x}}Y-Z_{1}+Z_{2}\\ \text{logit}(P(R_{y}=1))=\gamma_{x_{r_{y}}}X+\gamma_{y_{r_{y}}}Y-Z_{1}+Z_{2}\\ (X,Z_{1},Z_{2},\varepsilon_{y}{)}^{⊤}∼N(0,I). \end{array}\right. \end{array}$$

The coefficients $\gamma_{xr_{x}},\gamma_{yr_{x}},\gamma_{xr_{y}},\gamma_{yr_{y}}$ determine whether the response indicators depend on X and Y . Each of the four coefficients is set to 0.5 when the corresponding response indicator depends on the associated variable. We exclude the MAR mechanism, which corresponds to all coefficients equal to zero, since MAR was considered under the Heckman selection model. This yields 15 distinct data-generating mechanisms.

We also consider two pattern-mixture models: one where the response indicator for X is independent of X , and another where it depends on X (see Supplementary Material for details).

In simulation study 2, we use the same methods as in simulation study 1. However, due to differences in data structure, the implementation of some methods differs from that in simulation study 1 as follows. For the Heckman selection model estimated by maximum likelihood, we additionally provide estimation from the subsample with observed X in “ML HE X cc,” which was not feasible in simulation study 1 since Y is observed for every case in such a subsample. For the multiple imputation under the Heckman selection model, in addition to “MI HE,” we consider “MI HE X cc” that subsets the sample to cases with X observed.

We provide the uncertainty intervals subsetting the sample to cases with X observed (“UI X cc”). We set the value of the correlation ρ to 0.3, the true value of the correlation for some of the processes. Additionally, we provide results for “UI X cc” assuming ρ∈[−0.99,0.99] in Supplementary Tables S18 and S19.

In “MI Stacking full data,” we use imputations provided by the “MI MAR” for those cases with either X or Y missing. To impute from $f(X,Y|Z_{1},Z_{2},R_{x}=1,R_{y}=1)$ for the cases with X and Y missing, we use a subset of the observed data that contains only cases with X and Y missing simultaneously or both observed. We assume logistic regressions for the response data indicators and define the weights, following Section 2.3.4, using predictions from the corresponding logistic regressions fitted to the full data.

Specifying all the models and parameters when imputing from different distributions for different missing data patterns can be challenging. Therefore, in “MI Stacking X MAR,” we assume that only the regression outcome is MNAR, while incomplete predictor is imputed using a fully conditional specification where conditional models for the predictor are fitted using Bayesian linear regression. Assuming a logistic regression model for the response indicator for the outcome, we estimate the coefficient corresponding to the outcome within this logistic model using the full data to define weights.

“MI NARFCS Shift SP” method is the same as in simulation study 1. In “MI NARFCS Shift full data” and “MI NARCFS Interactions full data” we impute incomplete variables using missing indicators for other incomplete variables according to the NARFCS algorithm.^14^

**Figure 4.**
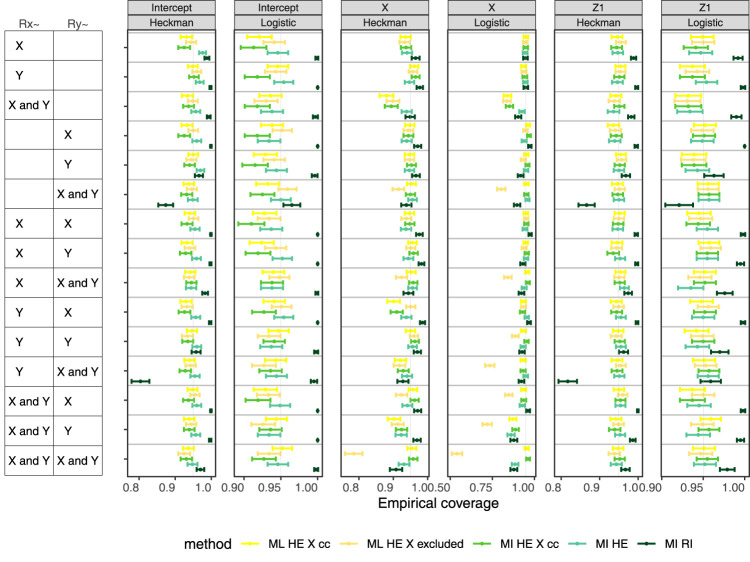
Empirical coverage of 95% confidence intervals in simulation study 2 with non-monotone missing in X and Y for the methods that make distributional assumptions and do not require parameter specification. The whiskers represent the Monte Carlo standard error of the coverage estimate. The vertical-axis represents different selection models. The panel to the left describes the relationship between the response indicators and the incomplete variables for each data-generating mechanism. In this panel, Rx ∼ indicates that the response indicator for X is related to the variable(s) listed in that row (either X, Y , or both). Similarly, Ry ∼ indicates the relationship for the response indicator of Y .

**Figure 5.**
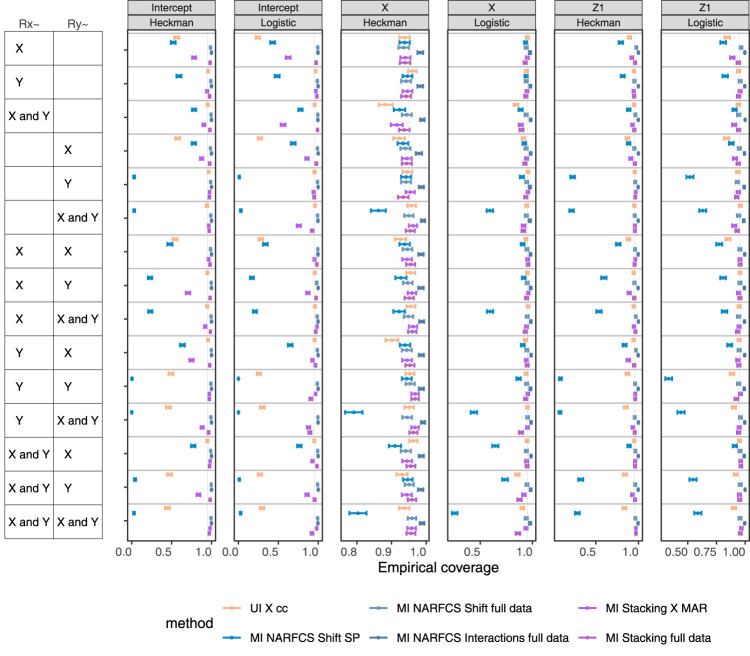
Empirical coverage of 95% confidence intervals in simulation study 2 with non-monotone missing in X and Y for the methods that require parameter specification. The whiskers represent the Monte Carlo standard error of the coverage estimate. The vertical-axis represents different selection models. The panel to the left describes the relationship between the response indicators and the incomplete variables for each data-generating mechanism. In this panel, Rx ∼ indicates that the response indicator for X is related to the variable(s) listed in that row (either X, Y , or both). Similarly, Ry ∼ indicates the relationship for the response indicator of Y .

The results are presented in Figures 4, and 5, Supplementary Tables S8–S17 and discussed in Section 3.3.

### 3.3. Results

Complete-case analyses and multiple imputation under MAR performed according to established findings (see Figure S1, Tables S3–S11 and Carpenter et al.,^42^ section 1.6). When both outcome and predictor were missing simultaneously, the methods performed similarly, with coverage close to nominal when data were MAR or when the response mechanism depended only on the predictor but not the outcome. For non-monotone missingness, multiple imputation assuming MAR showed poor coverage when the response indicators depended on the incomplete predictor and/or incomplete outcome.

#### 3.3.1. Methods that make distributional assumptions

As Figure 3 (and Supplementary Tables S3–S6) shows, in simulation study 1, maximum likelihood estimation based on the Heckman model while excluding X from the response model, “ML HE X excluded,” had closest to nominal coverage of confidence intervals for the intercept and the parameter for $Z_{1}.$ However, for the parameter for X, imputing X in “MI HE” improved the performance compared to “ML HE X excluded” in Heckman models where the response indicator was positively related to X ( $\gamma_{x}=0.5$ ). The variable X was imputed in “MI HE” assuming its value does not inform the response indicator, and when this assumption held (“Logistic, $\gamma_{x}=0,\gamma_{y}=0.5$ ,” “Logistic, $\gamma_{x}=0,\gamma_{y}=-0.5$ ”), the “MI HE” estimators for X had close to nominal coverage.

Even though it yielded the most biased estimates, random indicator imputation “MI RI” was prone to overcoverage for most of the considered scenarios due to large model standard error, and thus, wide confidence intervals. The empirical standard error was much smaller than the model standard error, suggesting possible bias in the estimation of the model standard error.^43^

All of the methods performed poorly for the complex selection and pattern-mixture models in both simulation studies. “ML HE X excluded” was least stable in terms of variance of the intercept estimator for the pattern-mixture data-generating mechanisms (see Supplementary Table S6).

In simulation study 2, excluding X from the response model, “ML HE X excluded,” had poor coverage for more complex logistic models. Considering complete cases in the partially observed predictor X, “ML HE X cc” generally was better than excluding the partially observed predictor from the response model in “ML HE X excluded.”

Maximum likelihood and multiple imputation based on the Heckman model and complete cases in incomplete predictor (“ML HE X cc,” “MI Heckman X cc”) performed similarly. Imputing X (“MI HE”) slightly improved the coverage of the estimators compared to considering complete cases in the incomplete predictor (“MI HE X cc”).

#### 3.3.2. Methods that require parameter specification

As Figure 3 shows, in simulation study 1, imputation stacking (“MI stacking SP”) that used weights proportional to $\exp\;(-0.5X_{im}-0.5Y_{im})$ regardless of data-generating mechanism performed well when the specified values of the parameters matched their true values (“Logistic, $\gamma_{x}=0.5,\gamma_{y}=0.5$ ”). However, the accuracy of estimation can be significantly compromised when the assumed values differ from the true ones (see e.g. “Logistic, $\gamma_{x}=-0.5,\gamma_{y}=-0.5$ ”) and/or when the models used in the analyses are misspecified. The same holds for “MI NARFCS SP” where the assumed values of the parameters were chosen at random, and the method therefore has low coverage for all considered scenarios.

Imputation stacking had low coverage when the response indicator model was incorrect (“MI Stacking full data”). For example, in data generated by the Heckman models, the response indicator model uses the probit link and relation to outcome Y through a correlation while imputation stacking assumes the logistic link and includes Y as a covariate in the response model. The bias of stacking is more apparent for higher correlations between the model errors (“Heckman, $\gamma_{x}=1,\rho =0.6$ ”).

Methods that used full data to estimate the values of the (sensitivity) parameters, “MI Stacking full data,” “MI NARFCS Shift full data,” “MI NARFCS Interactions full data” were prone to overcoverage of the intercept. This is because, instead of using the true values, they use a sample estimate, which effectively allows the methods to overfit, and so overcover when evaluated across repeated samples. “MI NARFCS Interactions full data” had a tendency to have higher than nominal coverage for the parameters for X and $Z_{1}$ while using a shift only in “MI NARFCS shift only” had closer to nominal coverage.

“MI NARFCS Interactions full data” outperformed all other methods for complex selection and pattern-mixture models.

Simulation study 2 results, shown in Figure 5, follow the results for simulation study 1.

Both imputation stacking assuming MAR X and MNAR Y, “MI Stacking X MAR” and stacking assuming both X and Y MNAR, “MI Stacking full data,” had reasonably good coverage for the parameter for X and $Z_{1},$ while “MI Stacking full data” required specification of multiple models and parameters instead of one parameter in “MI Stacking X MAR.”

As expected, uncertainty intervals had generally low coverage in the simulation studies due to a misspecified selection model (“UI X excluded” in simulation study 1 and for all logistic models) or a wrongly guessed correlation value. Conversely, when the specified correlation value is the same as the true one, as in the scenarios where $R_{x}$ does not depend on data, while $R_{y}$ depends on Y or X,Y in simulation study 2, the UI method had good coverage.

As shown in Supplementary Tables S18 and S19, assuming ρ∈[−0.99,0.99] provides uncertainty intervals with empirical coverage close to 1 for all models except the pattern-mixture models. This is expected since the assumed range for ρ includes a wide range of possible correlation values. Since an uncertainty interval is the union of confidence intervals for considered ranges of the parameter ρ, the resulting intervals are wider than the width of the confidence intervals for all cases.

## 4. Empirical study

We revisit the linear regression analyses of the relationship between longitudinal changes in memory and grey matter volume of the hippocampus, investigated in Gorbach et al.,^4^ using data from the Betula prospective study.^2,3^ A sample of 264 healthy individuals was scheduled to have their memory tested and their grey matter volume measured at the fifth and sixth Betula waves.

Gorbach et al.^4^ investigated the relationship between memory and grey matter changes *via* a linear regression of volume changes on memory decline, age, and hypertension. In Gorbach et al.,^4^ memory decline was estimated by the OLS slope using memory measures from the third to the sixth Betula wave and was thus available for everyone. Here, we estimate the change as the difference in memory scores from the fifth to the sixth Betula waves. We also scale volume change by a factor of 1000 for a clearer representation of regression estimates.

Data for predictor and outcome were missing for 61 out of 264 individuals. Additionally, the predictor alone was missing for three individuals, and the outcome alone was missing for another 46 individuals. Here, we investigate the sensitivity of complete case analyses to various assumptions about the missing data.

We first impute missing data 50 times with 10 iterations, assuming missing at random data. Note that we do not have any secret knowledge to inject (because there is no peeking at the full data). We then use maximum likelihood estimation based on the Heckman selection model and complete cases for the missing predictor, memory change. Maximization does not converge when using the default likelihood maximization method (Newton–Raphson), and ρ is estimated to be close to 1. Therefore, we first estimate the regression parameters using the Broyden–Fletcher–Goldfarb–Shanno likelihood maximization method, then use these estimates as the initial values for maximization using the default method as advised by Toomet and Henningsen.^29^ We construct estimates using multiple imputation based on the Heckman selection model, assuming volume change is MNAR and the conditional models in the fully conditional specification for the predictor are fitted via Bayesian linear regression We also construct uncertainty intervals based on complete cases for memory change and assuming ρ lies within ( − 0.1, 0.1); ( − 0.5, 0.5) and ( − 0.99, 0.99) to investigate the sensitivity of the change-change relationship to different strengths of the relationship between missingness and volume change. We continue with random indicator imputation.

For the imputation using NARFCS, we investigate how the memory-volume change relationship alters if the missing volume and memory changes are, on average, up to two standard deviations lower than the observed changes. To do this, we first assume that the observed and missing parts of the distribution of memory change, conditional on other variables and the response indicator for volume change, differ only by a shift. The same shift-only assumption is imposed for the distribution of volume change. We fit the analysis model over a grid of shift values: {0,−1,−2,−3,−4} for memory change and {0,−5,−10,−20,−40} for volume change, which amounts approximately up to two standard deviations difference between the marginal means of the observed and missing memory and volume change (see Table 3).

**Table 2.** Point estimates (SE, *p*-value) or uncertainty intervals for the regression parameters from the sensitivity analyses in the empirical study.

Method	Intercept	Memory change	Age	Hypertension
Complete-case	1093.57(22.24;0.000)	2.50(0.94;0.009)	−1.71(0.35;0.000)	−6.80(5.22;0.195)
MI under MAR	1099.07(24.14;0.000)	2.53(0.97;0.011)	−1.80(0.38;0.000)	−7.13(5.12;0.167)
ML HE	1093.77(23.13;0.000)	2.49(0.93;0.008)	−1.71(0.40;0.000)	−6.81(5.19;0.191)
MI HE	1100.15(22.07;0.000)	2.01(0.67;0.003)	−1.83(0.39;0.000)	−6.95(4.12;0.095)
MI RI	1100.39(27.82;0.000)	2.18(1.61;0.185)	−1.84(0.55;0.002)	−6.27(5.91;0.291)
UI ρin (−0.1,0.1)	(1048.42;1138.72)	(0.64;4.36)	(−2.43;−0.98)	(−17.18;3.59)
UI ρ in (−0.5;0.5)	(1040.19;1146.94)	(0.50;4.49)	(−2.63;−0.78)	(−18.17;4.58)
UI ρ in (−0.99;0.99)	(1019.69;1167.44)	(0.02;4.97)	(−3.09;−0.33)	(−21.26;7.67)

MI under MAR: Multiple imputation assuming MAR; ML HE: Maximum likelihood estimation based on the Heckman selection model; MI HE: Multiple imputation under the Heckman selection model; MI RI: Random Indicator imputation; UI: uncertainty intervals..

**Table 3.** Point estimates (SE, *p*-value) for the regression parameters from the sensitivity analyses in the empirical study according to imputations using NARFCS.

Shift memory	Marginal diff. memory	Shift volume	Marginal diff. volume	Intercept	Memory change	Age	Hypertension
0	−0.9	0	−24.9	1106.09(23.46;0.00)	3.04(1.40;0.04)	−2.00(0.38;0.00)	−5.36(5.33;0.32)
−1	−1.7	0	− 20.3	1103.21(22.43;0.00)	3.43(1.23;0.01)	− 1.90(0.36;0.00)	− 6.93(5.63;0.22)
− 2	− 2.7	0	− 23.4	1099.28(23.47;0.00)	3.80(1.26;0.00)	− 1.84(0.38;0.00)	− 7.37(5.15;0.16)
− 3	− 3.8	0	− 20.3	1094.04(22.68;0.00)	3.58(1.58;0.03)	− 1.74(0.36;0.00)	− 5.97(5.44;0.27)
− 4	− 4.7	0	− 23.7	1095.06(23.80;0.00)	3.88(1.49;0.01)	− 1.75(0.37;0.00)	− 5.95(5.47;0.28)
0	− 0.8	-5	− 27.1	1109.14(24.24;0.00)	2.95(1.21;0.02)	− 2.06(0.39;0.00)	− 6.12(5.20;0.24)
− 1	− 1.6	-5	− 26.0	1105.53(24.78;0.00)	3.32(1.16;0.01)	− 1.97(0.41;0.00)	− 7.05(5.80;0.23)
− 2	− 2.8	-5	− 28.1	1101.51(22.75;0.00)	4.07(1.49;0.01)	− 1.90(0.36;0.00)	− 5.17(5.14;0.32)
− 3	− 3.8	-5	− 27.5	1099.90(22.17;0.00)	4.04(1.37;0.01)	− 1.85(0.35;0.00)	− 6.42(5.59;0.25)
− 4	− 4.8	-5	− 28.6	1096.88(22.90;0.00)	4.12(1.27;0.00)	− 1.79(0.36;0.00)	− 6.89(5.64;0.23)
0	− 0.7	-10	− 33.2	1110.15(27.54;0.00)	2.88(1.19;0.02)	− 2.11(0.44;0.00)	− 6.41(5.49;0.25)
− 1	− 1.7	-10	− 31.1	1107.87(22.81;0.00)	3.45(1.37;0.02)	− 2.04(0.36;0.00)	− 5.72(5.82;0.33)
− 2	− 2.7	-10	− 32.3	1105.72(25.58;0.00)	4.06(1.48;0.01)	− 1.98(0.41;0.00)	− 6.66(5.53;0.23)
− 3	− 3.8	-10	− 33.1	1099.45(24.50;0.00)	4.48(1.70;0.01)	− 1.87(0.38;0.00)	− 6.45(5.58;0.25)
− 4	− 4.6	-10	− 30.3	1094.47(21.72;0.00)	4.09(1.61;0.02)	− 1.78(0.34;0.00)	− 5.96(5.51;0.28)
0	− 0.7	20	− 42.5	1117.96(26.28;0.00)	3.07(1.39;0.03)	− 2.28(0.43;0.00)	− 7.42(6.31;0.24)
− 1	− 1.6	20	− 40.2	1108.78(22.99;0.00)	3.90(1.63;0.02)	− 2.10(0.37;0.00)	− 6.08(5.38;0.26)
− 2	− 2.6	20	− 40.6	1107.08(21.93;0.00)	4.23(1.54;0.01)	− 2.05(0.35;0.00)	− 6.42(5.75;0.27)
− 3	− 3.7	20	− 43.0	1100.66(26.90;0.00)	4.92(1.63;0.01)	− 1.93(0.43;0.00)	− 6.50(5.42;0.23)
− 4	− 4.7	20	− 41.8	1099.73(23.60;0.00)	4.82(1.52;0.00)	− 1.90(0.37;0.00)	− 5.76(5.43;0.29)
0	− 0.7	40	− 63.8	1132.41(29.01;0.00)	3.06(1.71;0.08)	− 2.62(0.46;0.00)	− 7.15(6.35;0.26)
− 1	− 1.8	40	− 64.8	1121.95(27.09;0.00)	4.69(1.86;0.02)	− 2.42(0.43;0.00)	− 6.28(6.64;0.35)
− 2	− 2.7	40	− 63.9	1116.42(26.55;0.00)	5.35(1.70;0.00)	− 2.30(0.42;0.00)	− 6.49(6.42;0.31)
− 3	− 3.8	40	− 63.3	1106.95(24.55;0.00)	6.12(1.80;0.00)	− 2.11(0.39;0.00)	− 6.44(6.10;0.29)
− 4	− 4.7	40	− 61.3	1103.41(26.39;0.00)	5.97(1.86;0.00)	− 2.03(0.41;0.00)	− 6.78(5.78;0.24)

The mean observed memory change is − 1.18 points, the standard deviation is 2.51 points. The mean observed hippocampal grey matter change is 977.7 points, the standard deviation is 32.88 points. Marginal diff.: the estimated mean of missing subtracted from the mean of the observed changes..

For imputation stacking, we first consider only the regression outcome, volume change, to be MNAR, and the predictor, memory change, to be missing at random. Assuming logistic regression for the response indicator for volume change, we only have to specify one parameter, the coefficient γ for volume change in this regression (see Section 2.5 in Beesley and Taylor^13^). Since we expect that those with a larger absolute value of volume change, that is, smaller grey matter shrinkage, are more prone to having their grey matter volume observed, the parameter should have positive values. Next, we have to decide on the scale for the parameter. Due to assumed logistic regression, the weights are proportional to exp(−γvolume change) .^13^ Observed volume changes are between 850 and 1,070. Therefore, we select small values 0.001, 0.005, 0.01, 0.02, 0.03 for the parameter γ , so that exp(−γvolume change) are not extremely close to 0. Since the theory for calculating the *p*-values for imputation stacking is lacking, we present in Table 4 point estimates and 95% confidence intervals assuming a normal distribution approximation as point estimate ± 1.96 × StackImpute jackknife modification of the standard error estimate.

**Table 4.** Point estimates (confidence intervals) for the regression parameters in the empirical study from the sensitivity analyses according to imputation stacking when assuming only volume change to be MNAR; γ : the coefficient for volume change in the logistic regression for the response indicator for volume change given volume change, memory change, age, and hypertension.

γ	Intercept	Memory change	Age	Hypertension
0.001	1099.51(1052.13,1146.89)	2.53(0.59,4.46)	−1.81(−2.55,−1.07)	−7.11(−17.18,2.95)
0.005	1101.41(1054.33,1148.50)	2.53(0.59,4.47)	−1.86(−2.60,−1.12)	−7.12(−17.46,3.22)
0.01	1103.74(1056.07,1151.41)	2.55(0.56,4.53)	−1.92(−2.68,−1.17)	−7.13(−17.95,3.69)
0.02	1108.26(1054.88,1161.64)	2.60(0.32,4.87)	−2.04(−2.90,−1.19)	−7.19(−19.44,5.07)
0.03	1112.59(1045.64,1179.55)	2.66( − 0.19,5.51)	−2.16(−3.26,−1.06)	−7.23(−21.65,7.18)

We then construct imputation stacking estimates assuming both volume and memory change are MNAR. Because this requires a large number of models, we adopt several simplifying assumptions. Results are provided in Tablentary S20. All analyses within empirical study were performed using R Statistical Software, version 4.5.2.^44^

For most of the considered models (see Tables 2 to 4), the relationship between the brain and memory changes remains significant, reinforcing earlier evidence from aging studies. The relationship between brain and memory changes is nonsignificant at the 5% significance level for the random indicator method and NARFCS when the difference between the mean of observed and missing memory change is small (assuming a conditional shift of 0), while the missing hippocampal grey matter volume changes are on average more than one standard deviations lower than the mean of the observed changes (assuming a shift of volume to be − 40). Since we expect missing volume and memory changes to be correlated, scenarios in which the missing volume is much lower than the observed volume, but the missing memory change is close to the observed memory change, are unlikely. Table 4 shows that for most values of the parameter γ, the relationship between the memory and brain changes is significant on the 5% significance level as the confidence intervals do not include 0. When γ=0.03, the results are driven by a few imputed values with weights much higher than for other imputations, are presented here for demonstration and should be taken with a pinch of salt. Here, one imputed value has a weight greater than 0.4 while the majority of weights are below 0.1 (see Supplementary Figure S4).

## 5. Discussion

We compared approaches for linear regression analyses when data are MNAR in both outcomes and predictors. The methods evaluated were complete-case analysis; multiple imputation assuming missing at random; maximum likelihood estimation, uncertainty intervals, and multiple imputation based on the Heckman selection model; multiple imputation using imputation stacking, not-at-random fully conditional specification, and random indicator imputation.

Methods can be grouped in various ways. First, maximum likelihood estimation, uncertainty intervals, MI based on the Heckman model, and imputation stacking are based on the selection factorization of the full-data distribution; NARFCS and random indicator imputation adopt the pattern-mixture specification.

Second, MI methods such as imputation stacking, NARFCS, and random indicator imputation are readily applicable to multivariate MNAR data. Other methods were primarily designed for a single MNAR variable (the outcome). Extending them to multivariate MNAR often requires pragmatic workarounds, such as subsetting the sample to those with complete predictors (which collapses to complete-case analysis and is impractical when outcomes and predictors are missing for the same cases), omitting incomplete predictors from the response indicator model (for uncertainty intervals and maximum likelihood estimation based on the Heckman selection model; induces model misspecification), or imputing incomplete predictors under MAR assumption (for MI based on the Heckman model). Such practical adjustments can undermine performance.

Third, maximum likelihood and MI based on the Heckman model and random indicator imputations are the methods that make distributional assumptions about the full data model, and the user does not have to specify any parameters. This may be a positive feature in making the analyst’s life easier, but places substantial weight on unverifiable distributional assumptions. In contrast, imputation using stacking, NARFCS, and uncertainty intervals, require the analyst to specify parameters’ values explicitly. This requires the analyst to engage more with missing data assumptions, and it is the parameters that do the heavy lifting.

Among methods not requiring user-specified parameters’ values, random indicator imputation was least stable, exhibiting high bias and overestimation of standard errors relative to alternatives. We consider our simulation results sufficiently conclusive to rule out the use of this method in future methodological work. Within the Heckman model-based methods, misspecification of the response model by omitting the incomplete predictor yielded the poorest estimates under some designs some of the models. Maximum likelihood and multiple imputation restricted to complete predictors performed similarly, while multiple imputation based on the Heckman model and imputing incomplete predictor under MAR improved performance. Current software for MI based on the Heckman model allows imputation of multiple MNAR variables, but is validated primarily for MNAR outcomes, caution is therefore advised (see the miceMNAR R package documentation). Future research should evaluate the method’s performance when incomplete predictors are imputed under the MNAR assumption as well. Notably, MI under the Heckman selection model had the longest computation time among all considered methods.

As anticipated, the methods that need parameter values specification perform well when the models were correctly specified and, where applicable, the specified parameters’ values matched their true values. However, the accuracy of estimation degraded and could be even worse than the complete-case analyses when the assumed values differ from the true ones and/or when the models were misspecified.

Imputations using NARFCS (with sensitivity parameters informed by the full data structure) provided the most consistently reliable results overall, closely followed by imputation stacking. Although NARFCS uses pattern-mixture specification, it also performed well for data generated from selection models. Within NARFCS, assuming the difference between the observed and missing parts of the fully conditional imputation models only in the intercept (the shift approach) achieved near-nominal coverage, whereas allowing shifts across all coefficients (the interaction approach) tended to overcover due to the sneak peaking and in-sample overfitting. However, the latter specification outperformed all other methods when data was generated from the pattern-mixture models.

The challenge for both imputation stacking and NARFCS is parameter specification. For NARFCS, one must define the differences between conditional distributions of missing data given other variables in the model. Correctly specifying these differences based on conditional models can be difficult, and calibration techniques for eliciting plausible values have been proposed by Tompsett et al.^14^ Imputation stacking under multivariate MNAR is more challenging than just using mice as in other methods, as data must be imputed from different distributions for different missing data patterns. Additionally, the required weighting function becomes increasingly complex as the number of MNAR variables increases. For example, for four missing data patterns in the case of non-monotone MNAR missing in two variables, the user has to specify six response indicator models! Practical strategies to reduce this complexity include adopting simpler assumptions, such as assuming MAR predictor to simplify weighting, or using approximate weight construction, as described by Beesley and Taylor,^13^ rather than implementing the exact approach.

In summary, our findings lead to several practical recommendations. First, analysts should exercise caution with random indicator imputation, as our simulations revealed substantial bias and issues with variance estimation. Second, when there is no strong indication for a particular full-data model, we recommend conducting sensitivity analyses that span both selection and pattern-mixture model classes and explore a range of plausible parameter values. Where feasible, specifications that minimize overfitting risk, such as intercept-only shifts in NARFCS, are preferable. Third, careful specification of the (sensitivity) parameters’ values is essential for meaningful sensitivity analyses. This is often challenging, and practical strategies include (a) simplifying the assumptions to assume that only key variable(s) are MNAR and (b) looking at the sensitivity of conclusions across a range of plausible values, where the plausible values can be deduced using calibration. Option (b) can lead to a “tipping point” analysis identifying parameter values extreme enough to alter conclusions. It is worth noting here that, when the goal is to estimate an exposure or treatment effect and outcomes are missing, sensitivity analyses assuming opposite-direction bias between groups (e.g. missing outcomes lower than the observed in the treated group but higher in the control group) are more likely to influence effect estimates than shifts in the same direction across groups. Analysts should prioritize these considerations to ensure sensitivity analyses are both rigorous and practically informative.

## Supplemental Material

sj-pdf-1-smm-10.1177_09622802261458074 - Supplemental material for A comparison of missing data approaches for linear regression with missing not at random outcome and predictors Supplemental material, sj-pdf-1-smm-10.1177_09622802261458074 for A comparison of missing data approaches for linear regression with missing not at random outcome and predictors by Tetiana Gorbach, Tim P Morris and James R Carpenter in Statistical Methods in Medical Research
