# Supplementary material for: A comparison of missing data approaches for linear regression with missing not at random outcome and predictors
Source: Stat Methods Med Res. 2026 Jul 15;35(8):1658–77. doi: 10.1177/09622802261458074 (PMC13478446; doi:10.1177/09622802261458074)
Supplement: Supplementary file 1 [file sj-pdf-1-smm-10.1177_09622802261458074.pdf]

Supplementary Material to “A comparison of missing data approaches for linear regression with missing not at random outcome and predictors”

S1 Software implementations of considered methods

The available software implementations of the considered methods in `R`, `Stata` and `SAS` are summarized in Table S1.

Method	R package	Stata	SAS
Complete cases	default	default	default
MI MAR	`mice`, (1) see details below	`mi impute`	`PROC MI`
ML HE	`sampleSelection`,(2)	`heckman`	`PROC QLIM`
UI	`ui`,(3)	-	-
MI HE	`mice`, `miceMNAR`, (4) see details below	-	-
MI stacking	`StackImpute`, (5) see details below	-	-
MI NARFCS	`mice`, (6) see details below	`mi impute`	-
MI RI	`mice`, (7) see details below	-	-

Table S1: Software implementations. “-” means no implementation available yet. MI MAR: Multivariate multiple imputation assuming MAR; ML HE: Maximum likelihood estimation based on the Heckman selection model, UI: uncertainty intervals; MI HE: Multiple imputation under the Heckman selection model; MI stacking: Multiple imputation using imputation stacking; MI NARFCS: Multiple imputation using not-at-random fully conditional specification, MI RI: random indicator imputation.

A more detailed description of the software implementation of selected multiple imputation methods in R within the simulation studies is provided below.

Multiple imputation assuming MAR (MI MAR)

The MICE (multivariate imputation using chained equations) algorithm, as implemented in `mice` R package,(1) is the most commonly used method for generating multiple imputations for multivariate, non-monotone missingness, as outlined in Section 4.5.1 of van Buuren and Groothuis-Oudshoorn.(8) The MICE algorithm employs fully conditional specification (9; 10) to estimate the posterior predictive distribution. It iteratively imputes missing values one variable at a time using the conditional distribution of that variable given all other variables (imputation model). The user must specify, among other things, an imputation model for each variable with missing data, which variables to use as predictors in this imputation model, and the imputation method. In the simulation studies, we impute both Y and X using Bayesian linear regression by specifying `method = "norm"`

in the `mice` function of the `mice` R package.(1) We include Z_2 in the imputation model as an auxiliary variable.

Maximum likelihood estimation under the Heckman selection model

Simulation study 1: Heckman selection model estimated *via* maximum likelihood using the `selection` function of `sampleSelection` R package.(2) The package states "NA-s are allowed in the data. These are ignored if the corresponding outcome is unobserved; otherwise observations that contain NA (either in selection or outcome) are removed". Thus, if X and Y are missing for the same observations as in simulation study 1, only complete cases are retained, and the selection model's outcome has only the value 1 for all units in the data. As a result, the estimation of the Heckman selection model is infeasible. Therefore, it becomes necessary to exclude X from the selection model in the `selection` function of `sampleSelection` R package, which leads to the selection model misspecification. We refer to this method as "ML HE X excluded".

In simulation study 2, the difference between "ML HE X cc" and "ML HE X excluded" is in the estimation of the selection model. "ML HE X cc" used cases with the observed X, while "ML HE X excluded" also included the cases with both X and Y unobserved. Thus "ML HE X excluded" used more cases to estimate the inverse Mills ratio, but ignored the relation between the response indicator for R_y and X in the selection model. Both methods use only the cases with observed X and Y to estimate the outcome regression.

Multiple imputation under the Heckman selection model

To multiply impute an MNAR variable using the Heckman selection model, one can use the `mice` function of `mice` R package: first, one should specify selection and outcome equations for this variable using `generate_JointModelEq` function of `miceMNAR` R package;(4) then one must define the parameters `data, method, predictorMatrix` and `JointModelEq` using `MNARargument` function of `miceMNAR` R package and adjust the corresponding parameters in the `mice` function of `mice` R package. The variable is then imputed using `mice.impute.heckprob` or `mice.impute.hecknorm` imputation method in `mice`, depending on the variable's type. At the time of writing, the package `miceMNAR` was removed from CRAN by administrators "as email to the package maintainer was undeliverable" (`https://cran.r-project.org/web/packages/miceMNAR/index.html`). However, it can be installed from the GitHub repository `https://github.com/cran/miceMNAR`. Note that the package relies on `GJRM` R package, version 0.2-6.7; running `mice.impute.hecknorm` function with other `GJRM` version might result in errors due to incompatibilities with `copulaSampleSel` function of `GJRM` R package.

Multiple imputation under the Heckman selection model (MI HE) is implemented by specifying the method `mice.impute.hecknorm` when imputing Y using `mice` function of the `mice` R package. The missing values in X are imputed using Bayesian linear regression

using `method = "norm"` in `mice` function, assuming that the response indicator does not depend on X (selection model is misspecified in these analyses). We specify that Z_2 should be used in the imputations by including it in the `data` argument. In `JointModelEq,` we state that X, Z_1 are the predictors in the outcome model, and X, Z_1, Z_2 are the predictors in the selection model. When applying this method in simulation study 1, the indicator of missingness in Y is excluded from the imputation model for X since it is the same as the indicator for missingness in X.

Uncertainty intervals

In simulation study 2, the "UI X excluded" method, which excludes X from the selection model, results in an error. This occurs because the `ui` implementation involves a matrix product between the inverse Mills ratio vector with a length equal to the number of observed Y values, and the outcome model's matrix, which only includes rows where both X and Y are observed (in simulation study 1 the estimation was possible since X and Y were missing for the same cases).

Imputation stacking

A user should implement imputations under MAR; standard error estimation is implemented in the R package `StackImpute`.(5)

In simulation study 1 when Y and X are missing simultaneously, following the logic of Appendix A.3 in Beesley and Taylor,(5), we have that

$$f(Y, X|Z_1, Z_2, R = 0) \propto f(Y, X|Z_1, Z_2, R = 1)\frac{P(R = 0|X, Y, Z_1, Z_2)}{1 - P(R = 0|X, Y, Z_1, Z_2)}.$$

Let $\{X_{im}, Y_{im}\}$ denote the mth imputation of $\{X_i, Y_i\}$. The weights are then defined as $\omega_{im} \propto \frac{P(R_i=0|X_{im},Y_{im},Z_{1i},Z_{2i})}{1-P(R_i=0|X_{im},Y_{im},Z_{1i},Z_{2i})}$ that are scaled such that $\sum_{m=1}^{M} \omega_{im} = 1$. The weights are equal to $1/M$ for cases with observed Y and X.

In simulation study 2, "MI Stacking full data", uses imputations provided by the "MI MAR" method from $f(X|Y, Z_1, Z_2, R_x = 1)$ and $f(Y|X, Z_1, Z_2, R_y = 1)$ for those cases with either X or Y missing. To impute from $f(X, Y|Z_1, Z_2, R_x = 1, R_y = 1)$ for the cases with X and Y missing, we use a subset of the observed data that contains only cases with X and Y missing simultaneously or both observed and `method = "norm"` in the `mice` function of the `mice` R-package. We assume logistic regressions for response data indicators and define the weights using the predictions from the fit of the corresponding logistic regressions to the full data.

Specifying all the models and sensitivity parameters when imputing from different distributions for different missing data patterns can be challenging. Therefore, in "MI Stacking

X MAR", we assume that only the regression outcome is missing not at random. Here, we stack imputations from "MI MAR". Assuming a logistic model for the response indicator for the analysis model outcome, only the coefficient associated with outcome in this logistic model should be specified. We use full data to estimate this parameter.

Imputation using NARFCS

MI NARFCS, Multiple imputation using not-at-random fully conditional specification, is implemented in "`mnar.norm`" and "`mnar.logreg`" imputation methods in the `mice` R package using functions `mice.impute.mnar.norm` and `mice.impute.mnar.logreg`. Note that R will automatically remove variables if their regression parameters for the imputation model cannot be estimated (unless specified as sensitivity parameters). So, one has to check if any missing indicators have been removed. Another implementation is provided by Moreno-Betancur *et al.*(11) It also uses `mice` function of the `mice` R package but specifies methods "`normSens`" and "`logregSens`" for imputations of continuous or binary variables, respectively.

In simulation study 1, multiple imputation using not-at-random fully conditional specification implemented by specifying `method = "mnar.norm"` for imputations of Y and X and adjusting the parameter `blots` in `mice` function of `mice` R package. We implement three NARFCS methods.

In "MI NARFCS Shift SP", we assume that those with missing Y or X have twice as large intercept as those with the observed Y or X, conditional on other variables in the data: $E(X|Y, Z_1, Z_2, R) = \mu_0+\mu_1 Y+\mu_2 Z_1+\mu_3 Z_2+\mu_0(1-R)$ and $E(Y|X, Z_1, Z_2, R) = \nu_0+\nu_1 X+\nu_2 Z_1+\nu_3 Z_2+\nu_0(1-R)$. To define input values of the shifts μ_0 and ν_0, we fit linear regressions of X on Y, Z_1, Z_2 and of Y on X, Z_1, Z_2 using the observed data to get the estimates $\hat{\mu}_0$ and $\hat{\nu}_0$. This is done in the simulation study to partition issues with elicitation of the shifts from how well the NARFCS machinery can use these sensitivity parameters. The `ums` parameter of `blots` argument in `mice` specifies the difference between the identifiable part, $E(X|Y, Z_1, Z_2, R = 1)$ or $E(Y|X, Z_1, Z_2, R = 1)$, and the missing part, $E(X|Y, Z_1, Z_2, R = 0)$ or $E(Y|X, Z_1, Z_2, R = 0)$, of the univariate conditional models for incomplete variables. This difference is equal to $\mu_0 = E(X|Y, Z_1, Z_2, R = 0) - E(X|Y, Z_1, Z_2, R = 1)$ for X and $\nu_0 = E(Y|X, Z_1, Z_2, R = 0) - E(Y|X, Z_1, Z_2, R = 1)$ for Y. We set this `ums` parameter to $\hat{\mu}_0$ for X and to $\hat{\nu}_0$ for Y.

In "MI NARFCS Shift full data", we assume that the conditional distributions of X and Y for missing data differ from the conditional distribution for the observed data only by a shift in the intercept: $E(X|Y, Z_1, Z_2, R) = \mu_0 + \mu_1 Y + \mu_2 Z_1 + \mu_3 Z_2 + \mu_4(1 - R)$ and ν_4 in $E(Y|X, Z_1, Z_2, R) = \nu_0 + \nu_1 X + \nu_2 Z_1 + \nu_3 Z_2 + \nu_4(1 - R)$. However, now we use full data to estimate the shifts μ_4 and ν_4, which is unrealistic in practice but can be seen as a scenario when the value of the sensitivity parameter is guessed close to the true value of the sensitivity parameter. We then set `ums` parameter to $\hat{\mu}_4$ for X and to $\hat{\nu}_4$ for Y.

In "MI NARCFS Interactions full data" we allow the coefficients of the linear regressions

of X on Y, Z_1, Z_2 and Y on X, Z_1, Z_2 to vary across missingness patterns by including the interaction terms between the variables and the response indicator. $E(X|Y, Z_1, Z_2, R) = \mu_0 + \mu_1 Y + \mu_2 Z_1 + \mu_3 Z_2 + (\mu_4 + \mu_5 Y + \mu_6 Z_1 + \mu_7 Z_2)(1 - R)$ and $E(Y|X, Z_1, Z_2, R) = \nu_0 + \nu_1 X + \nu_2 Z_1 + \nu_3 Z_2 + (\nu_4 + \nu_5 X + \nu_6 Z_1 + \nu_7 Z_2)(1 - R)$. Here, we again unrealistically use full data to estimate the values of the coefficients for the interaction terms. Note that this is slightly different from using the true values. We then set `ums` parameter to $\hat{\mu}_4 + \hat{\mu}_5 Y + \hat{\mu}_6 Z_1 + \hat{\mu}_7 Z_2$ for X and to $\hat{\nu}_4 + \hat{\nu}_5 X + \hat{\nu}_6 Z_1 + \hat{\nu}_7 Z_2$ for Y.

Note here that the NARFCS procedure of Tompsett *et al.* (6) considers including missing indicators for all incomplete variables in the fully conditional distributions. However, since X and Y are missing for the same cases in simulation study 1, missing indicators for X and Y are equal.

In simulation study 2,"MI NARFCS Shift full data" considers $E(X|Y, Z_1, Z_2, R_x, R_y) = \mu_0 + \mu_1 Y + \mu_2 Z_1 + \mu_3 Z_2 + \mu_4 R_y + \mu_5(1 - R_x)$ and $E(Y|X, Z_1, Z_2, R_x, R_y) = \nu_0 + \nu_1 X + \nu_2 Z_1 + \nu_3 Z_2 + \nu_4 R_x + \nu_5(1 - R_y)$. We set `ums` parameter to $\hat{\mu}_5$ for X and to $\hat{\nu}_5$ for Y estimated from the full data.

In "MI NARCFS Interactions full data" within simulation study 2, we consider $E(X|Y, Z_1, Z_2, R_x, R_y) = \mu_0 + \mu_1 Y + \mu_2 Z_1 + \mu_3 Z_2 + \mu_4 R_y + (\mu_5 + \mu_6 Y + \mu_7 Z_1 + \mu_8 Z_2 + \mu_9 R_y)(1 - R_x)$ and $E(Y|X, Z_1, Z_2, R_x, R_y) = \nu_0 + \nu_1 X + \nu_2 Z_1 + \nu_3 Z_2 + \nu_4 R_x + (\nu_5 + \nu_6 X + \nu_7 Z_1 + \nu_8 Z_2 + \nu_9 R_x)(1 - R_y)$. We then set `ums` parameter to $\hat{\mu}_5 + \hat{\mu}_6 Y + \hat{\mu}_7 Z_1 + \hat{\mu}_8 Z_2 + \hat{\mu}_9 R_y$ for X and to $\hat{\nu}_5 + \hat{\nu}_6 X + \hat{\nu}_7 Z_1 + \hat{\nu}_8 Z_2 + \hat{\nu}_9 R_x$ for Y, where the estimates are constructed using the full data.

Random indicator imputation (MI RI)

Random indicator imputation is implemented in the `mice` R package using `"ri"` imputation method in `mice` function.

S2 Simulation study 1 results

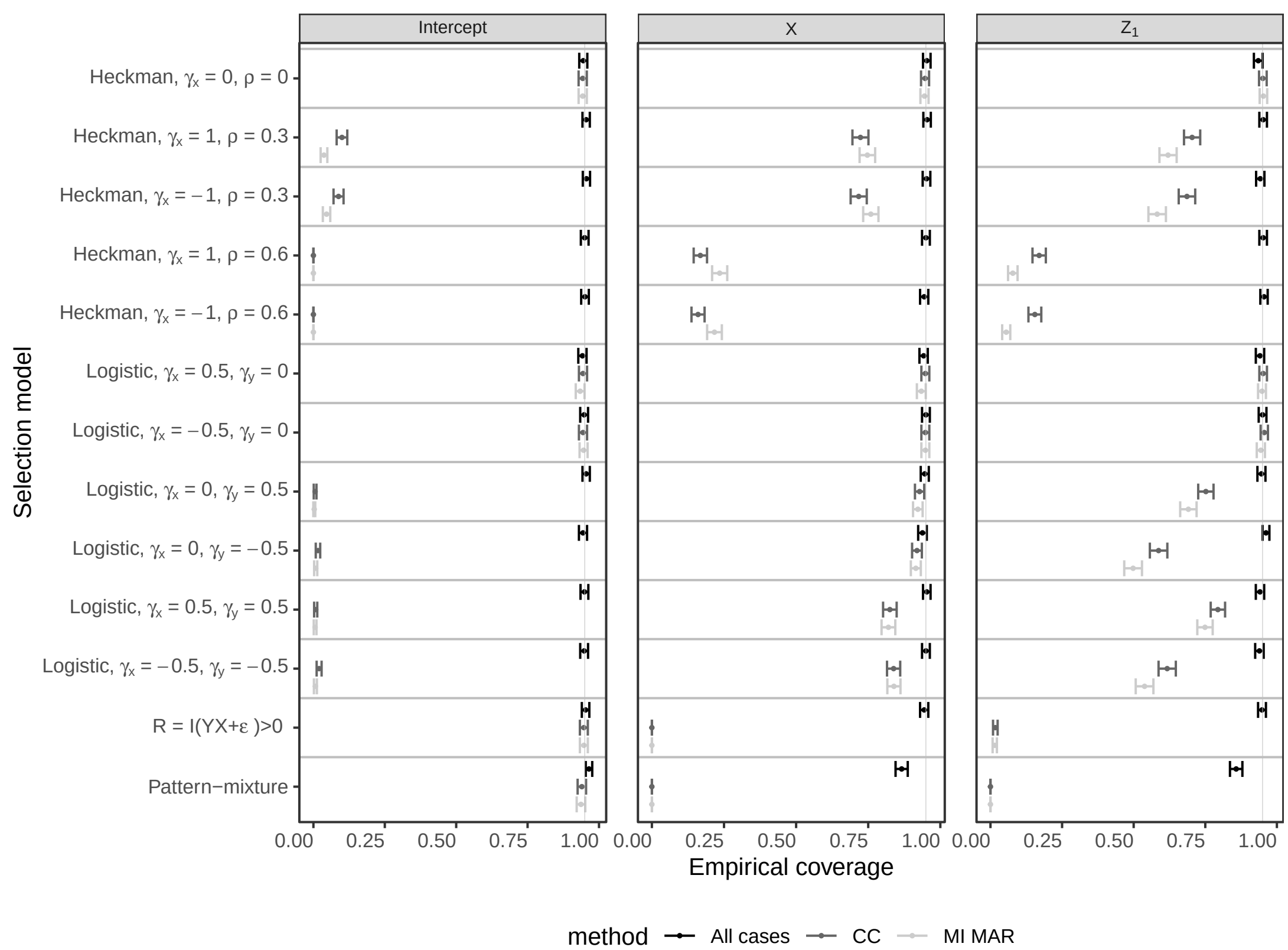

Figure S1: Empirical coverage of 95% confidence intervals in simulation study 1 where X and Y are missing for the same participants for all cases, complete cases (CC), multiple imputation under MAR (MI MAR). The whiskers represent the Monte Carlo standard error of the coverage estimate.

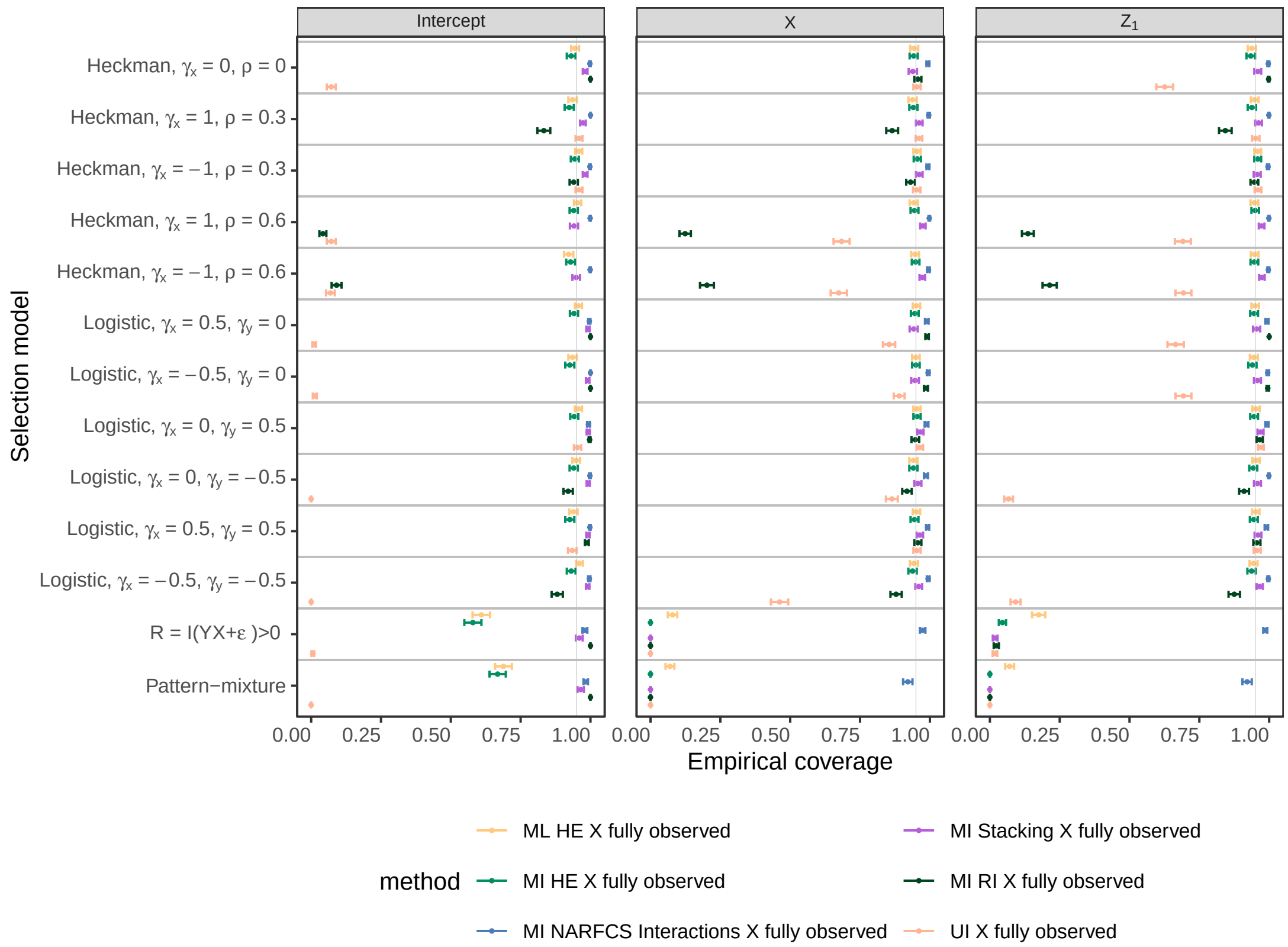

Figure S2: Empirical coverage for some methods in simulation study 1 when X is assumed to be fully observed while Y is missing for some cases in the data. The whiskers represent the Monte Carlo standard error of the coverage estimate. Generally and unsurprisingly, methods perform well in the situations they were designed for. Maximum likelihood estimation and multiple imputation based on the Heckman selection model ("ML HE X fully observed" and 'MI HE X fully observed") had close to nominal coverage for all Heckman but also logistic selection models. Imputation using NARFCS, "MI NARFCS Int. X full", tended to overcover the parameters; imputation stacking "MI Stacking X full" was less susceptible to overcoverage than imputation using NARFCS. Random indicator imputation, however, had low coverage for all of the parameters and some Heckman and logistic models. Uncertainty intervals have close to nominal coverage when the assumed model is correct ("Heckman, $\gamma_x = -1, \rho = 0.3$", "Heckman $\gamma_x = -1, \rho = 0.3$"). For the complex selection and the pattern-mixture model, only NARFCS had an acceptable coverage of regression parameters for X and Z_1.

All Cases	all cases analysis
CC	complete-case analysis
MI MAR	multiple imputation using MAR
ML HE excl.	maximum likelihood estimation using the Heckman selection model, excluding X from the selection equation
ML HE X excluded	maximum likelihood estimation using the Heckman selection model, excluding X from the selection equation
ML HE X fully obs.	maximum likelihood estimation based on the Heckman selection model, assuming X fully observed
ML HE X cc	maximum likelihood estimation based on the Heckman selection model, restricted to cases with observed X
UI X excl.	uncertainty intervals excluding X from the selection equation
UI X fully obs.	uncertainty intervals assuming fully observed X
UI X cc	uncertainty intervals restricted to cases with observed X
MI HE	multiple imputation under the Heckman selection model
MI HE X fully obs.	multiple imputation under the Heckman selection model assuming X fully observed
MI HE X cc	multiple imputation under the Heckman selection model restricted to cases with observed X
Stacking SP	MI Stacking, weights proportional to $\exp(-(0.5X_{im} + 0.5Y_{im}))$
Stacking full data	MI Stacking full data
Stacking X fully obs.	MI stacking assuming fully observed X
Stacking X MAR	MI stacking assuming MAR X
NARFCS Shift SP	MI NARFCS assuming that those with missing Y or X have twice as large an intercept as those with the observed Y or X
NARFCS Shift full data	MI NARFCS Shift full data
NARFCS Int. full data	MI NARFCS Interactions full data
NARFCS Int. X fully obs.	MI NARFCS Interactions assuming fully observed X
MI RI	Random indicator imputation
MI RI X fully obs.	Random indicator imputation assuming fully observed X

Table S2: Abbreviations and corresponding analysis methods used in the simulation studies

		Intercept					β_x					β_z				
DGM	Method	bias	empse	rmse	modse	cov	bias	empse	rmse	modse	cov	bias	empse	rmse	modse	cov
Heckman, γ_x=0, ρ=0	All cases	0.00	0.03	0.03	0.03	0.95	0.00	0.03	0.03	0.03	0.95	-0.00	0.03	0.03	0.03	0.94
Heckman, γ_x=0, ρ=0	CC	-0.00	0.05	0.05	0.05	0.94	-0.00	0.05	0.05	0.04	0.95	-0.00	0.05	0.05	0.05	0.95
Heckman, γ_x=0, ρ=0	MI MAR	0.00	0.06	0.06	0.06	0.94	0.00	0.05	0.05	0.05	0.95	-0.00	0.05	0.05	0.05	0.95
Heckman, γ_x=0, ρ=0	ML HE X excl.	-0.00	0.09	0.09	0.09	0.94	-0.00	0.05	0.05	0.04	0.95	-0.00	0.06	0.06	0.06	0.94
Heckman, γ_x=0, ρ=0	ML HE X fully obs.	-0.00	0.09	0.09	0.09	0.95	-0.00	0.05	0.05	0.04	0.94	-0.00	0.06	0.06	0.06	0.94
Heckman, γ_x=0, ρ=0	UI X excl.	-0.18	0.05	0.18	0.05	0.07	-0.00	0.05	0.05	0.05	0.95	-0.08	0.05	0.10	0.05	0.63
Heckman, γ_x=0, ρ=0	UI X fully obs.	-0.18	0.05	0.18	0.05	0.07	-0.00	0.05	0.05	0.05	0.95	-0.08	0.05	0.10	0.05	0.63
Heckman, γ_x=0, ρ=0	MI HE	-0.00	0.09	0.09	0.08	0.91	0.00	0.05	0.05	0.05	0.95	-0.00	0.06	0.06	0.06	0.93
Heckman, γ_x=0, ρ=0	MI HE X fully obs.	-0.00	0.09	0.09	0.09	0.93	-0.00	0.05	0.05	0.05	0.94	-0.00	0.06	0.06	0.06	0.93
Heckman, γ_x=0, ρ=0	Stacking SP	-0.21	0.06	0.22	0.06	0.07	0.04	0.05	0.07	0.06	0.92	-0.10	0.05	0.11	0.06	0.59
Heckman, γ_x=0, ρ=0	Stacking full data	0.00	0.05	0.05	0.06	0.99	0.00	0.05	0.05	0.05	0.95	-0.00	0.05	0.05	0.05	0.96
Heckman, γ_x=0, ρ=0	Stacking X fully obs.	0.00	0.04	0.04	0.06	0.98	-0.00	0.05	0.05	0.05	0.94	-0.00	0.05	0.05	0.05	0.96
Heckman, γ_x=0, ρ=0	NARFCS Shift SP	0.00	0.09	0.09	0.06	0.81	-0.00	0.05	0.05	0.05	0.95	-0.00	0.06	0.06	0.05	0.92
Heckman, γ_x=0, ρ=0	NARFCS Shift full data	0.00	0.04	0.04	0.06	0.99	-0.00	0.05	0.05	0.05	0.95	-0.00	0.05	0.05	0.05	0.96
Heckman, γ_x=0, ρ=0	NARFCS Int. full data	0.00	0.03	0.03	0.06	1.00	0.00	0.04	0.04	0.05	0.98	-0.00	0.03	0.03	0.05	1.00
Heckman, γ_x=0, ρ=0	NARFCS Int. X fully obs.	0.00	0.03	0.03	0.06	1.00	0.00	0.03	0.03	0.05	0.99	-0.00	0.03	0.03	0.05	1.00
Heckman, γ_x=0, ρ=0	MI RI	-0.06	0.10	0.12	0.26	1.00	-0.01	0.05	0.05	0.07	0.99	-0.03	0.06	0.07	0.13	1.00
Heckman, γ_x=0, ρ=0	MI RI X fully obs.	-0.06	0.11	0.12	0.26	1.00	-0.00	0.05	0.05	0.05	0.96	-0.03	0.07	0.07	0.13	1.00
Heckman, γ_x=1, ρ=0.3	All cases	-0.00	0.03	0.03	0.03	0.96	0.00	0.03	0.03	0.03	0.95	0.00	0.03	0.03	0.03	0.95
Heckman, γ_x=1, ρ=0.3	CC	0.17	0.05	0.18	0.05	0.10	-0.07	0.05	0.08	0.05	0.72	0.07	0.05	0.08	0.05	0.70
Heckman, γ_x=1, ρ=0.3	MI MAR	0.21	0.06	0.22	0.06	0.04	-0.06	0.05	0.08	0.05	0.75	0.08	0.05	0.10	0.05	0.62
Heckman, γ_x=1, ρ=0.3	ML HE X excl.	-0.01	0.10	0.10	0.10	0.95	-0.09	0.05	0.11	0.05	0.55	-0.00	0.06	0.06	0.06	0.95
Heckman, γ_x=1, ρ=0.3	ML HE X fully obs.	-0.00	0.09	0.09	0.09	0.94	0.00	0.06	0.06	0.06	0.94	-0.00	0.06	0.06	0.06	0.95
Heckman, γ_x=1, ρ=0.3	UI X excl.	-0.02	0.05	0.06	0.06	0.95	-0.09	0.05	0.11	0.05	0.57	-0.01	0.05	0.05	0.05	0.96
Heckman, γ_x=1, ρ=0.3	UI X fully obs.	-0.00	0.05	0.05	0.06	0.96	0.00	0.05	0.05	0.05	0.96	-0.00	0.05	0.05	0.05	0.95
Heckman, γ_x=1, ρ=0.3	MI HE	-0.02	0.11	0.11	0.10	0.92	-0.06	0.05	0.08	0.05	0.76	-0.01	0.06	0.06	0.06	0.93
Heckman, γ_x=1, ρ=0.3	MI HE X fully obs.	-0.00	0.09	0.09	0.09	0.92	0.00	0.06	0.06	0.06	0.94	-0.00	0.06	0.06	0.06	0.94
Heckman, γ_x=1, ρ=0.3	Stacking SP	-0.01	0.06	0.06	0.07	0.97	-0.05	0.05	0.07	0.06	0.89	-0.01	0.05	0.05	0.06	0.96
Heckman, γ_x=1, ρ=0.3	Stacking full data	0.01	0.05	0.05	0.08	1.00	-0.04	0.05	0.06	0.08	0.98	0.00	0.05	0.05	0.07	0.99
Heckman, γ_x=1, ρ=0.3	Stacking X fully obs.	-0.03	0.05	0.06	0.07	0.97	0.01	0.05	0.05	0.06	0.96	-0.01	0.05	0.05	0.06	0.96
Heckman, γ_x=1, ρ=0.3	NARFCS Shift SP	0.28	0.08	0.29	0.06	0.01	-0.01	0.05	0.05	0.05	0.94	0.11	0.06	0.12	0.05	0.45
Heckman, γ_x=1, ρ=0.3	NARFCS Shift full data	-0.00	0.04	0.04	0.06	0.99	0.00	0.05	0.05	0.05	0.94	-0.00	0.05	0.05	0.05	0.95
Heckman, γ_x=1, ρ=0.3	NARFCS Int. full data	0.00	0.03	0.03	0.06	1.00	0.00	0.04	0.04	0.05	0.99	0.00	0.03	0.03	0.05	1.00
Heckman, γ_x=1, ρ=0.3	NARFCS Int. X fully obs.	-0.00	0.03	0.03	0.06	1.00	0.00	0.03	0.03	0.05	1.00	0.00	0.03	0.03	0.05	1.00
Heckman, γ_x=1, ρ=0.3	MI RI	0.15	0.09	0.17	0.24	1.00	-0.08	0.05	0.09	0.07	0.85	0.06	0.06	0.08	0.11	0.99
Heckman, γ_x=1, ρ=0.3	MI RI X fully obs.	0.33	0.12	0.35	0.25	0.83	-0.13	0.06	0.14	0.11	0.86	0.13	0.07	0.15	0.11	0.84
Heckman, $\gamma_x = -1$, ρ=0.3	All cases	-0.00	0.03	0.03	0.03	0.96	0.00	0.03	0.03	0.03	0.95	0.00	0.03	0.03	0.03	0.94
Heckman, $\gamma_x = -1$, ρ=0.3	CC	0.17	0.05	0.18	0.05	0.09	0.07	0.05	0.09	0.05	0.72	0.07	0.05	0.09	0.05	0.69
Heckman, $\gamma_x = -1$, ρ=0.3	MI MAR	0.21	0.06	0.22	0.06	0.05	0.07	0.05	0.08	0.05	0.76	0.09	0.05	0.10	0.05	0.58
Heckman, $\gamma_x = -1$, ρ=0.3	ML HE X excl.	0.00	0.10	0.10	0.10	0.95	0.10	0.05	0.11	0.05	0.54	0.00	0.06	0.06	0.06	0.96
Heckman, $\gamma_x = -1$, ρ=0.3	ML HE X fully obs.	-0.00	0.09	0.09	0.09	0.96	0.00	0.06	0.06	0.06	0.95	0.00	0.06	0.06	0.06	0.96
Heckman, $\gamma_x = -1$, ρ=0.3	UI X excl.	-0.02	0.05	0.05	0.06	0.96	0.10	0.05	0.11	0.05	0.53	-0.00	0.05	0.05	0.05	0.96
Heckman, $\gamma_x = -1$, ρ=0.3	UI X fully obs.	-0.00	0.05	0.05	0.06	0.96	-0.00	0.05	0.05	0.05	0.95	0.00	0.05	0.05	0.05	0.96
Heckman, $\gamma_x = -1$, ρ=0.3	MI HE	0.02	0.09	0.09	0.09	0.91	0.12	0.06	0.14	0.06	0.40	0.01	0.06	0.06	0.06	0.95
Heckman, $\gamma_x = -1$, ρ=0.3	MI HE X fully obs.	-0.00	0.09	0.09	0.09	0.94	-0.00	0.06	0.06	0.06	0.96	0.00	0.06	0.06	0.06	0.96
Heckman, $\gamma_x = -1$, ρ=0.3	Stacking SP	0.03	0.06	0.07	0.06	0.92	0.12	0.05	0.14	0.06	0.50	0.01	0.05	0.05	0.06	0.96
Heckman, $\gamma_x = -1$, ρ=0.3	Stacking full data	0.01	0.05	0.05	0.08	0.99	0.04	0.05	0.07	0.08	0.98	0.01	0.05	0.05	0.07	0.98
Heckman, $\gamma_x = -1$, ρ=0.3	Stacking X fully obs.	-0.03	0.05	0.06	0.07	0.98	-0.01	0.05	0.05	0.06	0.96	-0.01	0.05	0.05	0.06	0.96
Heckman, $\gamma_x = -1$, ρ=0.3	NARFCS Shift SP	0.28	0.08	0.29	0.06	0.02	0.02	0.05	0.05	0.05	0.94	0.11	0.05	0.13	0.05	0.40
Heckman, $\gamma_x = -1$, ρ=0.3	NARFCS Shift full data	-0.00	0.04	0.04	0.06	0.99	0.00	0.05	0.05	0.05	0.95	0.00	0.05	0.05	0.05	0.96
Heckman, $\gamma_x = -1$, ρ=0.3	NARFCS Int. full data	-0.00	0.03	0.03	0.06	1.00	0.00	0.04	0.04	0.05	0.99	0.00	0.03	0.03	0.05	0.99
Heckman, $\gamma_x = -1$, ρ=0.3	NARFCS Int. X fully obs.	-0.00	0.03	0.03	0.06	1.00	0.00	0.03	0.03	0.05	0.99	0.00	0.03	0.03	0.05	1.00
Heckman, $\gamma_x = -1$, ρ=0.3	MI RI	0.19	0.08	0.21	0.23	0.99	0.07	0.05	0.08	0.07	0.91	0.08	0.05	0.10	0.10	0.98
Heckman, $\gamma_x = -1$, ρ=0.3	MI RI X fully obs.	0.24	0.12	0.27	0.25	0.94	0.10	0.07	0.12	0.11	0.93	0.10	0.06	0.12	0.11	0.95

Table S3: (1)Performance of the methods in the simulation study 1 with simultaneous monotone missing. DGM: data-generating mechanism; empse: empirical standard error; rmse: root mean square error; modse: model standard error; cov: empirical coverage of the 95% confidence intervals; see the description of abbreviations in Table S2.

		Intercept					β_x					β_z				
DGM	Method	bias	empse	rmse	modse	cov	bias	empse	rmse	modse	cov	bias	empse	rmse	modse	cov
Heckman, γ_x=1, ρ=0.6	All cases	-0.00	0.03	0.03	0.03	0.95	-0.00	0.03	0.03	0.03	0.95	0.00	0.03	0.03	0.03	0.95
Heckman, γ_x=1, ρ=0.6	CC	0.35	0.05	0.35	0.05	0.00	-0.14	0.05	0.15	0.05	0.17	0.14	0.05	0.15	0.05	0.17
Heckman, γ_x=1, ρ=0.6	MI MAR	0.42	0.05	0.43	0.06	0.00	-0.13	0.05	0.14	0.05	0.24	0.17	0.05	0.18	0.05	0.08
Heckman, γ_x=1, ρ=0.6	ML HE X excl.	0.00	0.09	0.09	0.09	0.94	-0.20	0.05	0.21	0.05	0.02	-0.00	0.06	0.06	0.06	0.95
Heckman, γ_x=1, ρ=0.6	ML HE X fully obs.	0.00	0.08	0.08	0.08	0.95	-0.00	0.06	0.06	0.05	0.94	-0.00	0.05	0.05	0.05	0.95
Heckman, γ_x=1, ρ=0.6	UI X excl.	0.17	0.05	0.17	0.05	0.13	-0.17	0.05	0.17	0.05	0.07	0.07	0.05	0.08	0.05	0.73
Heckman, γ_x=1, ρ=0.6	UI X fully obs.	0.18	0.05	0.19	0.05	0.07	-0.07	0.05	0.09	0.05	0.68	0.07	0.05	0.09	0.05	0.69
Heckman, γ_x=1, ρ=0.6	MI HE	-0.04	0.10	0.11	0.09	0.90	-0.11	0.06	0.13	0.06	0.51	-0.02	0.06	0.06	0.06	0.93
Heckman, γ_x=1, ρ=0.6	MI HE X fully obs.	0.00	0.08	0.08	0.08	0.94	-0.00	0.06	0.06	0.06	0.95	-0.00	0.06	0.05	0.06	0.95
Heckman, γ_x=1, ρ=0.6	Stacking SP	0.23	0.06	0.24	0.07	0.05	-0.14	0.05	0.14	0.06	0.33	0.09	0.05	0.10	0.05	0.62
Heckman, γ_x=1, ρ=0.6	Stacking full data	0.04	0.05	0.06	0.09	0.98	-0.10	0.05	0.11	0.08	0.87	0.01	0.05	0.05	0.08	0.99
Heckman, γ_x=1, ρ=0.6	Stacking X fully obs.	-0.06	0.06	0.08	0.08	0.94	0.03	0.05	0.06	0.07	0.97	-0.03	0.05	0.06	0.07	0.97
Heckman, γ_x=1, ρ=0.6	NARFCS Shift SP	0.55	0.07	0.56	0.06	0.00	-0.04	0.05	0.06	0.05	0.88	0.22	0.06	0.23	0.05	0.02
Heckman, γ_x=1, ρ=0.6	NARFCS Shift full data	0.00	0.04	0.04	0.06	0.99	-0.00	0.05	0.05	0.05	0.93	-0.00	0.05	0.05	0.05	0.95
Heckman, γ_x=1, ρ=0.6	NARFCS Int. full data	0.00	0.03	0.03	0.06	1.00	-0.00	0.04	0.04	0.05	0.99	0.00	0.03	0.03	0.05	0.99
Heckman, γ_x=1, ρ=0.6	NARFCS Int. X fully obs.	-0.00	0.03	0.03	0.06	1.00	-0.00	0.03	0.03	0.05	1.00	0.00	0.03	0.03	0.05	1.00
Heckman, γ_x=1, ρ=0.6	MI RI	0.39	0.08	0.40	0.22	0.59	-0.15	0.05	0.16	0.07	0.41	0.16	0.06	0.17	0.10	0.70
Heckman, γ_x=1, ρ=0.6	MI RI X fully obs.	0.65	0.10	0.66	0.22	0.04	-0.26	0.06	0.27	0.10	0.12	0.26	0.06	0.27	0.10	0.14
Heckman, $\gamma_x = -1$, ρ=0.6	All cases	0.00	0.03	0.03	0.03	0.95	0.00	0.03	0.03	0.03	0.94	-0.00	0.03	0.03	0.03	0.96
Heckman, $\gamma_x = -1$, ρ=0.6	CC	0.35	0.05	0.36	0.05	0.00	0.14	0.05	0.15	0.05	0.16	0.14	0.05	0.15	0.05	0.15
Heckman, $\gamma_x = -1$, ρ=0.6	MI MAR	0.43	0.06	0.43	0.06	0.00	0.13	0.05	0.14	0.05	0.22	0.17	0.05	0.18	0.05	0.06
Heckman, $\gamma_x = -1$, ρ=0.6	ML HE X excl.	0.00	0.10	0.10	0.09	0.94	0.20	0.05	0.21	0.05	0.02	-0.00	0.06	0.06	0.06	0.95
Heckman, $\gamma_x = -1$, ρ=0.6	ML HE X fully obs.	-0.00	0.08	0.08	0.08	0.92	0.00	0.05	0.05	0.05	0.95	-0.00	0.05	0.05	0.05	0.95
Heckman, $\gamma_x = -1$, ρ=0.6	UI X excl.	0.17	0.05	0.18	0.05	0.12	0.17	0.05	0.17	0.05	0.07	0.07	0.05	0.08	0.05	0.75
Heckman, $\gamma_x = -1$, ρ=0.6	UI X fully obs.	0.18	0.05	0.19	0.05	0.07	0.07	0.05	0.09	0.05	0.67	0.07	0.05	0.09	0.05	0.69
Heckman, $\gamma_x = -1$, ρ=0.6	MI HE	0.11	0.07	0.13	0.07	0.66	0.30	0.07	0.30	0.06	0.01	0.04	0.05	0.07	0.05	0.87
Heckman, $\gamma_x = -1$, ρ=0.6	MI HE X fully obs.	-0.00	0.08	0.08	0.08	0.93	-0.00	0.06	0.06	0.06	0.95	-0.00	0.05	0.05	0.06	0.95
Heckman, $\gamma_x = -1$, ρ=0.6	Stacking SP	0.25	0.05	0.25	0.06	0.01	0.18	0.05	0.19	0.06	0.11	0.10	0.05	0.11	0.06	0.58
Heckman, $\gamma_x = -1$, ρ=0.6	Stacking full data	0.04	0.05	0.07	0.09	0.98	0.10	0.05	0.11	0.08	0.86	0.01	0.05	0.05	0.08	0.99
Heckman, $\gamma_x = -1$, ρ=0.6	Stacking X fully obs.	-0.06	0.06	0.08	0.08	0.95	-0.02	0.05	0.06	0.07	0.97	-0.03	0.05	0.06	0.07	0.97
Heckman, $\gamma_x = -1$, ρ=0.6	NARFCS Shift SP	0.56	0.08	0.56	0.06	0.00	0.04	0.05	0.06	0.05	0.89	0.22	0.05	0.23	0.05	0.01
Heckman, $\gamma_x = -1$, ρ=0.6	NARFCS Shift full data	0.00	0.04	0.04	0.06	0.99	0.00	0.05	0.05	0.05	0.95	-0.00	0.05	0.05	0.05	0.96
Heckman, $\gamma_x = -1$, ρ=0.6	NARFCS Int. full data	-0.00	0.03	0.03	0.06	1.00	0.00	0.04	0.04	0.05	0.98	-0.00	0.03	0.03	0.05	1.00
Heckman, $\gamma_x = -1$, ρ=0.6	NARFCS Int. X fully obs.	0.00	0.03	0.03	0.06	1.00	-0.00	0.03	0.03	0.05	0.99	-0.00	0.03	0.03	0.05	1.00
Heckman, $\gamma_x = -1$, ρ=0.6	MI RI	0.43	0.08	0.43	0.20	0.38	0.13	0.05	0.14	0.07	0.52	0.17	0.05	0.18	0.09	0.56
Heckman, $\gamma_x = -1$, ρ=0.6	MI RI X fully obs.	0.59	0.10	0.60	0.21	0.09	0.24	0.06	0.25	0.10	0.20	0.23	0.06	0.24	0.10	0.21
Logistic, γ_x=0.5, γ_y=0	All cases	0.00	0.03	0.03	0.03	0.94	0.00	0.03	0.03	0.03	0.94	0.00	0.03	0.03	0.03	0.94
Logistic, γ_x=0.5, γ_y=0	CC	0.00	0.05	0.05	0.05	0.94	0.00	0.05	0.05	0.05	0.95	-0.00	0.05	0.05	0.05	0.95
Logistic, γ_x=0.5, γ_y=0	MI MAR	0.00	0.06	0.05	0.05	0.93	0.00	0.05	0.05	0.05	0.93	-0.00	0.05	0.05	0.05	0.95
Logistic, γ_x=0.5, γ_y=0	ML HE X excl.	-0.00	0.12	0.12	0.12	0.96	0.00	0.05	0.05	0.05	0.94	-0.00	0.06	0.06	0.06	0.95
Logistic, γ_x=0.5, γ_y=0	ML HE X fully obs.	-0.00	0.12	0.12	0.12	0.96	0.00	0.05	0.05	0.05	0.95	-0.00	0.06	0.06	0.06	0.95
Logistic, γ_x=0.5, γ_y=0	UI X excl.	-0.22	0.05	0.22	0.05	0.01	-0.00	0.05	0.05	0.05	0.95	-0.08	0.05	0.09	0.05	0.67
Logistic, γ_x=0.5, γ_y=0	UI X fully obs.	-0.22	0.05	0.22	0.05	0.01	0.04	0.05	0.06	0.05	0.85	-0.08	0.05	0.09	0.05	0.67
Logistic, γ_x=0.5, γ_y=0	MI HE	-0.00	0.12	0.12	0.11	0.90	0.01	0.05	0.05	0.05	0.93	-0.00	0.06	0.06	0.06	0.94
Logistic, γ_x=0.5, γ_y=0	MI HE X fully obs.	-0.00	0.12	0.12	0.12	0.94	0.00	0.05	0.05	0.05	0.95	-0.00	0.06	0.06	0.06	0.95
Logistic, γ_x=0.5, γ_y=0	Stacking SP	-0.22	0.06	0.23	0.06	0.03	0.05	0.05	0.07	0.06	0.91	-0.08	0.05	0.10	0.06	0.71
Logistic, γ_x=0.5, γ_y=0	Stacking full data	0.00	0.04	0.04	0.06	0.99	0.00	0.05	0.05	0.05	0.96	-0.00	0.05	0.05	0.05	0.96
Logistic, γ_x=0.5, γ_y=0	Stacking X fully obs.	0.00	0.04	0.04	0.05	0.99	0.00	0.05	0.05	0.05	0.94	-0.00	0.05	0.05	0.05	0.96
Logistic, γ_x=0.5, γ_y=0	NARFCS Shift SP	-0.00	0.08	0.08	0.05	0.80	0.00	0.05	0.05	0.05	0.94	-0.00	0.05	0.05	0.05	0.92
Logistic, γ_x=0.5, γ_y=0	NARFCS Shift full data	0.00	0.04	0.04	0.05	0.99	0.00	0.05	0.05	0.05	0.94	-0.00	0.05	0.05	0.05	0.96
Logistic, γ_x=0.5, γ_y=0	NARFCS Int. full data	0.00	0.03	0.03	0.05	1.00	0.00	0.04	0.04	0.05	0.98	0.00	0.03	0.03	0.05	0.99
Logistic, γ_x=0.5, γ_y=0	NARFCS Int. X fully obs.	0.00	0.03	0.03	0.05	1.00	0.00	0.03	0.03	0.05	0.99	0.00	0.03	0.03	0.05	0.99
Logistic, γ_x=0.5, γ_y=0	MI RI	-0.06	0.08	0.10	0.24	1.00	-0.00	0.05	0.05	0.07	0.98	-0.02	0.05	0.06	0.10	1.00
Logistic, γ_x=0.5, γ_y=0	MI RI X fully obs.	-0.03	0.08	0.09	0.25	1.00	0.01	0.05	0.05	0.06	0.99	-0.01	0.05	0.05	0.10	1.00
Logistic, $\gamma_x = -0.5$, γ_y=0	All cases	-0.00	0.03	0.03	0.03	0.95	0.00	0.03	0.03	0.03	0.95	0.00	0.03	0.03	0.03	0.95
Logistic, $\gamma_x = -0.5$, γ_y=0	CC	-0.00	0.05	0.05	0.05	0.94	0.00	0.05	0.05	0.05	0.95	0.00	0.05	0.05	0.05	0.96
Logistic, $\gamma_x = -0.5$, γ_y=0	MI MAR	-0.00	0.05	0.05	0.05	0.95	0.00	0.05	0.05	0.05	0.95	0.00	0.05	0.05	0.05	0.94
Logistic, $\gamma_x = -0.5$, γ_y=0	ML HE X excl.	0.00	0.13	0.13	0.12	0.94	0.00	0.05	0.05	0.05	0.95	0.00	0.06	0.06	0.06	0.95
Logistic, $\gamma_x = -0.5$, γ_y=0	ML HE X fully obs.	0.00	0.12	0.12	0.12	0.94	0.00	0.05	0.05	0.05	0.95	0.00	0.06	0.06	0.06	0.95
Logistic, $\gamma_x = -0.5$, γ_y=0	UI X excl.	-0.22	0.05	0.22	0.05	0.01	0.01	0.05	0.05	0.05	0.95	-0.08	0.05	0.09	0.05	0.70
Logistic, $\gamma_x = -0.5$, γ_y=0	UI X fully obs.	-0.22	0.05	0.22	0.05	0.01	-0.04	0.05	0.06	0.05	0.89	-0.08	0.05	0.09	0.05	0.69
Logistic, $\gamma_x = -0.5$, γ_y=0	MI HE	0.00	0.12	0.12	0.11	0.89	0.01	0.05	0.05	0.05	0.93	0.00	0.06	0.06	0.06	0.94
Logistic, $\gamma_x = -0.5$, γ_y=0	MI HE X fully obs.	0.00	0.12	0.12	0.12	0.93	0.00	0.05	0.05	0.05	0.95	0.00	0.06	0.06	0.06	0.94
Logistic, $\gamma_x = -0.5$, γ_y=0	Stacking SP	-0.20	0.05	0.21	0.06	0.04	0.06	0.05	0.08	0.06	0.88	-0.07	0.05	0.09	0.06	0.79
Logistic, $\gamma_x = -0.5$, γ_y=0	Stacking full data	-0.00	0.04	0.04	0.06	0.99	0.00	0.05	0.05	0.05	0.96	0.00	0.05	0.05	0.05	0.96
Logistic, $\gamma_x = -0.5$, γ_y=0	Stacking X fully obs.	-0.00	0.04	0.04	0.05	0.99	0.00	0.05	0.05	0.05	0.95	0.00	0.05	0.05	0.05	0.96
Logistic, $\gamma_x = -0.5$, γ_y=0	NARFCS Shift SP	-0.00	0.08	0.08	0.05	0.81	0.00	0.05	0.05	0.05	0.95	0.00	0.05	0.05	0.05	0.94
Logistic, $\gamma_x = -0.5$, γ_y=0	NARFCS Shift full data	-0.00	0.04	0.04	0.05	0.99	0.00	0.05	0.05	0.05	0.95	0.00	0.05	0.05	0.05	0.96
Logistic, $\gamma_x = -0.5$, γ_y=0	NARFCS Int. full data	-0.00	0.03	0.03	0.05	0.99	0.00	0.04	0.04	0.05	0.98	0.00	0.03	0.03	0.05	0.99
Logistic, $\gamma_x = -0.5$, γ_y=0	NARFCS Int. X fully obs.	-0.00	0.03	0.03	0.05	1.00	0.00	0.03	0.03	0.05	0.99	0.00	0.03	0.03	0.05	0.99
Logistic, $\gamma_x = -0.5$, γ_y=0	MI RI	-0.05	0.08	0.09	0.24	1.00	-0.00	0.05	0.05	0.07	0.99	-0.02	0.05	0.05	0.10	1.00
Logistic, $\gamma_x = -0.5$, γ_y=0	MI RI X fully obs.	-0.09	0.08	0.12	0.25	1.00	-0.01	0.05	0.05	0.06	0.99	-0.03	0.05	0.06	0.10	0.99

Table S4: (2)Performance of the methods in the simulation study 1 with simultaneous monotone missing. DGM: data-generating mechanism; empse: empirical standard error; rmse: root mean square error; modse: model standard error; cov: empirical coverage of the 95% confidence intervals; see the description of abbreviations in Table S2.

		Intercept					β_x					β_z				
DGM	Method	bias	empse	rmse	modse	cov	bias	empse	rmse	modse	cov	bias	empse	rmse	modse	cov
Logistic, γ_x=0, γ_y=0.5	All cases	-0.00	0.03	0.03	0.03	0.96	-0.00	0.03	0.03	0.03	0.95	0.00	0.03	0.03	0.03	0.95
Logistic, γ_x=0, γ_y=0.5	CC	0.20	0.04	0.21	0.05	0.01	-0.02	0.05	0.05	0.04	0.93	0.06	0.04	0.07	0.05	0.75
Logistic, γ_x=0, γ_y=0.5	MI MAR	0.24	0.05	0.24	0.05	0.00	-0.02	0.05	0.05	0.05	0.92	0.07	0.05	0.08	0.05	0.69
Logistic, γ_x=0, γ_y=0.5	ML HE X excl.	-0.00	0.11	0.11	0.11	0.96	-0.02	0.05	0.05	0.04	0.91	0.00	0.05	0.05	0.05	0.95
Logistic, γ_x=0, γ_y=0.5	ML HE X fully obs.	-0.00	0.11	0.11	0.11	0.96	-0.00	0.05	0.05	0.05	0.95	0.00	0.05	0.05	0.05	0.95
Logistic, γ_x=0, γ_y=0.5	UI X excl.	-0.01	0.05	0.05	0.05	0.95	-0.02	0.05	0.05	0.05	0.93	0.00	0.05	0.05	0.05	0.97
Logistic, γ_x=0, γ_y=0.5	UI X fully obs.	-0.01	0.05	0.05	0.05	0.95	0.00	0.05	0.05	0.05	0.96	0.00	0.05	0.05	0.05	0.97
Logistic, γ_x=0, γ_y=0.5	MI HE	0.00	0.11	0.11	0.10	0.91	0.01	0.05	0.06	0.05	0.93	0.00	0.05	0.06	0.05	0.94
Logistic, γ_x=0, γ_y=0.5	MI HE X fully obs.	0.00	0.11	0.11	0.12	0.94	-0.00	0.05	0.05	0.05	0.95	0.00	0.06	0.06	0.06	0.95
Logistic, γ_x=0, γ_y=0.5	Stacking SP	0.03	0.05	0.06	0.06	0.93	0.02	0.05	0.05	0.06	0.97	0.01	0.05	0.05	0.05	0.97
Logistic, γ_x=0, γ_y=0.5	Stacking full data	0.01	0.04	0.04	0.06	0.99	-0.01	0.05	0.05	0.05	0.97	0.00	0.04	0.05	0.05	0.98
Logistic, γ_x=0, γ_y=0.5	Stacking X fully obs.	0.01	0.04	0.04	0.06	0.99	-0.00	0.05	0.05	0.05	0.97	0.00	0.04	0.04	0.05	0.97
Logistic, γ_x=0, γ_y=0.5	NARFCS Shift SP	0.35	0.07	0.36	0.05	0.00	-0.01	0.05	0.05	0.05	0.95	0.10	0.05	0.11	0.05	0.44
Logistic, γ_x=0, γ_y=0.5	NARFCS Shift full data	0.00	0.04	0.04	0.05	0.99	-0.00	0.05	0.05	0.05	0.95	0.00	0.04	0.04	0.05	0.96
Logistic, γ_x=0, γ_y=0.5	NARFCS Int. full data	0.00	0.03	0.03	0.05	0.99	0.00	0.04	0.04	0.05	0.98	0.00	0.03	0.03	0.05	0.99
Logistic, γ_x=0, γ_y=0.5	NARFCS Int. X fully obs.	-0.00	0.03	0.03	0.05	0.99	-0.00	0.03	0.03	0.05	0.99	0.00	0.03	0.03	0.05	0.99
Logistic, γ_x=0, γ_y=0.5	MI RI	0.18	0.07	0.20	0.24	0.99	-0.03	0.05	0.05	0.06	0.98	0.05	0.05	0.07	0.08	0.98
Logistic, γ_x=0, γ_y=0.5	MI RI X fully obs.	0.20	0.07	0.21	0.24	1.00	-0.02	0.05	0.05	0.05	0.95	0.06	0.05	0.07	0.08	0.97
Logistic, γ_x=0, $\gamma_y=-0.5$	All cases	-0.00	0.03	0.03	0.03	0.94	-0.00	0.03	0.03	0.03	0.94	0.00	0.03	0.03	0.03	0.96
Logistic, γ_x=0, $\gamma_y=-0.5$	CC	-0.20	0.05	0.21	0.05	0.02	-0.02	0.05	0.05	0.04	0.92	-0.08	0.05	0.10	0.05	0.59
Logistic, γ_x=0, $\gamma_y=-0.5$	MI MAR	-0.23	0.05	0.24	0.05	0.01	-0.02	0.05	0.05	0.05	0.92	-0.10	0.05	0.11	0.05	0.50
Logistic, γ_x=0, $\gamma_y=-0.5$	ML HE X excl.	-0.01	0.12	0.12	0.12	0.95	-0.02	0.05	0.05	0.04	0.91	-0.00	0.07	0.07	0.07	0.95
Logistic, γ_x=0, $\gamma_y=-0.5$	ML HE X fully obs.	-0.01	0.12	0.12	0.12	0.95	-0.00	0.05	0.05	0.05	0.94	-0.00	0.07	0.07	0.07	0.95
Logistic, γ_x=0, $\gamma_y=-0.5$	UI X excl.	-0.42	0.05	0.42	0.05	0.00	-0.02	0.05	0.05	0.05	0.94	-0.17	0.05	0.18	0.05	0.07
Logistic, γ_x=0, $\gamma_y=-0.5$	UI X fully obs.	-0.42	0.05	0.42	0.05	0.00	-0.04	0.05	0.06	0.05	0.86	-0.17	0.05	0.18	0.05	0.07
Logistic, γ_x=0, $\gamma_y=-0.5$	MI HE	-0.01	0.12	0.12	0.11	0.90	0.01	0.05	0.05	0.05	0.93	-0.00	0.07	0.07	0.06	0.93
Logistic, γ_x=0, $\gamma_y=-0.5$	MI HE X fully obs.	-0.01	0.12	0.12	0.12	0.94	-0.00	0.05	0.05	0.05	0.94	-0.00	0.07	0.07	0.07	0.94
Logistic, γ_x=0, $\gamma_y=-0.5$	Stacking SP	-0.43	0.05	0.43	0.06	0.00	0.03	0.05	0.06	0.06	0.95	-0.18	0.05	0.19	0.06	0.10
Logistic, γ_x=0, $\gamma_y=-0.5$	Stacking full data	-0.01	0.04	0.04	0.06	0.99	-0.01	0.05	0.05	0.05	0.96	-0.00	0.05	0.05	0.05	0.96
Logistic, γ_x=0, $\gamma_y=-0.5$	Stacking X fully obs.	-0.00	0.04	0.04	0.06	0.99	-0.00	0.05	0.05	0.05	0.96	-0.00	0.05	0.05	0.05	0.96
Logistic, γ_x=0, $\gamma_y=-0.5$	NARFCS Shift SP	-0.35	0.08	0.36	0.05	0.00	-0.01	0.05	0.05	0.05	0.94	-0.14	0.06	0.15	0.05	0.21
Logistic, γ_x=0, $\gamma_y=-0.5$	NARFCS Shift full data	-0.00	0.04	0.04	0.05	0.99	-0.00	0.05	0.05	0.05	0.94	0.00	0.05	0.05	0.05	0.95
Logistic, γ_x=0, $\gamma_y=-0.5$	NARFCS Int. full data	-0.00	0.03	0.03	0.05	1.00	-0.00	0.04	0.04	0.05	0.97	0.00	0.03	0.03	0.05	1.00
Logistic, γ_x=0, $\gamma_y=-0.5$	NARFCS Int. X fully obs.	-0.00	0.03	0.03	0.05	1.00	-0.00	0.03	0.03	0.05	0.99	0.00	0.03	0.03	0.05	1.00
Logistic, γ_x=0, $\gamma_y=-0.5$	MI RI	-0.29	0.08	0.30	0.24	0.96	-0.03	0.05	0.05	0.06	0.98	-0.12	0.05	0.13	0.11	0.93
Logistic, γ_x=0, $\gamma_y=-0.5$	MI RI X fully obs.	-0.32	0.08	0.33	0.24	0.92	-0.03	0.05	0.06	0.05	0.92	-0.13	0.05	0.14	0.11	0.91
Logistic, γ_x=0.5, γ_y=0.5	All cases	-0.00	0.03	0.03	0.03	0.95	0.00	0.03	0.03	0.03	0.95	-0.00	0.03	0.03	0.03	0.94
Logistic, γ_x=0.5, γ_y=0.5	CC	0.20	0.05	0.21	0.05	0.01	-0.05	0.05	0.07	0.05	0.83	0.05	0.05	0.07	0.05	0.79
Logistic, γ_x=0.5, γ_y=0.5	MI MAR	0.23	0.05	0.24	0.05	0.01	-0.05	0.05	0.07	0.05	0.82	0.06	0.05	0.08	0.05	0.75
Logistic, γ_x=0.5, γ_y=0.5	ML HE X excl.	0.00	0.12	0.12	0.12	0.95	-0.06	0.05	0.08	0.05	0.75	-0.00	0.05	0.05	0.06	0.95
Logistic, γ_x=0.5, γ_y=0.5	ML HE X fully obs.	0.00	0.12	0.12	0.12	0.94	0.00	0.05	0.05	0.05	0.95	-0.00	0.05	0.05	0.05	0.95
Logistic, γ_x=0.5, γ_y=0.5	UI X excl.	-0.01	0.05	0.05	0.05	0.94	-0.06	0.05	0.08	0.05	0.79	-0.00	0.05	0.05	0.05	0.96
Logistic, γ_x=0.5, γ_y=0.5	UI X fully obs.	-0.01	0.05	0.05	0.05	0.94	0.01	0.05	0.05	0.05	0.95	-0.00	0.05	0.05	0.05	0.96
Logistic, γ_x=0.5, γ_y=0.5	MI HE	0.00	0.12	0.12	0.11	0.90	-0.03	0.05	0.06	0.05	0.91	-0.00	0.06	0.06	0.05	0.94
Logistic, γ_x=0.5, γ_y=0.5	MI HE X fully obs.	0.01	0.12	0.12	0.12	0.93	0.00	0.06	0.06	0.06	0.95	-0.00	0.06	0.06	0.06	0.95
Logistic, γ_x=0.5, γ_y=0.5	Stacking SP	0.01	0.06	0.06	0.06	0.95	-0.02	0.05	0.05	0.06	0.97	0.00	0.05	0.05	0.05	0.97
Logistic, γ_x=0.5, γ_y=0.5	Stacking full data	0.01	0.04	0.04	0.06	0.99	-0.02	0.05	0.05	0.06	0.98	0.00	0.05	0.05	0.05	0.97
Logistic, γ_x=0.5, γ_y=0.5	Stacking X fully obs.	0.00	0.04	0.04	0.06	0.99	0.00	0.05	0.05	0.05	0.96	-0.00	0.05	0.05	0.05	0.96
Logistic, γ_x=0.5, γ_y=0.5	NARFCS Shift SP	0.33	0.07	0.34	0.05	0.00	-0.02	0.05	0.05	0.05	0.92	0.09	0.05	0.10	0.05	0.55
Logistic, γ_x=0.5, γ_y=0.5	NARFCS Shift full data	-0.00	0.04	0.04	0.05	0.99	0.00	0.05	0.05	0.05	0.95	-0.00	0.05	0.05	0.05	0.96
Logistic, γ_x=0.5, γ_y=0.5	NARFCS Int. full data	0.00	0.03	0.03	0.05	1.00	0.00	0.04	0.04	0.05	0.99	-0.00	0.03	0.03	0.05	0.99
Logistic, γ_x=0.5, γ_y=0.5	NARFCS Int. X fully obs.	0.00	0.03	0.03	0.05	1.00	0.00	0.03	0.03	0.05	0.99	-0.00	0.03	0.03	0.05	0.99
Logistic, γ_x=0.5, γ_y=0.5	MI RI	0.17	0.08	0.19	0.24	1.00	-0.06	0.05	0.08	0.07	0.94	0.04	0.05	0.07	0.08	0.98
Logistic, γ_x=0.5, γ_y=0.5	MI RI X fully obs.	0.23	0.08	0.24	0.24	0.99	-0.06	0.05	0.08	0.08	0.96	0.06	0.05	0.08	0.08	0.96
Logistic, $\gamma_x=-0.5$, $\gamma_y=-0.5$	All cases	-0.00	0.03	0.03	0.03	0.95	0.00	0.03	0.03	0.03	0.95	-0.00	0.03	0.03	0.03	0.94
Logistic, $\gamma_x=-0.5$, $\gamma_y=-0.5$	CC	-0.20	0.05	0.21	0.05	0.02	-0.05	0.05	0.06	0.05	0.84	-0.08	0.05	0.09	0.05	0.62
Logistic, $\gamma_x=-0.5$, $\gamma_y=-0.5$	MI MAR	-0.23	0.05	0.24	0.05	0.01	-0.05	0.05	0.06	0.05	0.84	-0.09	0.05	0.10	0.05	0.54
Logistic, $\gamma_x=-0.5$, $\gamma_y=-0.5$	ML HE X excl.	-0.00	0.13	0.13	0.13	0.96	-0.05	0.05	0.07	0.05	0.78	-0.00	0.07	0.07	0.07	0.94
Logistic, $\gamma_x=-0.5$, $\gamma_y=-0.5$	ML HE X fully obs.	-0.01	0.12	0.12	0.12	0.96	-0.00	0.05	0.05	0.05	0.94	-0.00	0.07	0.07	0.07	0.94
Logistic, $\gamma_x=-0.5$, $\gamma_y=-0.5$	UI X excl.	-0.42	0.05	0.42	0.05	0.00	-0.04	0.05	0.06	0.05	0.89	-0.17	0.05	0.17	0.05	0.09
Logistic, $\gamma_x=-0.5$, $\gamma_y=-0.5$	UI X fully obs.	-0.42	0.05	0.42	0.05	0.00	-0.10	0.05	0.11	0.05	0.46	-0.17	0.05	0.17	0.05	0.09
Logistic, $\gamma_x=-0.5$, $\gamma_y=-0.5$	MI HE	-0.00	0.13	0.13	0.11	0.89	-0.02	0.05	0.06	0.05	0.92	-0.00	0.07	0.07	0.07	0.93
Logistic, $\gamma_x=-0.5$, $\gamma_y=-0.5$	MI HE X fully obs.	-0.01	0.12	0.12	0.12	0.93	-0.00	0.05	0.05	0.05	0.94	-0.01	0.07	0.07	0.07	0.94
Logistic, $\gamma_x=-0.5$, $\gamma_y=-0.5$	Stacking SP	-0.41	0.05	0.42	0.06	0.00	0.01	0.05	0.05	0.06	0.96	-0.17	0.05	0.17	0.06	0.14
Logistic, $\gamma_x=-0.5$, $\gamma_y=-0.5$	Stacking full data	-0.01	0.04	0.04	0.06	0.99	-0.01	0.05	0.05	0.06	0.96	-0.00	0.05	0.05	0.06	0.97
Logistic, $\gamma_x=-0.5$, $\gamma_y=-0.5$	Stacking X fully obs.	-0.00	0.04	0.04	0.06	0.99	0.00	0.05	0.05	0.05	0.96	-0.00	0.05	0.05	0.05	0.97
Logistic, $\gamma_x=-0.5$, $\gamma_y=-0.5$	NARFCS Shift SP	-0.33	0.07	0.34	0.05	0.00	-0.02	0.05	0.05	0.05	0.93	-0.13	0.05	0.14	0.05	0.27
Logistic, $\gamma_x=-0.5$, $\gamma_y=-0.5$	NARFCS Shift full data	-0.00	0.04	0.04	0.05	0.99	0.00	0.05	0.05	0.05	0.93	-0.00	0.05	0.05	0.05	0.95
Logistic, $\gamma_x=-0.5$, $\gamma_y=-0.5$	NARFCS Int. full data	-0.00	0.03	0.03	0.05	1.00	0.00	0.04	0.04	0.05	0.98	-0.00	0.03	0.03	0.05	1.00
Logistic, $\gamma_x=-0.5$, $\gamma_y=-0.5$	NARFCS Int. X fully obs.	-0.00	0.03	0.03	0.05	1.00	0.00	0.03	0.03	0.05	0.99	-0.00	0.03	0.03	0.05	1.00
Logistic, $\gamma_x=-0.5$, $\gamma_y=-0.5$	MI RI	-0.29	0.08	0.30	0.24	0.95	-0.05	0.05	0.07	0.07	0.94	-0.11	0.05	0.13	0.11	0.94
Logistic, $\gamma_x=-0.5$, $\gamma_y=-0.5$	MI RI X fully obs.	-0.35	0.08	0.36	0.25	0.88	-0.08	0.05	0.09	0.08	0.88	-0.14	0.05	0.15	0.11	0.88

Table S5: (3)Performance of the methods in the simulation study 1 with simultaneous monotone missing. DGM: data-generating mechanism; empse: empirical standard error; rmse: root mean square error; modse: model standard error; cov: empirical coverage of the 95% confidence intervals; see the description of abbreviations in Table S2.

		Intercept					β_x					β_z				
DGM	Method	bias	empse	rmse	modse	cov	bias	empse	rmse	modse	cov	bias	empse	rmse	modse	cov
$R = I(YX + \varepsilon) > 0$	All cases	0.00	0.03	0.03	0.03	0.95	0.00	0.03	0.03	0.03	0.94	-0.00	0.03	0.03	0.03	0.95
$R = I(YX + \varepsilon) > 0$	CC	0.00	0.03	0.03	0.03	0.95	0.34	0.03	0.34	0.03	0.00	-0.15	0.04	0.15	0.03	0.02
$R = I(YX + \varepsilon) > 0$	MI MAR	0.00	0.03	0.03	0.03	0.95	0.34	0.03	0.34	0.03	0.00	-0.15	0.04	0.15	0.04	0.01
$R = I(YX + \varepsilon) > 0$	ML HE X excl.	-0.04	0.67	0.67	Inf	0.62	0.34	0.04	0.34	Inf	0.07	-0.15	0.07	0.16	Inf	0.16
$R = I(YX + \varepsilon) > 0$	ML HE X fully obs.	-0.03	0.71	0.71	Inf	0.61	0.34	0.05	0.34	Inf	0.08	-0.15	0.06	0.16	Inf	0.17
$R = I(YX + \varepsilon) > 0$	UI X excl.	-0.15	0.03	0.16	0.03	0.01	0.34	0.03	0.34	0.03	0.00	-0.15	0.04	0.15	0.04	0.02
$R = I(YX + \varepsilon) > 0$	UI X fully obs.	-0.15	0.03	0.16	0.03	0.01	0.34	0.03	0.34	0.03	0.00	-0.15	0.04	0.15	0.04	0.02
$R = I(YX + \varepsilon) > 0$	MI HE	0.00	0.14	0.14	0.11	0.75	0.43	0.05	0.43	0.06	0.00	-0.16	0.04	0.17	0.04	0.01
$R = I(YX + \varepsilon) > 0$	MI HE X fully obs.	-0.00	0.28	0.28	0.23	0.58	0.34	0.03	0.34	0.04	0.00	-0.15	0.04	0.15	0.04	0.04
$R = I(YX + \varepsilon) > 0$	Stacking SP	-0.10	0.03	0.11	0.04	0.19	0.38	0.03	0.38	0.04	0.00	-0.16	0.04	0.16	0.04	0.03
$R = I(YX + \varepsilon) > 0$	Stacking full data	0.00	0.03	0.03	0.03	0.97	0.34	0.03	0.34	0.03	0.00	-0.15	0.04	0.15	0.04	0.01
$R = I(YX + \varepsilon) > 0$	Stacking X fully obs.	0.00	0.03	0.03	0.03	0.96	0.34	0.03	0.34	0.03	0.00	-0.15	0.04	0.15	0.04	0.02
$R = I(YX + \varepsilon) > 0$	NARFCS Shift SP	0.00	0.05	0.05	0.03	0.84	0.34	0.03	0.34	0.03	0.00	-0.15	0.04	0.15	0.04	0.01
$R = I(YX + \varepsilon) > 0$	NARFCS Shift full data	0.00	0.03	0.03	0.03	0.96	0.34	0.03	0.34	0.03	0.00	-0.15	0.04	0.15	0.04	0.01
$R = I(YX + \varepsilon) > 0$	NARFCS Int. full data	0.00	0.03	0.03	0.04	0.99	-0.04	0.04	0.06	0.04	0.88	-0.00	0.03	0.03	0.05	0.99
$R = I(YX + \varepsilon) > 0$	NARFCS Int. X fully obs.	0.00	0.03	0.03	0.04	0.98	0.00	0.03	0.03	0.04	0.97	-0.00	0.03	0.03	0.04	0.99
$R = I(YX + \varepsilon) > 0$	MI RI	0.00	0.05	0.05	0.14	1.00	0.33	0.03	0.33	0.04	0.00	-0.14	0.04	0.15	0.04	0.03
$R = I(YX + \varepsilon) > 0$	MI RI X fully obs.	0.00	0.05	0.05	0.14	1.00	0.34	0.03	0.34	0.03	0.00	-0.15	0.04	0.15	0.04	0.02
Pattern-mixture	All cases	0.00	0.05	0.05	0.05	0.96	0.00	0.07	0.07	0.05	0.87	-0.00	0.07	0.07	0.05	0.86
Pattern-mixture	CC	0.00	0.05	0.05	0.04	0.94	1.00	0.05	1.00	0.04	0.00	1.00	0.04	1.00	0.04	0.00
Pattern-mixture	MI MAR	0.00	0.05	0.05	0.05	0.94	1.00	0.05	1.00	0.05	0.00	1.00	0.04	1.00	0.05	0.00
Pattern-mixture	ML HE X excl.	-0.00	1.14	1.14	Inf	0.69	1.00	0.07	1.00	Inf	0.07	1.00	0.09	1.01	Inf	0.07
Pattern-mixture	ML HE X fully obs.	0.05	1.16	1.16	Inf	0.69	1.01	0.09	1.01	Inf	0.07	1.00	0.09	1.01	Inf	0.07
Pattern-mixture	UI X excl.	-0.24	0.05	0.25	0.05	0.00	1.00	0.05	1.00	0.05	0.00	1.00	0.04	1.00	0.05	0.00
Pattern-mixture	UI X fully obs.	-0.24	0.05	0.25	0.05	0.00	1.00	0.05	1.00	0.05	0.00	1.00	0.04	1.00	0.05	0.00
Pattern-mixture	MI HE	0.01	0.19	0.19	0.14	0.69	1.10	0.08	1.11	0.08	0.00	1.00	0.04	1.00	0.05	0.00
Pattern-mixture	MI HE X fully obs.	0.02	0.45	0.45	0.44	0.67	1.00	0.05	1.00	0.05	0.00	1.00	0.05	1.00	0.05	0.00
Pattern-mixture	Stacking SP	-0.17	0.05	0.18	0.05	0.06	1.07	0.05	1.07	0.07	0.00	1.00	0.05	1.00	0.06	0.00
Pattern-mixture	Stacking full data	0.00	0.04	0.04	0.05	0.96	1.00	0.05	1.00	0.05	0.00	1.00	0.04	1.00	0.05	0.00
Pattern-mixture	Stacking X fully obs.	0.00	0.04	0.04	0.05	0.96	1.00	0.05	1.00	0.05	0.00	1.00	0.04	1.00	0.05	0.00
Pattern-mixture	NARFCS Shift SP	0.00	0.07	0.07	0.05	0.81	1.00	0.05	1.00	0.05	0.00	1.00	0.04	1.00	0.05	0.00
Pattern-mixture	NARFCS Shift full data	0.00	0.05	0.05	0.05	0.91	1.00	0.05	1.00	0.05	0.00	1.00	0.04	1.00	0.05	0.00
Pattern-mixture	NARFCS Int. full data	0.00	0.05	0.05	0.07	0.99	0.20	0.09	0.22	0.09	0.39	-0.00	0.07	0.07	0.07	0.95
Pattern-mixture	NARFCS Int. X fully obs.	0.00	0.05	0.05	0.06	0.98	0.00	0.07	0.07	0.06	0.92	-0.00	0.07	0.07	0.06	0.92
Pattern-mixture	MI RI	0.00	0.07	0.07	0.23	1.00	1.00	0.05	1.00	0.06	0.00	1.00	0.04	1.00	0.05	0.00
Pattern-mixture	MI RI X fully obs.	0.00	0.07	0.07	0.22	1.00	1.00	0.05	1.00	0.05	0.00	1.00	0.04	1.00	0.05	0.00

Table S6: (4)Performance of the methods in the simulation study 1 with simultaneous monotone missing. DGM: data-generating mechanism; empse: empirical standard error; rmse: root mean square error; modse: model standard error; cov: empirical coverage of the 95% confidence intervals; see the description of abbreviations in Table S2.

S3 Simulation study 2

S3.1 Pattern-mixture models

For the pattern-mixture models, we assume that the relationship between X and Y depends on whether Y is observed or not. In the first model, referred below as "Pattern-mixture", the response indicator for X does not depend on X:

$$\begin{cases} (X, Z_1, Z_2, \varepsilon_y) \sim N(0, \mathbf{I}) \\ R_x = Bern(0.5) \\ R_y = Bern(0.5) \\ Y = \begin{cases} 1.5X + 1.5Z_1 + \varepsilon_y, \text{if } R_y = 1 \\ -0.5X - 0.5Z_1 + \varepsilon_y, \text{if } R_y = 0. \end{cases} \end{cases}$$

In the second pattern-mixture model, the response indicator for X depends on the value of X:

$$\begin{cases} (X^*, Z_1, Z_2, \varepsilon_y) \sim N(0, \mathbf{I}) \\ R_x = Bern(0.5) \\ R_y = Bern(0.5) \\ X = \begin{cases} 1 + X^*, \text{if } R_x = 1 \\ X^*, \text{if } R_x = 0 \end{cases} \\ Y = \begin{cases} 1.5X + 1.5Z_1 + \varepsilon_y, \text{if } R_y = 1 \\ -0.5X - 0.5Z_1 + \varepsilon_y, \text{if } R_y = 0 \end{cases} \end{cases}$$

Data-generating mechanism	$R_x \sim$	$R_y \sim$	$cor(R_x, X)$	$cor(R_x, Y)$	$cor(R_y, X)$	$cor(R_y, Y)$	$cor(R_x, R_y)$
MAR			0	0	0	0	0.3
Heckman, $R_x \sim X$	X		0.3	0	0	0	0.3
Heckman, $R_x \sim Y$	Y		0	0.3	0	0	0.3
Heckman, $R_x \sim X$ and Y	X and Y		0.3	0.3	0	0	0.3
Heckman, $R_y \sim X$		X	0	0	0.3	0	0.3
Heckman, $R_y \sim Y$		Y	0	0	0	0.3	0.3
Heckman, $R_y \sim X$ and Y		X and Y	0	0	0.3	0.3	0.3
Heckman, $R_x \sim X, R_y \sim X$	X	X	0.3	0	0.3	0	0.3
Heckman, $R_x \sim X, R_y \sim Y$	X	Y	0.3	0	0	0.3	0.3
Heckman, $R_x \sim X, R_y \sim X$ and Y	X	X and Y	0.3	0	0.3	0.3	0.3
Heckman, $R_x \sim Y, R_y \sim X$	Y	X	0	0.3	0.3	0	0.3
Heckman, $R_x \sim Y, R_y \sim Y$	Y	Y	0	0.3	0	0.3	0.3
Heckman, $R_x \sim Y, R_y \sim X$ and Y	Y	X and Y	0	0.3	0.3	0.3	0.3
Heckman, $R_x \sim X$ and $Y, R_y \sim X$	X and Y	X	0.3	0.3	0.3	0	0.3
Heckman, $R_x \sim X$ and $Y, R_y \sim Y$	X and Y	Y	0.3	0.3	0	0.3	0.3
Heckman, $R_x \sim X$ and $Y, R_y \sim X$ and Y	X and Y	X and Y	0.3	0.3	0.3	0.3	0.3
			γ_{xr_x}	γ_{yr_x}	γ_{xr_y}	γ_{yr_y}	
Logistic, R_x=$0.5X$	X		0.5	0	0	0	
Logistic, R_x=$0.5Y$	Y		0	0.5	0	0	
Logistic, R_x=$0.5X$+$0.5Y$	X and Y		0.5	0.5	0	0	
Logistic, R_y=$0.5X$		X	0	0	0.5	0	
Logistic, R_y=$0.5Y$		Y	0	0	0	0.5	
Logistic, R_y=$0.5X$+$0.5Y$		X and Y	0	0	0.5	0.5	
Logistic, R_x=$0.5X, R_y$=$0.5X$	X	X	0.5	0	0.5	0	
Logistic, R_x=$0.5X, R_y$=$0.5Y$	X	Y	0.5	0	0	0.5	
Logistic, R_x=$0.5X, R_y$=$0.5X$+$0.5Y$	X	X and Y	0.5	0	0.5	0.5	
Logistic, R_x=$0.5Y, R_y$=$0.5X$	Y	X	0	0.5	0.5	0	
Logistic, R_x=$0.5Y, R_y$=$0.5Y$	Y	Y	0	0.5	0	0.5	
Logistic, R_x=$0.5Y, R_y$=$0.5X$+$0.5Y$	Y	X and Y	0	0.5	0.5	0.5	
Logistic, R_x=$0.5X$+$0.5Y, R_y$=$0.5X$	X and Y	X	0.5	0.5	0.5	0	
Logistic, R_x=$0.5X$+$0.5Y, R_y$=$0.5Y$	X and Y	Y	0.5	0.5	0	0.5	
Logistic, R_x=$0.5X$+$0.5Y, R_y$=$0.5X$+$0.5Y$	X and Y	X and Y	0.5	0.5	0.5	0.5	

Table S7: Values of the parameters in the considered data-generating mechanisms. Empty cells indicate that the corresponding response indicators do not depend on either X or Y.

S3.2 Simulation study 2 main results

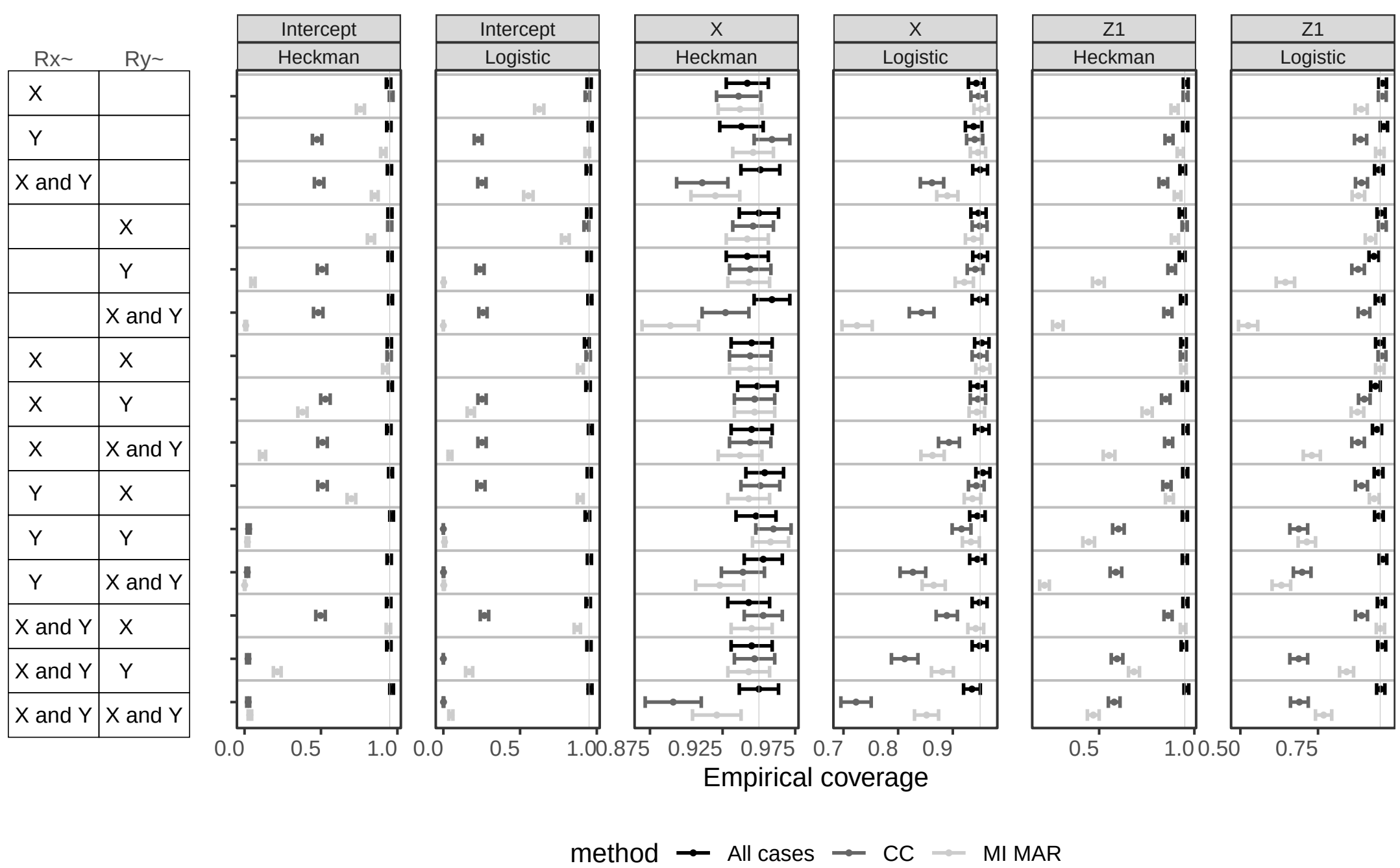

Figure S3: Empirical coverage of 95% confidence intervals in simulation study 2 with non-monotone missing in X and Y for all cases, complete cases (CC), multiple imputation under MAR (MI MAR). The whiskers represent the Monte Carlo standard error of the coverage estimate. The panel to the left describes the relationship between the response indicators and the incomplete variables for each data-generating mechanism. In this panel, Rx$\sim$ indicates that the response indicator for X is related to the variable(s) listed in that row (either X, Y, or both). Similarly, Ry$\sim$ indicates the relationship for the response indicator of Y.

		Intercept					β_x					β_z				
DGM	Method	bias	empse	rmse	modse	cov	bias	empse	rmse	modse	cov	bias	empse	rmse	modse	cov
MAR	All cases	-0.00	0.03	0.03	0.03	0.95	-0.00	0.03	0.03	0.03	0.95	-0.00	0.03	0.03	0.03	0.95
MAR	CC	-0.00	0.06	0.06	0.06	0.94	-0.00	0.05	0.05	0.05	0.95	-0.00	0.06	0.06	0.06	0.96
MAR	MI MAR	-0.00	0.06	0.06	0.06	0.95	-0.00	0.05	0.05	0.05	0.95	-0.00	0.05	0.05	0.06	0.96
MAR	ML HE X cc	-0.00	0.11	0.11	0.11	0.93	-0.00	0.05	0.05	0.05	0.95	-0.00	0.07	0.07	0.07	0.94
MAR	ML HE X excl.	-0.00	0.10	0.10	0.10	0.94	-0.00	0.05	0.05	0.05	0.95	-0.00	0.07	0.07	0.07	0.94
MAR	ML HE X fully obs.	0.00	0.09	0.09	0.09	0.94	-0.00	0.04	0.05	0.04	0.95	-0.00	0.06	0.06	0.06	0.95
MAR	UI X cc	-0.12	0.06	0.13	0.06	0.54	-0.00	0.05	0.05	0.05	0.95	-0.05	0.06	0.08	0.06	0.87
MAR	UI X fully obs.	-0.18	0.05	0.18	0.05	0.07	-0.00	0.05	0.05	0.05	0.96	-0.08	0.05	0.10	0.05	0.65
MAR	MI HE X cc	-0.00	0.11	0.11	0.10	0.92	-0.00	0.05	0.05	0.05	0.95	-0.00	0.07	0.07	0.07	0.94
MAR	MI HE	0.00	0.09	0.09	0.10	0.95	-0.00	0.05	0.05	0.05	0.94	0.00	0.06	0.06	0.07	0.96
MAR	MI HE X fully obs.	0.00	0.09	0.09	0.09	0.93	-0.00	0.05	0.05	0.05	0.94	-0.00	0.06	0.06	0.06	0.93
MAR	Stacking full data	-0.00	0.05	0.05	0.07	0.98	-0.00	0.05	0.05	0.05	0.92	-0.00	0.05	0.05	0.06	0.96
MAR	Stacking X MAR	-0.00	0.05	0.05	0.06	0.98	-0.00	0.05	0.05	0.05	0.95	-0.00	0.05	0.05	0.06	0.96
MAR	Stacking X fully obs.	-0.00	0.04	0.04	0.06	0.99	-0.00	0.05	0.05	0.05	0.95	-0.00	0.05	0.05	0.05	0.97
MAR	NARFCS Shift SP	-0.00	0.11	0.11	0.07	0.79	-0.00	0.05	0.05	0.05	0.94	-0.00	0.07	0.07	0.06	0.91
MAR	NARFCS Shift full data	-0.00	0.05	0.05	0.07	0.99	-0.00	0.05	0.05	0.05	0.94	-0.00	0.05	0.05	0.06	0.96
MAR	NARFCS Int. full data	-0.00	0.03	0.03	0.07	1.00	-0.00	0.04	0.04	0.05	0.98	-0.00	0.03	0.03	0.06	1.00
MAR	NARFCS Int. X fully obs.	-0.00	0.03	0.03	0.06	1.00	-0.00	0.03	0.03	0.05	0.99	-0.00	0.03	0.03	0.05	1.00
MAR	MI RI	-0.07	0.13	0.15	0.29	1.00	-0.03	0.05	0.06	0.06	0.96	-0.04	0.07	0.08	0.14	1.00
MAR	MI RI X fully obs.	-0.06	0.10	0.12	0.26	1.00	-0.00	0.05	0.05	0.05	0.95	-0.03	0.06	0.07	0.13	1.00
Heckman, $R_x \sim X$	All cases	0.00	0.03	0.03	0.03	0.94	0.00	0.03	0.03	0.03	0.94	-0.00	0.03	0.03	0.03	0.96
Heckman, $R_x \sim X$	CC	0.00	0.06	0.06	0.06	0.96	0.00	0.05	0.05	0.05	0.94	0.00	0.06	0.06	0.06	0.95
Heckman, $R_x \sim X$	MI MAR	-0.08	0.06	0.10	0.06	0.76	-0.00	0.05	0.05	0.05	0.94	-0.04	0.05	0.06	0.06	0.90
Heckman, $R_x \sim X$	ML HE X cc	-0.00	0.11	0.11	0.10	0.93	0.00	0.05	0.05	0.05	0.94	0.00	0.07	0.07	0.07	0.95
Heckman, $R_x \sim X$	ML HE X excl.	-0.00	0.10	0.10	0.10	0.94	0.00	0.05	0.05	0.05	0.93	0.00	0.07	0.07	0.07	0.96
Heckman, $R_x \sim X$	ML HE X fully obs.	0.00	0.09	0.09	0.09	0.96	0.00	0.05	0.05	0.05	0.94	0.00	0.06	0.06	0.06	0.95
Heckman, $R_x \sim X$	UI X cc	-0.11	0.06	0.13	0.06	0.57	-0.01	0.05	0.05	0.05	0.94	-0.05	0.06	0.07	0.06	0.89
Heckman, $R_x \sim X$	UI X fully obs.	0.00	0.05	0.05	0.05	0.95	0.00	0.05	0.05	0.05	0.95	0.00	0.05	0.05	0.05	0.95
Heckman, $R_x \sim X$	MI HE X cc	-0.00	0.11	0.11	0.10	0.93	0.00	0.05	0.05	0.05	0.94	0.00	0.07	0.07	0.07	0.94
Heckman, $R_x \sim X$	MI HE	-0.01	0.09	0.09	0.10	0.98	0.00	0.05	0.05	0.05	0.94	-0.00	0.06	0.06	0.07	0.95
Heckman, $R_x \sim X$	MI HE X fully obs.	-0.00	0.09	0.09	0.09	0.95	0.00	0.05	0.05	0.05	0.94	0.00	0.06	0.06	0.06	0.95
Heckman, $R_x \sim X$	Stacking full data	-0.01	0.05	0.05	0.07	0.98	-0.00	0.05	0.05	0.05	0.94	0.00	0.05	0.05	0.06	0.96
Heckman, $R_x \sim X$	Stacking X MAR	-0.08	0.05	0.10	0.06	0.78	-0.00	0.05	0.05	0.05	0.94	-0.04	0.05	0.06	0.06	0.92
Heckman, $R_x \sim X$	Stacking X fully obs.	0.00	0.05	0.05	0.06	0.98	0.00	0.05	0.05	0.05	0.95	0.00	0.05	0.05	0.05	0.97
Heckman, $R_x \sim X$	NARFCS Shift SP	-0.12	0.10	0.16	0.07	0.52	-0.01	0.05	0.05	0.05	0.94	-0.05	0.07	0.09	0.06	0.79
Heckman, $R_x \sim X$	NARFCS Shift full data	0.01	0.05	0.05	0.07	0.99	0.00	0.05	0.05	0.05	0.94	0.01	0.05	0.05	0.06	0.98
Heckman, $R_x \sim X$	NARFCS Int. full data	0.00	0.04	0.04	0.07	1.00	0.00	0.04	0.04	0.05	0.98	0.00	0.03	0.03	0.06	1.00
Heckman, $R_x \sim X$	NARFCS Int. X fully obs.	0.00	0.03	0.03	0.06	1.00	0.00	0.03	0.03	0.05	0.99	0.00	0.03	0.03	0.05	1.00
Heckman, $R_x \sim X$	MI RI	-0.21	0.12	0.24	0.28	0.99	-0.05	0.05	0.07	0.08	0.96	-0.09	0.07	0.12	0.14	0.99
Heckman, $R_x \sim X$	MI RI X fully obs.	-0.06	0.11	0.12	0.26	1.00	0.00	0.05	0.05	0.05	0.96	-0.03	0.07	0.07	0.13	1.00
Heckman, $R_x \sim Y$	All cases	0.00	0.03	0.03	0.03	0.94	0.00	0.03	0.03	0.03	0.94	-0.00	0.03	0.03	0.03	0.95
Heckman, $R_x \sim Y$	CC	0.12	0.06	0.13	0.06	0.48	0.00	0.05	0.05	0.05	0.96	0.05	0.06	0.08	0.06	0.87
Heckman, $R_x \sim Y$	MI MAR	0.03	0.07	0.07	0.06	0.91	0.01	0.05	0.05	0.05	0.95	0.01	0.06	0.06	0.06	0.93
Heckman, $R_x \sim Y$	ML HE X cc	0.01	0.10	0.10	0.10	0.95	0.00	0.05	0.05	0.05	0.96	0.00	0.07	0.07	0.07	0.95
Heckman, $R_x \sim Y$	ML HE X excl.	0.02	0.09	0.09	0.10	0.96	0.00	0.05	0.05	0.05	0.96	0.00	0.07	0.07	0.07	0.95
Heckman, $R_x \sim Y$	ML HE X fully obs.	0.01	0.09	0.09	0.09	0.96	0.00	0.04	0.04	0.04	0.93	0.00	0.06	0.06	0.06	0.95
Heckman, $R_x \sim Y$	UI X cc	0.01	0.06	0.06	0.06	0.96	0.00	0.05	0.05	0.05	0.96	0.00	0.06	0.06	0.06	0.95
Heckman, $R_x \sim Y$	UI X fully obs.	0.00	0.05	0.05	0.05	0.96	0.00	0.04	0.04	0.04	0.94	0.00	0.05	0.05	0.05	0.94
Heckman, $R_x \sim Y$	MI HE X cc	0.01	0.10	0.10	0.10	0.95	0.00	0.05	0.05	0.05	0.96	0.00	0.07	0.07	0.07	0.95
Heckman, $R_x \sim Y$	MI HE	0.01	0.09	0.09	0.10	0.97	0.01	0.05	0.05	0.05	0.95	0.00	0.06	0.06	0.07	0.95
Heckman, $R_x \sim Y$	MI HE X fully obs.	0.00	0.09	0.09	0.09	0.95	0.00	0.05	0.05	0.05	0.94	0.00	0.06	0.06	0.06	0.94
Heckman, $R_x \sim Y$	Stacking full data	0.00	0.06	0.06	0.07	0.98	-0.00	0.05	0.05	0.05	0.94	0.01	0.06	0.06	0.06	0.95
Heckman, $R_x \sim Y$	Stacking X MAR	0.03	0.05	0.06	0.06	0.95	0.01	0.05	0.05	0.05	0.95	0.01	0.05	0.05	0.06	0.95
Heckman, $R_x \sim Y$	Stacking X fully obs.	0.00	0.05	0.05	0.06	0.99	0.00	0.05	0.05	0.05	0.94	0.00	0.05	0.05	0.05	0.96
Heckman, $R_x \sim Y$	NARFCS Shift SP	0.10	0.11	0.15	0.07	0.59	0.01	0.05	0.05	0.05	0.95	0.05	0.07	0.08	0.06	0.82
Heckman, $R_x \sim Y$	NARFCS Shift full data	-0.00	0.05	0.05	0.07	0.99	-0.00	0.05	0.05	0.05	0.94	-0.00	0.05	0.05	0.06	0.95
Heckman, $R_x \sim Y$	NARFCS Int. full data	-0.00	0.04	0.04	0.07	1.00	-0.00	0.04	0.04	0.05	0.98	-0.00	0.03	0.03	0.06	1.00
Heckman, $R_x \sim Y$	NARFCS Int. X fully obs.	0.00	0.03	0.03	0.06	1.00	0.00	0.03	0.03	0.05	0.99	-0.00	0.03	0.03	0.05	0.99
Heckman, $R_x \sim Y$	MI RI	-0.05	0.14	0.14	0.29	1.00	-0.02	0.05	0.06	0.07	0.98	-0.02	0.08	0.08	0.14	1.00
Heckman, $R_x \sim Y$	MI RI X fully obs.	-0.06	0.11	0.12	0.26	1.00	0.00	0.05	0.05	0.05	0.95	-0.03	0.07	0.07	0.13	1.00
Heckman, $R_x \sim X$ and Y	All cases	0.00	0.03	0.03	0.03	0.95	-0.00	0.03	0.03	0.03	0.95	0.00	0.03	0.03	0.03	0.94
Heckman, $R_x \sim X$ and Y	CC	0.12	0.06	0.13	0.06	0.49	-0.03	0.05	0.06	0.05	0.91	0.05	0.06	0.08	0.06	0.84
Heckman, $R_x \sim X$ and Y	MI MAR	-0.06	0.06	0.08	0.06	0.85	-0.02	0.05	0.06	0.05	0.92	-0.03	0.06	0.06	0.06	0.91
Heckman, $R_x \sim X$ and Y	ML HE X cc	0.01	0.10	0.10	0.10	0.93	-0.04	0.05	0.06	0.05	0.88	0.00	0.07	0.07	0.07	0.94
Heckman, $R_x \sim X$ and Y	ML HE X excl.	0.01	0.10	0.10	0.10	0.95	-0.03	0.05	0.06	0.05	0.90	0.00	0.07	0.07	0.07	0.94
Heckman, $R_x \sim X$ and Y	ML HE X fully obs.	0.00	0.09	0.09	0.09	0.94	-0.00	0.05	0.05	0.04	0.95	0.00	0.07	0.07	0.06	0.93
Heckman, $R_x \sim X$ and Y	UI X cc	0.01	0.06	0.06	0.06	0.95	-0.04	0.05	0.06	0.05	0.88	0.00	0.06	0.06	0.06	0.95
Heckman, $R_x \sim X$ and Y	UI X fully obs.	-0.00	0.05	0.05	0.05	0.95	-0.00	0.05	0.05	0.04	0.95	0.00	0.05	0.05	0.05	0.94
Heckman, $R_x \sim X$ and Y	MI HE X cc	0.01	0.10	0.10	0.10	0.94	-0.04	0.05	0.06	0.05	0.89	0.00	0.07	0.07	0.07	0.95
Heckman, $R_x \sim X$ and Y	MI HE	-0.00	0.09	0.09	0.10	0.96	-0.01	0.05	0.05	0.05	0.94	0.00	0.07	0.07	0.07	0.94
Heckman, $R_x \sim X$ and Y	MI HE X fully obs.	0.00	0.09	0.09	0.09	0.92	-0.00	0.05	0.05	0.05	0.95	0.00	0.07	0.07	0.06	0.93
Heckman, $R_x \sim X$ and Y	Stacking full data	-0.00	0.05	0.05	0.07	0.98	-0.01	0.05	0.05	0.05	0.94	0.01	0.06	0.06	0.06	0.95
Heckman, $R_x \sim X$ and Y	Stacking X MAR	-0.06	0.05	0.08	0.06	0.91	-0.02	0.05	0.06	0.05	0.92	-0.03	0.05	0.06	0.06	0.93
Heckman, $R_x \sim X$ and Y	Stacking X fully obs.	-0.00	0.04	0.04	0.06	0.99	-0.00	0.05	0.05	0.05	0.94	0.00	0.05	0.05	0.05	0.95
Heckman, $R_x \sim X$ and Y	NARFCS Shift SP	-0.04	0.10	0.11	0.07	0.78	-0.02	0.05	0.06	0.05	0.92	-0.02	0.07	0.07	0.06	0.89
Heckman, $R_x \sim X$ and Y	NARFCS Shift full data	0.00	0.05	0.05	0.07	0.99	-0.00	0.05	0.05	0.05	0.94	0.00	0.05	0.05	0.06	0.96
Heckman, $R_x \sim X$ and Y	NARFCS Int. full data	0.00	0.03	0.03	0.07	1.00	-0.00	0.04	0.04	0.05	0.99	0.00	0.04	0.04	0.06	1.00
Heckman, $R_x \sim X$ and Y	NARFCS Int. X fully obs.	0.00	0.03	0.03	0.06	1.00	-0.00	0.03	0.03	0.05	0.99	0.00	0.03	0.03	0.05	1.00
Heckman, $R_x \sim X$ and Y	MI RI	-0.19	0.13	0.23	0.29	0.99	-0.08	0.06	0.10	0.09	0.95	-0.09	0.08	0.12	0.14	0.98
Heckman, $R_x \sim X$ and Y	MI RI X fully obs.	-0.06	0.11	0.12	0.26	1.00	-0.00	0.05	0.05	0.05	0.95	-0.03	0.07	0.07	0.13	1.00

Table S8: (1)Performance of the methods in the simulation study with non-monotone missing. DGM: data-generating mechanism; empse: empirical standard error; rmse: root mean square error; modse: model standard error; cov: empirical coverage of the 95% confidence intervals; see the description of abbreviations in Table S2.

DGM	Method	Intercept					β_x					β_z				
		bias	empse	rmse	modse	cov	bias	empse	rmse	modse	cov	bias	empse	rmse	modse	cov
Heckman, $R_y\sim X$	All cases	0.00	0.03	0.03	0.03	0.95	-0.00	0.03	0.03	0.03	0.95	-0.00	0.03	0.03	0.03	0.94
Heckman, $R_y\sim X$	CC	0.00	0.06	0.06	0.06	0.95	-0.00	0.05	0.05	0.05	0.95	-0.00	0.06	0.06	0.06	0.95
Heckman, $R_y\sim X$	MI MAR	0.06	0.07	0.09	0.06	0.83	-0.01	0.05	0.05	0.05	0.94	0.03	0.06	0.07	0.06	0.90
Heckman, $R_y\sim X$	ML HE X cc	0.00	0.10	0.10	0.10	0.93	-0.00	0.06	0.06	0.05	0.95	-0.00	0.07	0.07	0.07	0.94
Heckman, $R_y\sim X$	ML HE X excl.	0.00	0.10	0.10	0.10	0.95	-0.00	0.05	0.05	0.05	0.94	-0.00	0.07	0.07	0.07	0.94
Heckman, $R_y\sim X$	ML HE X fully obs.	0.00	0.09	0.09	0.09	0.94	-0.00	0.05	0.05	0.05	0.95	-0.00	0.06	0.06	0.06	0.93
Heckman, $R_y\sim X$	UI X cc	-0.11	0.06	0.13	0.06	0.58	0.02	0.05	0.06	0.05	0.92	-0.05	0.06	0.08	0.06	0.87
Heckman, $R_y\sim X$	UI X fully obs.	0.00	0.05	0.05	0.05	0.95	-0.00	0.05	0.05	0.05	0.94	0.00	0.05	0.05	0.05	0.94
Heckman, $R_y\sim X$	MI HE X cc	0.00	0.11	0.11	0.10	0.93	-0.00	0.06	0.06	0.06	0.94	-0.00	0.07	0.07	0.07	0.94
Heckman, $R_y\sim X$	MI HE	-0.01	0.09	0.09	0.10	0.96	0.00	0.05	0.05	0.05	0.94	-0.00	0.06	0.06	0.07	0.94
Heckman, $R_y\sim X$	MI HE X fully obs.	-0.00	0.09	0.09	0.09	0.94	-0.00	0.05	0.05	0.05	0.94	-0.00	0.06	0.06	0.06	0.92
Heckman, $R_y\sim X$	Stacking full data	0.01	0.05	0.06	0.07	0.98	-0.00	0.05	0.05	0.05	0.94	0.00	0.06	0.06	0.06	0.96
Heckman, $R_y\sim X$	Stacking X MAR	0.06	0.05	0.08	0.06	0.88	-0.01	0.05	0.05	0.05	0.94	0.03	0.05	0.06	0.06	0.91
Heckman, $R_y\sim X$	Stacking X fully obs.	0.00	0.05	0.05	0.06	0.98	-0.00	0.05	0.05	0.05	0.94	-0.00	0.05	0.05	0.05	0.95
Heckman, $R_y\sim X$	NARFCS Shift SP	-0.01	0.11	0.11	0.07	0.78	-0.00	0.05	0.05	0.05	0.93	-0.00	0.07	0.07	0.06	0.89
Heckman, $R_y\sim X$	NARFCS Shift full data	0.00	0.05	0.05	0.07	0.99	-0.00	0.05	0.05	0.05	0.94	0.00	0.05	0.05	0.06	0.95
Heckman, $R_y\sim X$	NARFCS Int. full data	0.00	0.04	0.04	0.07	1.00	-0.00	0.04	0.04	0.05	0.98	0.00	0.03	0.03	0.06	1.00
Heckman, $R_y\sim X$	NARFCS Int. X fully obs.	0.00	0.03	0.03	0.06	1.00	-0.00	0.03	0.03	0.05	0.99	-0.00	0.03	0.03	0.05	1.00
Heckman, $R_y\sim X$	MI RI	0.04	0.14	0.14	0.29	1.00	-0.03	0.05	0.06	0.07	0.97	0.02	0.08	0.08	0.14	0.99
Heckman, $R_y\sim X$	MI RI X fully obs.	-0.04	0.11	0.12	0.26	1.00	0.01	0.05	0.05	0.06	0.97	-0.02	0.07	0.07	0.13	1.00
Heckman, $R_y\sim Y$	All cases	-0.00	0.03	0.03	0.03	0.95	0.00	0.03	0.03	0.03	0.94	-0.00	0.03	0.03	0.03	0.94
Heckman, $R_y\sim Y$	CC	0.12	0.06	0.13	0.06	0.51	0.00	0.05	0.05	0.05	0.94	0.05	0.06	0.08	0.06	0.88
Heckman, $R_y\sim Y$	MI MAR	0.23	0.06	0.24	0.06	0.06	-0.00	0.05	0.05	0.05	0.94	0.10	0.05	0.12	0.05	0.50
Heckman, $R_y\sim Y$	ML HE X cc	0.01	0.10	0.10	0.10	0.95	0.00	0.05	0.05	0.05	0.95	0.00	0.07	0.07	0.07	0.96
Heckman, $R_y\sim Y$	ML HE X excl.	0.02	0.09	0.10	0.10	0.95	0.00	0.05	0.05	0.05	0.95	0.00	0.07	0.07	0.07	0.96
Heckman, $R_y\sim Y$	ML HE X fully obs.	0.00	0.08	0.08	0.09	0.96	0.00	0.05	0.05	0.04	0.94	0.00	0.06	0.06	0.06	0.95
Heckman, $R_y\sim Y$	UI X cc	0.00	0.06	0.06	0.06	0.96	0.00	0.05	0.05	0.05	0.95	0.00	0.06	0.06	0.06	0.96
Heckman, $R_y\sim Y$	UI X fully obs.	0.00	0.05	0.05	0.05	0.96	0.00	0.05	0.05	0.05	0.95	0.00	0.05	0.05	0.05	0.96
Heckman, $R_y\sim Y$	MI HE X cc	0.01	0.10	0.10	0.10	0.94	0.00	0.05	0.05	0.05	0.95	0.00	0.07	0.07	0.07	0.95
Heckman, $R_y\sim Y$	MI HE	0.01	0.08	0.09	0.10	0.97	-0.00	0.05	0.05	0.05	0.95	0.00	0.06	0.06	0.06	0.95
Heckman, $R_y\sim Y$	MI HE X fully obs.	0.00	0.08	0.08	0.09	0.95	0.00	0.05	0.05	0.05	0.93	0.00	0.06	0.06	0.06	0.95
Heckman, $R_y\sim Y$	Stacking full data	0.01	0.05	0.05	0.07	0.97	-0.00	0.05	0.05	0.05	0.93	0.01	0.05	0.06	0.06	0.96
Heckman, $R_y\sim Y$	Stacking X MAR	-0.02	0.05	0.06	0.07	0.98	0.01	0.05	0.05	0.06	0.95	-0.01	0.05	0.05	0.06	0.97
Heckman, $R_y\sim Y$	Stacking X fully obs.	-0.02	0.05	0.05	0.06	0.99	0.00	0.05	0.05	0.05	0.95	-0.01	0.05	0.05	0.06	0.96
Heckman, $R_y\sim Y$	NARFCS Shift SP	0.34	0.11	0.36	0.07	0.03	-0.00	0.05	0.05	0.05	0.94	0.16	0.07	0.17	0.06	0.23
Heckman, $R_y\sim Y$	NARFCS Shift full data	-0.01	0.05	0.05	0.07	0.99	-0.00	0.05	0.05	0.05	0.94	-0.00	0.05	0.05	0.06	0.96
Heckman, $R_y\sim Y$	NARFCS Int. full data	-0.00	0.03	0.03	0.07	1.00	-0.00	0.04	0.04	0.05	0.99	-0.00	0.04	0.04	0.06	1.00
Heckman, $R_y\sim Y$	NARFCS Int. X fully obs.	-0.00	0.03	0.03	0.06	1.00	0.00	0.03	0.03	0.05	0.99	-0.00	0.03	0.03	0.05	1.00
Heckman, $R_y\sim Y$	MI RI	0.23	0.13	0.26	0.28	0.97	-0.03	0.05	0.06	0.06	0.97	0.10	0.07	0.13	0.14	0.97
Heckman, $R_y\sim Y$	MI RI X fully obs.	0.24	0.11	0.26	0.25	0.96	0.00	0.05	0.05	0.05	0.94	0.11	0.06	0.13	0.12	0.96
Heckman, $R_y\sim X$ and Y	All cases	-0.00	0.03	0.03	0.03	0.96	0.00	0.03	0.03	0.03	0.96	0.00	0.03	0.03	0.03	0.94
Heckman, $R_y\sim X$ and Y	CC	0.12	0.06	0.14	0.06	0.48	-0.02	0.05	0.06	0.05	0.93	0.05	0.06	0.08	0.06	0.86
Heckman, $R_y\sim X$ and Y	MI MAR	0.29	0.06	0.30	0.06	0.01	-0.04	0.05	0.06	0.05	0.89	0.14	0.05	0.15	0.06	0.28
Heckman, $R_y\sim X$ and Y	ML HE X cc	0.00	0.10	0.10	0.10	0.94	0.00	0.05	0.05	0.05	0.95	0.00	0.07	0.07	0.07	0.95
Heckman, $R_y\sim X$ and Y	ML HE X excl.	0.01	0.10	0.10	0.10	0.95	-0.03	0.05	0.06	0.05	0.92	0.00	0.07	0.07	0.07	0.95
Heckman, $R_y\sim X$ and Y	ML HE X fully obs.	0.00	0.09	0.09	0.08	0.93	0.00	0.05	0.05	0.05	0.95	0.00	0.06	0.06	0.06	0.95
Heckman, $R_y\sim X$ and Y	UI X cc	0.01	0.06	0.06	0.06	0.94	0.00	0.05	0.05	0.05	0.96	0.01	0.06	0.06	0.06	0.95
Heckman, $R_y\sim X$ and Y	UI X fully obs.	0.01	0.05	0.05	0.05	0.94	-0.00	0.04	0.04	0.05	0.96	0.00	0.05	0.05	0.05	0.96
Heckman, $R_y\sim X$ and Y	MI HE X cc	0.01	0.10	0.10	0.10	0.93	0.00	0.05	0.05	0.06	0.95	0.00	0.07	0.07	0.07	0.95
Heckman, $R_y\sim X$ and Y	MI HE	0.00	0.09	0.09	0.09	0.95	0.01	0.05	0.05	0.05	0.96	0.00	0.06	0.06	0.06	0.95
Heckman, $R_y\sim X$ and Y	MI HE X fully obs.	0.00	0.09	0.09	0.09	0.93	0.00	0.05	0.05	0.05	0.96	0.00	0.06	0.06	0.06	0.93
Heckman, $R_y\sim X$ and Y	Stacking full data	0.02	0.06	0.06	0.07	0.97	-0.01	0.05	0.05	0.05	0.95	0.01	0.06	0.06	0.06	0.96
Heckman, $R_y\sim X$ and Y	Stacking X MAR	0.03	0.05	0.06	0.07	0.96	-0.01	0.05	0.05	0.06	0.96	0.02	0.05	0.06	0.06	0.96
Heckman, $R_y\sim X$ and Y	Stacking X fully obs.	-0.02	0.05	0.05	0.07	0.99	0.00	0.05	0.05	0.05	0.97	-0.01	0.05	0.05	0.06	0.97
Heckman, $R_y\sim X$ and Y	NARFCS Shift SP	0.35	0.11	0.36	0.07	0.03	-0.04	0.05	0.07	0.05	0.86	0.16	0.07	0.17	0.06	0.21
Heckman, $R_y\sim X$ and Y	NARFCS Shift full data	-0.00	0.05	0.05	0.07	0.99	0.00	0.05	0.05	0.05	0.95	0.00	0.05	0.05	0.06	0.96
Heckman, $R_y\sim X$ and Y	NARFCS Int. full data	0.00	0.03	0.03	0.07	1.00	0.00	0.04	0.04	0.05	0.99	0.00	0.03	0.03	0.06	1.00
Heckman, $R_y\sim X$ and Y	NARFCS Int. X fully obs.	0.00	0.03	0.03	0.06	1.00	0.00	0.03	0.03	0.05	0.99	0.00	0.03	0.03	0.05	1.00
Heckman, $R_y\sim X$ and Y	MI RI	0.35	0.12	0.37	0.28	0.87	-0.08	0.05	0.09	0.08	0.94	0.17	0.07	0.18	0.14	0.87
Heckman, $R_y\sim X$ and Y	MI RI X fully obs.	0.28	0.10	0.30	0.25	0.92	-0.04	0.05	0.06	0.06	0.94	0.13	0.06	0.15	0.12	0.92
Heckman, $R_x\sim X, R_y\sim X$	All cases	-0.00	0.03	0.03	0.03	0.95	-0.00	0.03	0.03	0.03	0.95	-0.00	0.03	0.03	0.03	0.94
Heckman, $R_x\sim X, R_y\sim X$	CC	-0.00	0.06	0.06	0.06	0.95	0.00	0.05	0.05	0.05	0.94	-0.00	0.06	0.06	0.06	0.94
Heckman, $R_x\sim X, R_y\sim X$	MI MAR	-0.03	0.07	0.07	0.07	0.92	0.00	0.05	0.05	0.05	0.94	-0.01	0.05	0.06	0.06	0.94
Heckman, $R_x\sim X, R_y\sim X$	ML HE X cc	-0.01	0.11	0.11	0.10	0.94	0.00	0.05	0.05	0.05	0.95	-0.00	0.07	0.07	0.07	0.95
Heckman, $R_x\sim X, R_y\sim X$	ML HE X excl.	-0.01	0.10	0.10	0.10	0.95	0.00	0.05	0.05	0.05	0.94	-0.00	0.07	0.07	0.07	0.95
Heckman, $R_x\sim X, R_y\sim X$	ML HE X fully obs.	-0.01	0.09	0.09	0.09	0.96	0.00	0.05	0.05	0.05	0.94	-0.00	0.06	0.06	0.06	0.95
Heckman, $R_x\sim X, R_y\sim X$	UI X cc	-0.12	0.06	0.13	0.06	0.54	0.02	0.05	0.05	0.05	0.93	-0.05	0.06	0.08	0.06	0.88
Heckman, $R_x\sim X, R_y\sim X$	UI X fully obs.	-0.00	0.05	0.05	0.05	0.95	0.00	0.05	0.05	0.05	0.94	0.00	0.05	0.05	0.05	0.95
Heckman, $R_x\sim X, R_y\sim X$	MI HE X cc	-0.01	0.11	0.11	0.10	0.93	0.00	0.05	0.05	0.05	0.95	-0.00	0.07	0.07	0.07	0.95
Heckman, $R_x\sim X, R_y\sim X$	MI HE	-0.02	0.09	0.09	0.10	0.96	-0.00	0.05	0.05	0.05	0.94	-0.01	0.06	0.06	0.07	0.95
Heckman, $R_x\sim X, R_y\sim X$	MI HE X fully obs.	-0.01	0.09	0.09	0.09	0.95	0.00	0.05	0.05	0.05	0.94	-0.00	0.06	0.06	0.06	0.95
Heckman, $R_x\sim X, R_y\sim X$	Stacking full data	-0.00	0.05	0.05	0.07	0.98	0.00	0.05	0.05	0.06	0.96	0.00	0.06	0.06	0.06	0.96
Heckman, $R_x\sim X, R_y\sim X$	Stacking X MAR	-0.03	0.05	0.06	0.07	0.96	0.00	0.05	0.05	0.05	0.95	-0.01	0.05	0.05	0.06	0.96
Heckman, $R_x\sim X, R_y\sim X$	Stacking X fully obs.	-0.00	0.05	0.05	0.06	0.99	0.00	0.05	0.05	0.05	0.95	0.00	0.05	0.05	0.05	0.96
Heckman, $R_x\sim X, R_y\sim X$	NARFCS Shift SP	-0.14	0.11	0.18	0.07	0.48	-0.01	0.05	0.05	0.05	0.94	-0.06	0.07	0.09	0.06	0.76
Heckman, $R_x\sim X, R_y\sim X$	NARFCS Shift full data	0.01	0.05	0.05	0.07	0.99	0.00	0.05	0.05	0.05	0.95	0.01	0.05	0.05	0.06	0.96
Heckman, $R_x\sim X, R_y\sim X$	NARFCS Int. full data	0.00	0.04	0.04	0.07	1.00	-0.00	0.04	0.04	0.05	0.98	0.01	0.04	0.04	0.06	1.00
Heckman, $R_x\sim X, R_y\sim X$	NARFCS Int. X fully obs.	-0.00	0.03	0.03	0.06	1.00	-0.00	0.03	0.03	0.05	0.99	-0.00	0.03	0.03	0.05	1.00
Heckman, $R_x\sim X, R_y\sim X$	MI RI	-0.10	0.13	0.16	0.29	1.00	-0.03	0.05	0.06	0.07	0.98	-0.04	0.08	0.09	0.14	0.99
Heckman, $R_x\sim X, R_y\sim X$	MI RI X fully obs.	-0.04	0.11	0.12	0.26	1.00	0.01	0.05	0.05	0.06	0.97	-0.02	0.07	0.07	0.13	1.00

Table S9: (2)Performance of the methods in the simulation study with non-monotone missing. DGM: data-generating mechanism; empse: empirical standard error; rmse: root mean square error; modse: model standard error; cov: empirical coverage of the 95% confidence intervals; see the description of abbreviations in Table S2.

		Intercept					β_x					β_z				
DGM	Method	bias	empse	rmse	modse	cov	bias	empse	rmse	modse	cov	bias	empse	rmse	modse	cov
Heckman, $R_x \sim X$, $R_y \sim Y$	All cases	0.00	0.03	0.03	0.03	0.95	-0.00	0.03	0.03	0.03	0.95	0.00	0.03	0.03	0.03	0.95
Heckman, $R_x \sim X$, $R_y \sim Y$	CC	0.12	0.06	0.13	0.06	0.53	0.01	0.05	0.05	0.05	0.95	0.05	0.06	0.08	0.06	0.85
Heckman, $R_x \sim X$, $R_y \sim Y$	MI MAR	0.14	0.06	0.15	0.06	0.38	0.01	0.05	0.05	0.05	0.95	0.06	0.06	0.08	0.05	0.75
Heckman, $R_x \sim X$, $R_y \sim Y$	ML HE X cc	0.00	0.11	0.11	0.10	0.93	-0.00	0.05	0.05	0.05	0.96	0.00	0.07	0.07	0.07	0.95
Heckman, $R_x \sim X$, $R_y \sim Y$	ML HE X excl.	0.01	0.10	0.10	0.10	0.94	0.00	0.05	0.05	0.05	0.95	0.00	0.07	0.07	0.07	0.94
Heckman, $R_x \sim X$, $R_y \sim Y$	ML HE X fully obs.	0.00	0.09	0.09	0.09	0.95	-0.00	0.04	0.04	0.04	0.95	0.00	0.06	0.06	0.06	0.95
Heckman, $R_x \sim X$, $R_y \sim Y$	UI X cc	0.00	0.06	0.06	0.06	0.95	-0.00	0.05	0.05	0.05	0.96	0.00	0.06	0.06	0.06	0.94
Heckman, $R_x \sim X$, $R_y \sim Y$	UI X fully obs.	-0.00	0.05	0.05	0.05	0.95	-0.00	0.04	0.04	0.05	0.96	0.00	0.05	0.05	0.05	0.95
Heckman, $R_x \sim X$, $R_y \sim Y$	MI HE X cc	0.01	0.11	0.11	0.10	0.93	-0.00	0.05	0.05	0.05	0.96	0.00	0.07	0.07	0.07	0.94
Heckman, $R_x \sim X$, $R_y \sim Y$	MI HE	-0.00	0.09	0.09	0.10	0.96	-0.00	0.05	0.05	0.05	0.94	-0.00	0.06	0.06	0.07	0.95
Heckman, $R_x \sim X$, $R_y \sim Y$	MI HE X fully obs.	0.00	0.09	0.09	0.09	0.92	-0.00	0.05	0.05	0.05	0.95	0.00	0.06	0.06	0.06	0.94
Heckman, $R_x \sim X$, $R_y \sim Y$	Stacking full data	-0.01	0.05	0.05	0.07	0.98	-0.01	0.05	0.05	0.05	0.95	0.00	0.06	0.06	0.06	0.95
Heckman, $R_x \sim X$, $R_y \sim Y$	Stacking X MAR	-0.11	0.05	0.12	0.07	0.70	0.00	0.05	0.05	0.06	0.96	-0.05	0.05	0.07	0.06	0.89
Heckman, $R_x \sim X$, $R_y \sim Y$	Stacking X fully obs.	-0.01	0.05	0.05	0.06	0.98	-0.00	0.05	0.05	0.05	0.96	-0.01	0.05	0.05	0.06	0.95
Heckman, $R_x \sim X$, $R_y \sim Y$	NARFCS Shift SP	0.20	0.10	0.23	0.07	0.23	0.02	0.05	0.05	0.05	0.93	0.09	0.07	0.12	0.06	0.59
Heckman, $R_x \sim X$, $R_y \sim Y$	NARFCS Shift full data	0.00	0.05	0.05	0.07	1.00	-0.00	0.05	0.05	0.05	0.95	0.00	0.05	0.05	0.06	0.95
Heckman, $R_x \sim X$, $R_y \sim Y$	NARFCS Int. full data	0.00	0.03	0.03	0.07	1.00	-0.00	0.04	0.04	0.05	0.98	0.00	0.03	0.03	0.06	0.99
Heckman, $R_x \sim X$, $R_y \sim Y$	NARFCS Int. X fully obs.	0.00	0.03	0.03	0.06	1.00	-0.00	0.03	0.03	0.05	0.99	0.00	0.03	0.03	0.05	1.00
Heckman, $R_x \sim X$, $R_y \sim Y$	MI RI	0.09	0.12	0.15	0.27	1.00	-0.02	0.05	0.05	0.07	0.98	0.04	0.07	0.08	0.14	1.00
Heckman, $R_x \sim X$, $R_y \sim Y$	MI RI X fully obs.	0.24	0.10	0.26	0.25	0.97	-0.00	0.04	0.04	0.05	0.95	0.11	0.06	0.13	0.12	0.96
Heckman, $R_x \sim X$, $R_y \sim X$ and Y	All cases	0.00	0.03	0.03	0.03	0.94	-0.00	0.03	0.03	0.03	0.95	-0.00	0.03	0.03	0.03	0.95
Heckman, $R_x \sim X$, $R_y \sim X$ and Y	CC	0.12	0.06	0.14	0.06	0.51	-0.01	0.05	0.05	0.05	0.94	0.05	0.06	0.08	0.06	0.86
Heckman, $R_x \sim X$, $R_y \sim X$ and Y	MI MAR	0.21	0.07	0.22	0.06	0.12	-0.02	0.05	0.05	0.05	0.94	0.10	0.05	0.11	0.05	0.55
Heckman, $R_x \sim X$, $R_y \sim X$ and Y	ML HE X cc	0.00	0.10	0.10	0.10	0.94	0.00	0.05	0.05	0.05	0.95	0.00	0.07	0.07	0.07	0.95
Heckman, $R_x \sim X$, $R_y \sim X$ and Y	ML HE X excl.	0.01	0.10	0.10	0.10	0.94	-0.02	0.05	0.06	0.05	0.92	0.00	0.07	0.07	0.07	0.95
Heckman, $R_x \sim X$, $R_y \sim X$ and Y	ML HE X fully obs.	0.00	0.08	0.09	0.08	0.95	-0.00	0.05	0.05	0.05	0.96	0.00	0.06	0.06	0.06	0.96
Heckman, $R_x \sim X$, $R_y \sim X$ and Y	UI X cc	0.01	0.06	0.07	0.06	0.95	0.00	0.05	0.05	0.05	0.96	0.00	0.06	0.06	0.06	0.95
Heckman, $R_x \sim X$, $R_y \sim X$ and Y	UI X fully obs.	0.01	0.05	0.05	0.05	0.95	-0.00	0.04	0.04	0.05	0.96	0.00	0.05	0.05	0.05	0.95
Heckman, $R_x \sim X$, $R_y \sim X$ and Y	MI HE X cc	0.01	0.10	0.10	0.10	0.95	0.00	0.05	0.05	0.05	0.96	0.00	0.07	0.07	0.07	0.95
Heckman, $R_x \sim X$, $R_y \sim X$ and Y	MI HE	-0.01	0.09	0.09	0.10	0.94	-0.01	0.05	0.05	0.05	0.96	-0.00	0.06	0.06	0.06	0.96
Heckman, $R_x \sim X$, $R_y \sim X$ and Y	MI HE X fully obs.	0.00	0.09	0.09	0.09	0.94	-0.00	0.05	0.05	0.05	0.96	0.00	0.06	0.06	0.06	0.95
Heckman, $R_x \sim X$, $R_y \sim X$ and Y	Stacking full data	0.01	0.06	0.06	0.07	0.99	-0.01	0.05	0.05	0.06	0.96	0.01	0.05	0.06	0.06	0.96
Heckman, $R_x \sim X$, $R_y \sim X$ and Y	Stacking X MAR	-0.06	0.05	0.08	0.07	0.92	-0.01	0.05	0.05	0.06	0.96	-0.02	0.05	0.06	0.06	0.95
Heckman, $R_x \sim X$, $R_y \sim X$ and Y	Stacking X fully obs.	-0.02	0.05	0.05	0.07	0.98	0.00	0.05	0.05	0.05	0.96	-0.01	0.05	0.05	0.06	0.96
Heckman, $R_x \sim X$, $R_y \sim X$ and Y	NARFCS Shift SP	0.22	0.11	0.24	0.07	0.23	-0.03	0.05	0.06	0.05	0.92	0.10	0.07	0.12	0.06	0.54
Heckman, $R_x \sim X$, $R_y \sim X$ and Y	NARFCS Shift full data	0.00	0.05	0.05	0.07	0.99	0.00	0.05	0.05	0.05	0.95	0.00	0.05	0.05	0.06	0.97
Heckman, $R_x \sim X$, $R_y \sim X$ and Y	NARFCS Int. full data	0.00	0.03	0.03	0.07	1.00	0.00	0.04	0.04	0.05	0.99	0.00	0.03	0.03	0.06	1.00
Heckman, $R_x \sim X$, $R_y \sim X$ and Y	NARFCS Int. X fully obs.	0.00	0.03	0.03	0.06	1.00	-0.00	0.03	0.03	0.05	1.00	-0.00	0.03	0.03	0.05	1.00
Heckman, $R_x \sim X$, $R_y \sim X$ and Y	MI RI	0.21	0.12	0.25	0.28	0.98	-0.04	0.05	0.06	0.06	0.94	0.10	0.07	0.12	0.14	0.97
Heckman, $R_x \sim X$, $R_y \sim X$ and Y	MI RI X fully obs.	0.28	0.11	0.30	0.25	0.93	-0.04	0.05	0.06	0.06	0.93	0.13	0.06	0.14	0.12	0.92
Heckman, $R_x \sim Y$, $R_y \sim X$	All cases	0.00	0.03	0.03	0.03	0.96	-0.00	0.03	0.03	0.03	0.95	-0.00	0.03	0.03	0.03	0.95
Heckman, $R_x \sim Y$, $R_y \sim X$	CC	0.12	0.06	0.13	0.06	0.51	0.01	0.05	0.05	0.05	0.95	0.05	0.06	0.08	0.06	0.86
Heckman, $R_x \sim Y$, $R_y \sim X$	MI MAR	0.09	0.06	0.11	0.06	0.70	0.01	0.05	0.05	0.05	0.94	0.04	0.06	0.07	0.05	0.87
Heckman, $R_x \sim Y$, $R_y \sim X$	ML HE X cc	0.01	0.10	0.10	0.10	0.93	0.03	0.05	0.06	0.05	0.90	0.00	0.07	0.07	0.07	0.94
Heckman, $R_x \sim Y$, $R_y \sim X$	ML HE X excl.	0.01	0.10	0.10	0.10	0.93	0.01	0.05	0.05	0.05	0.95	0.00	0.07	0.07	0.07	0.95
Heckman, $R_x \sim Y$, $R_y \sim X$	ML HE X fully obs.	0.00	0.09	0.09	0.09	0.94	-0.00	0.05	0.05	0.05	0.95	0.00	0.06	0.06	0.06	0.95
Heckman, $R_x \sim Y$, $R_y \sim X$	UI X cc	0.01	0.06	0.06	0.06	0.96	0.03	0.05	0.06	0.05	0.90	0.00	0.06	0.06	0.06	0.94
Heckman, $R_x \sim Y$, $R_y \sim X$	UI X fully obs.	0.00	0.05	0.05	0.05	0.96	0.00	0.04	0.04	0.05	0.96	0.00	0.05	0.05	0.05	0.95
Heckman, $R_x \sim Y$, $R_y \sim X$	MI HE X cc	0.01	0.10	0.10	0.10	0.93	0.03	0.05	0.06	0.06	0.91	0.01	0.07	0.07	0.07	0.94
Heckman, $R_x \sim Y$, $R_y \sim X$	MI HE	0.00	0.09	0.09	0.10	0.96	0.02	0.05	0.05	0.05	0.94	0.00	0.06	0.06	0.07	0.96
Heckman, $R_x \sim Y$, $R_y \sim X$	MI HE X fully obs.	0.00	0.09	0.09	0.09	0.93	0.00	0.05	0.05	0.05	0.95	0.00	0.06	0.06	0.06	0.94
Heckman, $R_x \sim Y$, $R_y \sim X$	Stacking full data	0.01	0.06	0.06	0.07	0.98	-0.01	0.05	0.05	0.05	0.95	0.01	0.06	0.06	0.06	0.95
Heckman, $R_x \sim Y$, $R_y \sim X$	Stacking X MAR	0.09	0.05	0.10	0.06	0.75	0.01	0.05	0.05	0.05	0.95	0.04	0.05	0.07	0.06	0.88
Heckman, $R_x \sim Y$, $R_y \sim X$	Stacking X fully obs.	0.00	0.05	0.05	0.06	0.99	0.00	0.05	0.05	0.05	0.95	0.00	0.05	0.05	0.05	0.95
Heckman, $R_x \sim Y$, $R_y \sim X$	NARFCS Shift SP	0.09	0.11	0.14	0.07	0.64	0.00	0.05	0.05	0.05	0.94	0.04	0.07	0.08	0.06	0.84
Heckman, $R_x \sim Y$, $R_y \sim X$	NARFCS Shift full data	0.00	0.05	0.05	0.07	1.00	-0.00	0.05	0.05	0.05	0.94	0.00	0.05	0.05	0.06	0.97
Heckman, $R_x \sim Y$, $R_y \sim X$	NARFCS Int. full data	0.00	0.03	0.03	0.07	1.00	-0.00	0.04	0.04	0.05	0.99	0.00	0.03	0.03	0.06	1.00
Heckman, $R_x \sim Y$, $R_y \sim X$	NARFCS Int. X fully obs.	0.00	0.03	0.03	0.06	1.00	0.00	0.03	0.03	0.05	0.99	-0.00	0.03	0.03	0.05	1.00
Heckman, $R_x \sim Y$, $R_y \sim X$	MI RI	0.07	0.13	0.15	0.29	1.00	-0.01	0.05	0.05	0.07	0.98	0.03	0.08	0.08	0.14	1.00
Heckman, $R_x \sim Y$, $R_y \sim X$	MI RI X fully obs.	-0.04	0.12	0.12	0.26	1.00	0.01	0.05	0.05	0.06	0.97	-0.02	0.07	0.07	0.13	1.00
Heckman, $R_x \sim Y$, $R_y \sim Y$	All cases	0.00	0.03	0.03	0.03	0.96	-0.00	0.03	0.03	0.03	0.95	-0.00	0.03	0.03	0.03	0.95
Heckman, $R_x \sim Y$, $R_y \sim Y$	CC	0.24	0.06	0.24	0.06	0.03	-0.00	0.05	0.05	0.05	0.96	0.10	0.06	0.11	0.06	0.60
Heckman, $R_x \sim Y$, $R_y \sim Y$	MI MAR	0.26	0.06	0.27	0.06	0.02	0.00	0.05	0.05	0.05	0.96	0.11	0.05	0.13	0.05	0.45
Heckman, $R_x \sim Y$, $R_y \sim Y$	ML HE X cc	0.04	0.08	0.09	0.09	0.95	-0.00	0.05	0.05	0.05	0.95	0.01	0.06	0.06	0.07	0.95
Heckman, $R_x \sim Y$, $R_y \sim Y$	ML HE X excl.	0.03	0.09	0.09	0.09	0.93	-0.00	0.05	0.05	0.05	0.96	0.00	0.07	0.07	0.07	0.94
Heckman, $R_x \sim Y$, $R_y \sim Y$	ML HE X fully obs.	0.00	0.08	0.08	0.09	0.95	-0.00	0.04	0.04	0.04	0.96	-0.00	0.06	0.06	0.06	0.95
Heckman, $R_x \sim Y$, $R_y \sim Y$	UI X cc	0.12	0.06	0.14	0.06	0.49	-0.00	0.05	0.05	0.05	0.95	0.05	0.06	0.08	0.06	0.87
Heckman, $R_x \sim Y$, $R_y \sim Y$	UI X fully obs.	0.00	0.05	0.05	0.05	0.97	-0.00	0.04	0.04	0.05	0.96	-0.00	0.05	0.05	0.05	0.95
Heckman, $R_x \sim Y$, $R_y \sim Y$	MI HE X cc	0.04	0.08	0.09	0.09	0.94	-0.00	0.05	0.05	0.05	0.96	0.01	0.06	0.07	0.07	0.95
Heckman, $R_x \sim Y$, $R_y \sim Y$	MI HE	0.02	0.08	0.09	0.10	0.96	-0.00	0.05	0.05	0.05	0.96	0.00	0.06	0.06	0.06	0.96
Heckman, $R_x \sim Y$, $R_y \sim Y$	MI HE X fully obs.	0.00	0.09	0.09	0.09	0.94	0.00	0.04	0.04	0.05	0.95	-0.00	0.06	0.06	0.06	0.95
Heckman, $R_x \sim Y$, $R_y \sim Y$	Stacking full data	0.01	0.05	0.06	0.07	0.97	-0.00	0.05	0.05	0.06	0.97	0.01	0.06	0.06	0.06	0.96
Heckman, $R_x \sim Y$, $R_y \sim Y$	Stacking X MAR	0.01	0.05	0.05	0.07	0.97	0.01	0.05	0.05	0.06	0.97	-0.00	0.05	0.05	0.06	0.96
Heckman, $R_x \sim Y$, $R_y \sim Y$	Stacking X fully obs.	-0.01	0.04	0.05	0.07	0.99	-0.00	0.04	0.04	0.05	0.96	-0.01	0.05	0.05	0.06	0.97
Heckman, $R_x \sim Y$, $R_y \sim Y$	NARFCS Shift SP	0.44	0.10	0.45	0.07	0.00	-0.01	0.05	0.05	0.05	0.94	0.20	0.07	0.21	0.06	0.09
Heckman, $R_x \sim Y$, $R_y \sim Y$	NARFCS Shift full data	-0.00	0.05	0.05	0.07	0.99	-0.00	0.05	0.05	0.05	0.95	-0.01	0.05	0.05	0.06	0.96
Heckman, $R_x \sim Y$, $R_y \sim Y$	NARFCS Int. full data	-0.00	0.03	0.03	0.07	1.00	-0.00	0.04	0.04	0.05	0.99	-0.01	0.03	0.03	0.06	1.00
Heckman, $R_x \sim Y$, $R_y \sim Y$	NARFCS Int. X fully obs.	0.00	0.03	0.03	0.06	1.00	-0.00	0.03	0.03	0.05	0.99	-0.00	0.03	0.03	0.05	1.00
Heckman, $R_x \sim Y$, $R_y \sim Y$	MI RI	0.25	0.13	0.28	0.28	0.96	-0.03	0.05	0.05	0.06	0.97	0.11	0.07	0.13	0.14	0.96
Heckman, $R_x \sim Y$, $R_y \sim Y$	MI RI X fully obs.	0.24	0.11	0.26	0.25	0.97	-0.00	0.04	0.04	0.05	0.96	0.11	0.07	0.13	0.12	0.95

Table S10: (3)Performance of the methods in the simulation study with non-monotone missing. DGM: data-generating mechanism; empse: empirical standard error; rmse: root mean square error; modse: model standard error; cov: empirical coverage of the 95% confidence intervals; see the description of abbreviations in Table S2.

		Intercept					β_x					β_z				
DGM	Method	bias	empse	rmse	modse	cov	bias	empse	rmse	modse	cov	bias	empse	rmse	modse	cov
Heckman, $R_x \sim Y$, $R_y \sim X$ and Y	All cases	-0.00	0.03	0.03	0.03	0.95	0.00	0.03	0.03	0.03	0.95	-0.00	0.03	0.03	0.03	0.95
Heckman, $R_x \sim Y$, $R_y \sim X$ and Y	CC	0.24	0.06	0.25	0.06	0.02	-0.01	0.05	0.05	0.05	0.94	0.10	0.06	0.12	0.06	0.59
Heckman, $R_x \sim Y$, $R_y \sim X$ and Y	MI MAR	0.32	0.06	0.33	0.06	0.00	-0.02	0.05	0.05	0.05	0.92	0.15	0.05	0.16	0.05	0.21
Heckman, $R_x \sim Y$, $R_y \sim X$ and Y	ML HE X cc	0.04	0.08	0.09	0.09	0.94	0.03	0.05	0.06	0.05	0.92	0.02	0.06	0.07	0.06	0.95
Heckman, $R_x \sim Y$, $R_y \sim X$ and Y	ML HE X excl.	0.03	0.09	0.09	0.09	0.94	-0.02	0.05	0.06	0.05	0.92	0.00	0.07	0.07	0.07	0.96
Heckman, $R_x \sim Y$, $R_y \sim X$ and Y	ML HE X fully obs.	-0.00	0.08	0.08	0.08	0.96	0.00	0.05	0.05	0.05	0.95	-0.00	0.06	0.06	0.06	0.95
Heckman, $R_x \sim Y$, $R_y \sim X$ and Y	UI X cc	0.13	0.06	0.14	0.06	0.46	0.01	0.05	0.05	0.05	0.95	0.05	0.06	0.08	0.06	0.85
Heckman, $R_x \sim Y$, $R_y \sim X$ and Y	UI X fully obs.	0.01	0.05	0.05	0.05	0.95	-0.00	0.05	0.05	0.05	0.95	0.00	0.05	0.05	0.05	0.95
Heckman, $R_x \sim Y$, $R_y \sim X$ and Y	MI HE X cc	0.05	0.08	0.09	0.09	0.93	0.03	0.05	0.06	0.06	0.93	0.02	0.06	0.07	0.07	0.95
Heckman, $R_x \sim Y$, $R_y \sim X$ and Y	MI HE	0.00	0.08	0.08	0.09	0.96	0.02	0.05	0.05	0.05	0.94	0.00	0.06	0.06	0.06	0.95
Heckman, $R_x \sim Y$, $R_y \sim X$ and Y	MI HE X fully obs.	-0.00	0.08	0.08	0.09	0.95	0.00	0.05	0.05	0.05	0.94	-0.00	0.06	0.06	0.06	0.94
Heckman, $R_x \sim Y$, $R_y \sim X$ and Y	Stacking full data	0.02	0.06	0.06	0.07	0.96	-0.01	0.05	0.05	0.06	0.96	0.02	0.06	0.06	0.06	0.96
Heckman, $R_x \sim Y$, $R_y \sim X$ and Y	Stacking X MAR	0.06	0.05	0.08	0.07	0.88	0.00	0.05	0.05	0.06	0.97	0.03	0.05	0.06	0.06	0.94
Heckman, $R_x \sim Y$, $R_y \sim X$ and Y	Stacking X fully obs.	-0.02	0.05	0.05	0.07	0.98	0.00	0.05	0.05	0.05	0.96	-0.01	0.05	0.05	0.06	0.97
Heckman, $R_x \sim Y$, $R_y \sim X$ and Y	NARFCS Shift SP	0.44	0.10	0.45	0.07	0.00	-0.06	0.05	0.08	0.05	0.79	0.20	0.06	0.21	0.06	0.08
Heckman, $R_x \sim Y$, $R_y \sim X$ and Y	NARFCS Shift full data	-0.00	0.05	0.05	0.07	0.99	0.00	0.05	0.05	0.05	0.95	-0.00	0.05	0.05	0.06	0.96
Heckman, $R_x \sim Y$, $R_y \sim X$ and Y	NARFCS Int. full data	-0.00	0.04	0.04	0.07	1.00	0.00	0.04	0.04	0.05	0.99	-0.00	0.03	0.03	0.06	1.00
Heckman, $R_x \sim Y$, $R_y \sim X$ and Y	NARFCS Int. X fully obs.	-0.00	0.03	0.03	0.06	1.00	0.00	0.03	0.03	0.05	0.99	-0.00	0.03	0.03	0.05	1.00
Heckman, $R_x \sim Y$, $R_y \sim X$ and Y	MI RI	0.38	0.12	0.40	0.27	0.80	-0.05	0.05	0.08	0.07	0.93	0.18	0.07	0.19	0.13	0.82
Heckman, $R_x \sim Y$, $R_y \sim X$ and Y	MI RI X fully obs.	0.28	0.11	0.30	0.25	0.92	-0.04	0.05	0.06	0.06	0.93	0.13	0.07	0.15	0.12	0.92
Heckman, $R_x \sim X$ and Y, $R_y \sim X$	All cases	0.00	0.03	0.03	0.03	0.94	0.00	0.03	0.03	0.03	0.94	0.00	0.03	0.03	0.03	0.95
Heckman, $R_x \sim X$ and Y, $R_y \sim X$	CC	0.12	0.06	0.14	0.06	0.50	-0.02	0.05	0.05	0.05	0.95	0.05	0.06	0.08	0.06	0.86
Heckman, $R_x \sim X$ and Y, $R_y \sim X$	MI MAR	0.00	0.06	0.06	0.06	0.94	-0.00	0.05	0.05	0.05	0.95	0.01	0.05	0.05	0.06	0.94
Heckman, $R_x \sim X$ and Y, $R_y \sim X$	ML HE X cc	0.00	0.10	0.10	0.10	0.95	-0.00	0.05	0.05	0.05	0.96	-0.00	0.07	0.07	0.07	0.95
Heckman, $R_x \sim X$ and Y, $R_y \sim X$	ML HE X excl.	0.01	0.09	0.09	0.10	0.96	-0.03	0.05	0.06	0.05	0.92	0.00	0.07	0.07	0.07	0.96
Heckman, $R_x \sim X$ and Y, $R_y \sim X$	ML HE X fully obs.	-0.00	0.08	0.08	0.09	0.95	0.00	0.05	0.05	0.05	0.95	-0.00	0.06	0.06	0.06	0.95
Heckman, $R_x \sim X$ and Y, $R_y \sim X$	UI X cc	0.01	0.06	0.06	0.06	0.95	-0.00	0.05	0.05	0.05	0.96	0.00	0.06	0.06	0.06	0.95
Heckman, $R_x \sim X$ and Y, $R_y \sim X$	UI X fully obs.	0.00	0.05	0.05	0.05	0.96	0.00	0.04	0.04	0.05	0.95	0.00	0.05	0.05	0.05	0.96
Heckman, $R_x \sim X$ and Y, $R_y \sim X$	MI HE X cc	0.00	0.10	0.10	0.10	0.94	-0.00	0.05	0.05	0.05	0.96	0.00	0.07	0.07	0.07	0.95
Heckman, $R_x \sim X$ and Y, $R_y \sim X$	MI HE	-0.01	0.09	0.09	0.10	0.96	-0.01	0.05	0.05	0.05	0.94	-0.00	0.06	0.06	0.07	0.95
Heckman, $R_x \sim X$ and Y, $R_y \sim X$	MI HE X fully obs.	-0.00	0.09	0.09	0.09	0.94	0.00	0.05	0.05	0.05	0.95	0.00	0.06	0.06	0.06	0.95
Heckman, $R_x \sim X$ and Y, $R_y \sim X$	Stacking full data	0.00	0.06	0.06	0.07	0.98	-0.01	0.05	0.05	0.06	0.96	0.01	0.06	0.06	0.06	0.96
Heckman, $R_x \sim X$ and Y, $R_y \sim X$	Stacking X MAR	0.00	0.05	0.05	0.06	0.98	-0.00	0.05	0.05	0.05	0.95	0.00	0.05	0.05	0.06	0.96
Heckman, $R_x \sim X$ and Y, $R_y \sim X$	Stacking X fully obs.	-0.00	0.05	0.05	0.06	0.98	0.00	0.05	0.05	0.05	0.95	-0.00	0.05	0.05	0.05	0.97
Heckman, $R_x \sim X$ and Y, $R_y \sim X$	NARFCS Shift SP	-0.04	0.10	0.11	0.07	0.77	-0.03	0.05	0.06	0.05	0.91	-0.01	0.07	0.07	0.06	0.89
Heckman, $R_x \sim X$ and Y, $R_y \sim X$	NARFCS Shift full data	0.00	0.05	0.05	0.07	0.99	-0.00	0.05	0.05	0.05	0.94	0.00	0.05	0.05	0.06	0.96
Heckman, $R_x \sim X$ and Y, $R_y \sim X$	NARFCS Int. full data	0.00	0.04	0.04	0.07	1.00	0.00	0.04	0.04	0.05	0.99	0.01	0.03	0.04	0.06	1.00
Heckman, $R_x \sim X$ and Y, $R_y \sim X$	NARFCS Int. X fully obs.	0.00	0.03	0.03	0.06	1.00	0.00	0.03	0.03	0.05	0.99	0.00	0.03	0.03	0.05	1.00
Heckman, $R_x \sim X$ and Y, $R_y \sim X$	MI RI	-0.08	0.13	0.15	0.29	1.00	-0.04	0.05	0.06	0.07	0.97	-0.03	0.07	0.08	0.14	1.00
Heckman, $R_x \sim X$ and Y, $R_y \sim X$	MI RI X fully obs.	-0.04	0.11	0.12	0.26	1.00	0.01	0.05	0.05	0.06	0.98	-0.02	0.07	0.07	0.13	1.00
Heckman, $R_x \sim X$ and Y, $R_y \sim Y$	All cases	-0.00	0.03	0.03	0.03	0.94	-0.00	0.03	0.03	0.03	0.95	-0.00	0.03	0.03	0.03	0.95
Heckman, $R_x \sim X$ and Y, $R_y \sim Y$	CC	0.24	0.06	0.25	0.06	0.02	-0.01	0.05	0.05	0.05	0.95	0.10	0.06	0.12	0.06	0.59
Heckman, $R_x \sim X$ and Y, $R_y \sim Y$	MI MAR	0.17	0.06	0.18	0.06	0.21	-0.01	0.05	0.05	0.05	0.94	0.08	0.05	0.10	0.06	0.68
Heckman, $R_x \sim X$ and Y, $R_y \sim Y$	ML HE X cc	0.03	0.09	0.09	0.09	0.94	-0.03	0.05	0.06	0.05	0.90	0.01	0.06	0.07	0.06	0.94
Heckman, $R_x \sim X$ and Y, $R_y \sim Y$	ML HE X excl.	0.03	0.09	0.10	0.09	0.94	-0.03	0.05	0.06	0.05	0.91	0.00	0.07	0.07	0.07	0.95
Heckman, $R_x \sim X$ and Y, $R_y \sim Y$	ML HE X fully obs.	-0.00	0.09	0.09	0.09	0.94	-0.00	0.04	0.04	0.04	0.95	-0.00	0.06	0.06	0.06	0.96
Heckman, $R_x \sim X$ and Y, $R_y \sim Y$	UI X cc	0.12	0.06	0.14	0.06	0.48	-0.02	0.05	0.06	0.05	0.93	0.05	0.06	0.08	0.06	0.84
Heckman, $R_x \sim X$ and Y, $R_y \sim Y$	UI X fully obs.	0.00	0.05	0.05	0.05	0.96	-0.00	0.04	0.04	0.05	0.96	-0.00	0.05	0.05	0.05	0.96
Heckman, $R_x \sim X$ and Y, $R_y \sim Y$	MI HE X cc	0.04	0.09	0.10	0.09	0.94	-0.03	0.05	0.06	0.05	0.92	0.02	0.06	0.07	0.07	0.94
Heckman, $R_x \sim X$ and Y, $R_y \sim Y$	MI HE	0.00	0.09	0.09	0.10	0.96	-0.02	0.05	0.06	0.05	0.92	-0.00	0.06	0.06	0.06	0.95
Heckman, $R_x \sim X$ and Y, $R_y \sim Y$	MI HE X fully obs.	-0.00	0.09	0.09	0.09	0.93	-0.00	0.05	0.05	0.05	0.94	-0.00	0.06	0.06	0.06	0.94
Heckman, $R_x \sim X$ and Y, $R_y \sim Y$	Stacking full data	0.00	0.06	0.06	0.07	0.98	-0.02	0.05	0.05	0.06	0.96	0.01	0.06	0.06	0.06	0.96
Heckman, $R_x \sim X$ and Y, $R_y \sim Y$	Stacking X MAR	-0.08	0.05	0.10	0.07	0.84	-0.02	0.05	0.06	0.06	0.94	-0.04	0.05	0.07	0.06	0.93
Heckman, $R_x \sim X$ and Y, $R_y \sim Y$	Stacking X fully obs.	-0.01	0.05	0.05	0.06	0.99	-0.00	0.05	0.05	0.05	0.97	-0.01	0.05	0.05	0.06	0.97
Heckman, $R_x \sim X$ and Y, $R_y \sim Y$	NARFCS Shift SP	0.31	0.10	0.32	0.07	0.04	-0.00	0.05	0.05	0.05	0.95	0.14	0.07	0.16	0.06	0.32
Heckman, $R_x \sim X$ and Y, $R_y \sim Y$	NARFCS Shift full data	-0.00	0.05	0.05	0.07	1.00	-0.00	0.05	0.05	0.05	0.95	-0.00	0.05	0.05	0.06	0.96
Heckman, $R_x \sim X$ and Y, $R_y \sim Y$	NARFCS Int. full data	-0.00	0.04	0.04	0.07	1.00	-0.00	0.04	0.04	0.05	0.98	-0.00	0.04	0.04	0.06	1.00
Heckman, $R_x \sim X$ and Y, $R_y \sim Y$	NARFCS Int. X fully obs.	0.00	0.03	0.03	0.06	1.00	-0.00	0.03	0.03	0.05	0.99	-0.00	0.03	0.03	0.05	1.00
Heckman, $R_x \sim X$ and Y, $R_y \sim Y$	MI RI	0.11	0.13	0.17	0.28	1.00	-0.05	0.05	0.07	0.08	0.97	0.05	0.08	0.09	0.14	0.99
Heckman, $R_x \sim X$ and Y, $R_y \sim Y$	MI RI X fully obs.	0.24	0.10	0.26	0.25	0.96	-0.00	0.04	0.04	0.05	0.95	0.11	0.07	0.13	0.12	0.95
Heckman, $R_x \sim X$ and Y, $R_y \sim X$ and Y	All cases	0.00	0.03	0.03	0.03	0.96	-0.00	0.03	0.03	0.03	0.95	-0.00	0.03	0.03	0.03	0.96
Heckman, $R_x \sim X$ and Y, $R_y \sim X$ and Y	CC	0.25	0.06	0.25	0.06	0.02	-0.03	0.05	0.06	0.05	0.89	0.10	0.06	0.12	0.06	0.58
Heckman, $R_x \sim X$ and Y, $R_y \sim X$ and Y	MI MAR	0.24	0.06	0.25	0.06	0.04	-0.03	0.05	0.06	0.05	0.92	0.11	0.05	0.12	0.05	0.47
Heckman, $R_x \sim X$ and Y, $R_y \sim X$ and Y	ML HE X cc	0.04	0.09	0.09	0.09	0.94	-0.01	0.05	0.05	0.05	0.95	0.01	0.07	0.07	0.06	0.95
Heckman, $R_x \sim X$ and Y, $R_y \sim X$ and Y	ML HE X excl.	0.03	0.10	0.10	0.09	0.93	-0.06	0.05	0.08	0.05	0.79	0.00	0.07	0.07	0.07	0.94
Heckman, $R_x \sim X$ and Y, $R_y \sim X$ and Y	ML HE X fully obs.	0.00	0.09	0.09	0.08	0.94	-0.00	0.05	0.05	0.05	0.94	0.00	0.06	0.06	0.06	0.95
Heckman, $R_x \sim X$ and Y, $R_y \sim X$ and Y	UI X cc	0.13	0.06	0.15	0.06	0.45	-0.02	0.05	0.05	0.05	0.94	0.05	0.06	0.08	0.06	0.83
Heckman, $R_x \sim X$ and Y, $R_y \sim X$ and Y	UI X fully obs.	0.01	0.05	0.05	0.05	0.95	-0.00	0.04	0.04	0.05	0.96	0.00	0.05	0.05	0.05	0.96
Heckman, $R_x \sim X$ and Y, $R_y \sim X$ and Y	MI HE X cc	0.04	0.09	0.09	0.09	0.93	-0.01	0.05	0.05	0.05	0.96	0.01	0.07	0.07	0.07	0.95
Heckman, $R_x \sim X$ and Y, $R_y \sim X$ and Y	MI HE	-0.01	0.09	0.09	0.10	0.95	-0.02	0.05	0.05	0.05	0.93	-0.00	0.06	0.06	0.06	0.94
Heckman, $R_x \sim X$ and Y, $R_y \sim X$ and Y	MI HE X fully obs.	0.00	0.09	0.09	0.09	0.94	-0.00	0.05	0.05	0.05	0.94	0.00	0.06	0.06	0.06	0.95
Heckman, $R_x \sim X$ and Y, $R_y \sim X$ and Y	Stacking full data	0.02	0.06	0.06	0.07	0.97	-0.03	0.05	0.06	0.06	0.96	0.02	0.06	0.06	0.07	0.97
Heckman, $R_x \sim X$ and Y, $R_y \sim X$ and Y	Stacking X MAR	-0.03	0.05	0.06	0.07	0.98	-0.02	0.05	0.05	0.06	0.96	-0.01	0.05	0.05	0.06	0.97
Heckman, $R_x \sim X$ and Y, $R_y \sim X$ and Y	Stacking X fully obs.	-0.02	0.05	0.05	0.07	0.99	0.00	0.05	0.05	0.05	0.96	-0.01	0.05	0.05	0.06	0.97
Heckman, $R_x \sim X$ and Y, $R_y \sim X$ and Y	NARFCS Shift SP	0.32	0.10	0.34	0.07	0.03	-0.06	0.05	0.07	0.05	0.80	0.15	0.07	0.16	0.06	0.28
Heckman, $R_x \sim X$ and Y, $R_y \sim X$ and Y	NARFCS Shift full data	0.00	0.05	0.05	0.07	0.99	0.00	0.05	0.05	0.05	0.96	0.00	0.05	0.05	0.06	0.96
Heckman, $R_x \sim X$ and Y, $R_y \sim X$ and Y	NARFCS Int. full data	0.00	0.03	0.03	0.07	1.00	0.00	0.04	0.04	0.05	0.99	0.00	0.03	0.03	0.06	0.99
Heckman, $R_x \sim X$ and Y, $R_y \sim X$ and Y	NARFCS Int. X fully obs.	-0.00	0.03	0.03	0.06	1.00	-0.00	0.03	0.03	0.05	0.99	-0.00	0.03	0.03	0.05	1.00
Heckman, $R_x \sim X$ and Y, $R_y \sim X$ and Y	MI RI	0.25	0.12	0.28	0.27	0.97	-0.05	0.05	0.07	0.06	0.91	0.12	0.07	0.14	0.13	0.97
Heckman, $R_x \sim X$ and Y, $R_y \sim X$ and Y	MI RI X fully obs.	0.29	0.11	0.31	0.25	0.91	-0.04	0.05	0.06	0.06	0.93	0.13	0.06	0.15	0.12	0.91

Table S11: (4)Performance of the methods in the simulation study with non-monotone missing. DGM: data-generating mechanism; empse: empirical standard error; rmse: root mean square error; modse: model standard error; cov: empirical coverage of the 95% confidence intervals; see the description of abbreviations in Table S2.

		Intercept					β_x					β_z				
DGM	Method	bias	empse	rmse	modse	cov	bias	empse	rmse	modse	cov	bias	empse	rmse	modse	cov
Logistic, R_x=0.5X	All cases	-0.00	0.03	0.03	0.03	0.95	-0.00	0.03	0.03	0.03	0.94	0.00	0.03	0.03	0.03	0.96
Logistic, R_x=0.5X	CC	-0.00	0.07	0.07	0.07	0.94	-0.00	0.06	0.06	0.06	0.95	-0.00	0.06	0.06	0.06	0.96
Logistic, R_x=0.5X	MI MAR	-0.10	0.06	0.11	0.06	0.62	-0.01	0.05	0.05	0.06	0.95	-0.03	0.05	0.06	0.05	0.89
Logistic, R_x=0.5X	ML HE X cc	-0.01	0.16	0.16	0.15	0.92	-0.00	0.06	0.06	0.06	0.95	-0.01	0.08	0.08	0.08	0.95
Logistic, R_x=0.5X	ML HE X excl.	-0.01	0.14	0.14	0.14	0.94	-0.00	0.06	0.06	0.06	0.95	-0.01	0.08	0.08	0.08	0.95
Logistic, R_x=0.5X	ML HE X fully obs.	-0.01	0.12	0.12	0.12	0.95	-0.00	0.04	0.04	0.04	0.96	-0.00	0.06	0.06	0.06	0.95
Logistic, R_x=0.5X	UI X cc	-0.18	0.07	0.19	0.07	0.24	-0.01	0.06	0.06	0.06	0.95	-0.06	0.06	0.09	0.06	0.84
Logistic, R_x=0.5X	UI X fully obs.	-0.21	0.05	0.22	0.05	0.01	-0.00	0.04	0.04	0.05	0.96	-0.08	0.05	0.09	0.05	0.65
Logistic, R_x=0.5X	MI HE X cc	-0.01	0.16	0.16	0.15	0.91	-0.00	0.06	0.06	0.06	0.95	-0.00	0.08	0.08	0.08	0.94
Logistic, R_x=0.5X	MI HE	-0.02	0.12	0.13	0.13	0.95	-0.00	0.05	0.05	0.06	0.95	-0.01	0.07	0.07	0.07	0.95
Logistic, R_x=0.5X	MI HE X fully obs.	-0.01	0.12	0.12	0.12	0.94	-0.00	0.04	0.04	0.05	0.95	-0.00	0.07	0.07	0.06	0.93
Logistic, R_x=0.5X	Stacking full data	-0.02	0.05	0.05	0.06	0.97	-0.01	0.05	0.05	0.05	0.93	-0.01	0.06	0.06	0.06	0.94
Logistic, R_x=0.5X	Stacking X MAR	-0.10	0.04	0.11	0.06	0.62	-0.01	0.05	0.05	0.06	0.95	-0.03	0.05	0.06	0.05	0.89
Logistic, R_x=0.5X	Stacking X fully obs.	0.00	0.04	0.04	0.05	1.00	-0.00	0.04	0.04	0.05	0.95	-0.00	0.05	0.05	0.05	0.96
Logistic, R_x=0.5X	NARFCS Shift SP	-0.13	0.10	0.17	0.06	0.43	-0.02	0.06	0.06	0.06	0.94	-0.05	0.06	0.08	0.05	0.81
Logistic, R_x=0.5X	NARFCS Shift full data	0.00	0.04	0.04	0.06	0.99	-0.01	0.05	0.05	0.05	0.94	0.00	0.05	0.05	0.05	0.96
Logistic, R_x=0.5X	NARFCS Int. full data	-0.00	0.03	0.03	0.06	1.00	-0.00	0.04	0.04	0.05	0.98	0.00	0.03	0.03	0.05	1.00
Logistic, R_x=0.5X	NARFCS Int. X fully obs.	-0.00	0.03	0.03	0.05	1.00	-0.00	0.03	0.03	0.05	0.99	0.00	0.03	0.03	0.05	1.00
Logistic, R_x=0.5X	MI RI	-0.17	0.08	0.19	0.27	1.00	-0.04	0.05	0.06	0.07	0.95	-0.06	0.06	0.08	0.11	0.99
Logistic, R_x=0.5X	MI RI X fully obs.	-0.06	0.08	0.10	0.25	1.00	-0.00	0.04	0.04	0.05	0.96	-0.02	0.05	0.06	0.10	1.00
Logistic, R_x=0.5Y	All cases	0.00	0.03	0.03	0.03	0.96	0.00	0.03	0.03	0.03	0.94	-0.00	0.03	0.03	0.03	0.96
Logistic, R_x=0.5Y	CC	0.17	0.06	0.18	0.06	0.23	-0.02	0.05	0.06	0.06	0.94	0.04	0.06	0.07	0.06	0.89
Logistic, R_x=0.5Y	MI MAR	-0.02	0.06	0.06	0.06	0.94	-0.01	0.05	0.05	0.06	0.95	-0.01	0.05	0.05	0.05	0.95
Logistic, R_x=0.5Y	ML HE X cc	0.01	0.15	0.15	0.15	0.95	-0.02	0.06	0.06	0.06	0.94	-0.01	0.08	0.08	0.08	0.94
Logistic, R_x=0.5Y	ML HE X excl.	0.03	0.14	0.14	0.13	0.94	-0.02	0.06	0.06	0.06	0.94	-0.01	0.08	0.08	0.07	0.94
Logistic, R_x=0.5Y	ML HE X fully obs.	0.00	0.11	0.11	0.12	0.96	0.00	0.05	0.05	0.05	0.95	0.00	0.06	0.06	0.06	0.95
Logistic, R_x=0.5Y	UI X cc	-0.01	0.06	0.06	0.07	0.97	-0.02	0.06	0.06	0.06	0.94	-0.02	0.06	0.06	0.06	0.94
Logistic, R_x=0.5Y	UI X fully obs.	-0.21	0.05	0.22	0.05	0.01	0.00	0.05	0.05	0.05	0.95	-0.08	0.05	0.09	0.05	0.68
Logistic, R_x=0.5Y	MI HE X cc	0.01	0.15	0.15	0.15	0.92	-0.02	0.06	0.06	0.06	0.94	-0.01	0.08	0.08	0.08	0.94
Logistic, R_x=0.5Y	MI HE	0.01	0.12	0.12	0.13	0.95	-0.00	0.06	0.06	0.06	0.95	0.00	0.06	0.06	0.07	0.95
Logistic, R_x=0.5Y	MI HE X fully obs.	0.00	0.12	0.12	0.12	0.95	0.00	0.05	0.05	0.05	0.94	0.00	0.06	0.06	0.06	0.96
Logistic, R_x=0.5Y	Stacking full data	0.01	0.05	0.05	0.06	0.98	-0.01	0.05	0.05	0.05	0.94	0.01	0.05	0.05	0.05	0.94
Logistic, R_x=0.5Y	Stacking X MAR	-0.02	0.05	0.05	0.06	0.97	-0.01	0.05	0.05	0.06	0.95	-0.01	0.05	0.05	0.05	0.96
Logistic, R_x=0.5Y	Stacking X fully obs.	-0.00	0.04	0.04	0.05	0.99	0.00	0.05	0.05	0.05	0.94	-0.00	0.04	0.04	0.05	0.96
Logistic, R_x=0.5Y	NARFCS Shift SP	0.12	0.10	0.15	0.06	0.48	-0.00	0.06	0.06	0.05	0.94	0.04	0.06	0.07	0.05	0.83
Logistic, R_x=0.5Y	NARFCS Shift full data	0.00	0.04	0.04	0.06	0.99	-0.00	0.05	0.05	0.05	0.94	-0.00	0.05	0.05	0.05	0.96
Logistic, R_x=0.5Y	NARFCS Int. full data	0.00	0.03	0.03	0.06	1.00	-0.00	0.04	0.04	0.05	0.98	-0.00	0.03	0.03	0.05	1.00
Logistic, R_x=0.5Y	NARFCS Int. X fully obs.	-0.00	0.03	0.03	0.05	1.00	0.00	0.03	0.03	0.05	0.99	-0.00	0.03	0.03	0.05	1.00
Logistic, R_x=0.5Y	MI RI	-0.10	0.09	0.13	0.27	1.00	-0.05	0.05	0.07	0.07	0.95	-0.04	0.05	0.07	0.11	1.00
Logistic, R_x=0.5Y	MI RI X fully obs.	-0.06	0.08	0.10	0.25	1.00	0.00	0.05	0.05	0.05	0.95	-0.02	0.05	0.06	0.10	1.00
Logistic, R_x=0.5$X+0.5Y$	All cases	0.00	0.03	0.03	0.03	0.95	0.00	0.03	0.03	0.03	0.95	0.00	0.03	0.03	0.03	0.95
Logistic, R_x=0.5$X+0.5Y$	CC	0.17	0.07	0.18	0.07	0.25	-0.05	0.06	0.08	0.06	0.86	0.04	0.06	0.07	0.06	0.89
Logistic, R_x=0.5$X+0.5Y$	MI MAR	-0.11	0.06	0.12	0.06	0.55	-0.04	0.06	0.07	0.06	0.89	-0.03	0.05	0.06	0.05	0.88
Logistic, R_x=0.5$X+0.5Y$	ML HE X cc	0.02	0.15	0.15	0.15	0.94	-0.06	0.06	0.08	0.06	0.84	-0.02	0.08	0.08	0.08	0.93
Logistic, R_x=0.5$X+0.5Y$	ML HE X excl.	0.03	0.14	0.14	0.14	0.93	-0.06	0.06	0.08	0.06	0.84	-0.01	0.08	0.08	0.08	0.93
Logistic, R_x=0.5$X+0.5Y$	ML HE X fully obs.	-0.00	0.12	0.12	0.12	0.95	0.00	0.04	0.04	0.05	0.95	-0.00	0.06	0.06	0.06	0.95
Logistic, R_x=0.5$X+0.5Y$	UI X cc	-0.01	0.07	0.07	0.07	0.96	-0.06	0.06	0.08	0.06	0.84	-0.02	0.06	0.07	0.06	0.94
Logistic, R_x=0.5$X+0.5Y$	UI X fully obs.	-0.22	0.05	0.22	0.05	0.01	0.00	0.05	0.05	0.05	0.96	-0.08	0.05	0.09	0.05	0.63
Logistic, R_x=0.5$X+0.5Y$	MI HE X cc	0.02	0.15	0.15	0.15	0.92	-0.06	0.06	0.08	0.06	0.85	-0.02	0.08	0.08	0.08	0.93
Logistic, R_x=0.5$X+0.5Y$	MI HE	-0.01	0.12	0.12	0.13	0.94	-0.02	0.06	0.07	0.06	0.93	0.01	0.07	0.07	0.07	0.93
Logistic, R_x=0.5$X+0.5Y$	MI HE X fully obs.	-0.00	0.12	0.12	0.12	0.92	0.00	0.05	0.05	0.05	0.95	-0.00	0.06	0.06	0.06	0.94
Logistic, R_x=0.5$X+0.5Y$	Stacking full data	-0.01	0.05	0.05	0.06	0.99	-0.03	0.06	0.06	0.06	0.90	0.01	0.05	0.06	0.06	0.94
Logistic, R_x=0.5$X+0.5Y$	Stacking X MAR	-0.11	0.05	0.12	0.06	0.56	-0.04	0.06	0.07	0.06	0.89	-0.03	0.05	0.06	0.05	0.90
Logistic, R_x=0.5$X+0.5Y$	Stacking X fully obs.	-0.00	0.04	0.04	0.05	0.99	0.00	0.05	0.05	0.05	0.94	-0.00	0.05	0.05	0.05	0.94
Logistic, R_x=0.5$X+0.5Y$	NARFCS Shift SP	-0.01	0.09	0.10	0.06	0.78	-0.05	0.06	0.08	0.06	0.89	0.01	0.06	0.06	0.05	0.91
Logistic, R_x=0.5$X+0.5Y$	NARFCS Shift full data	-0.00	0.04	0.04	0.06	0.99	0.00	0.05	0.05	0.05	0.94	-0.00	0.05	0.05	0.05	0.94
Logistic, R_x=0.5$X+0.5Y$	NARFCS Int. full data	0.00	0.04	0.04	0.06	0.99	-0.00	0.04	0.04	0.05	0.98	0.00	0.03	0.03	0.05	1.00
Logistic, R_x=0.5$X+0.5Y$	NARFCS Int. X fully obs.	0.00	0.03	0.03	0.05	1.00	0.00	0.03	0.03	0.05	0.99	0.00	0.03	0.03	0.05	0.99
Logistic, R_x=0.5$X+0.5Y$	MI RI	-0.18	0.09	0.20	0.27	1.00	-0.08	0.06	0.10	0.09	0.90	-0.06	0.06	0.08	0.11	0.99
Logistic, R_x=0.5$X+0.5Y$	MI RI X fully obs.	-0.06	0.08	0.10	0.25	1.00	0.00	0.05	0.05	0.05	0.95	-0.02	0.05	0.06	0.10	1.00

Table S12: (5)Performance of the methods in the simulation study with non-monotone missing. DGM: data-generating mechanism; empse: empirical standard error; rmse: root mean square error; modse: model standard error; cov: empirical coverage of the 95% confidence intervals; see the description of abbreviations in Table S2.

		Intercept					β_x					β_z				
DGM	Method	bias	empse	rmse	modse	cov	bias	empse	rmse	modse	cov	bias	empse	rmse	modse	cov
Logistic, R_y=0.5X	All cases	0.00	0.03	0.03	0.03	0.95	0.00	0.03	0.03	0.03	0.95	-0.00	0.03	0.03	0.03	0.95
Logistic, R_y=0.5X	CC	0.00	0.07	0.07	0.07	0.93	0.00	0.06	0.06	0.06	0.95	-0.00	0.06	0.06	0.06	0.96
Logistic, R_y=0.5X	MI MAR	0.06	0.06	0.09	0.06	0.80	-0.01	0.06	0.06	0.06	0.94	0.03	0.05	0.06	0.05	0.92
Logistic, R_y=0.5X	ML HE X cc	0.00	0.16	0.16	0.16	0.94	-0.00	0.06	0.06	0.06	0.96	-0.00	0.08	0.08	0.08	0.95
Logistic, R_y=0.5X	ML HE X excl.	0.00	0.14	0.14	0.14	0.95	0.00	0.06	0.06	0.06	0.95	-0.00	0.08	0.08	0.08	0.95
Logistic, R_y=0.5X	ML HE X fully obs.	0.00	0.13	0.13	0.12	0.93	0.00	0.05	0.05	0.05	0.95	-0.00	0.06	0.06	0.06	0.95
Logistic, R_y=0.5X	UI X cc	-0.18	0.07	0.19	0.07	0.27	0.04	0.06	0.07	0.06	0.91	-0.06	0.06	0.09	0.06	0.84
Logistic, R_y=0.5X	UI X fully obs.	-0.21	0.05	0.22	0.05	0.01	0.04	0.05	0.06	0.05	0.88	-0.08	0.05	0.09	0.05	0.67
Logistic, R_y=0.5X	MI HE X cc	0.00	0.16	0.16	0.16	0.92	-0.00	0.06	0.06	0.07	0.97	-0.00	0.08	0.08	0.08	0.95
Logistic, R_y=0.5X	MI HE	-0.00	0.13	0.13	0.13	0.93	-0.00	0.06	0.06	0.06	0.94	-0.00	0.07	0.07	0.07	0.95
Logistic, R_y=0.5X	MI HE X fully obs.	0.00	0.13	0.13	0.12	0.92	-0.00	0.05	0.05	0.05	0.94	-0.00	0.06	0.06	0.06	0.94
Logistic, R_y=0.5X	Stacking full data	0.01	0.05	0.05	0.06	0.98	-0.01	0.05	0.05	0.05	0.93	0.00	0.06	0.06	0.06	0.94
Logistic, R_y=0.5X	Stacking X MAR	0.06	0.05	0.08	0.06	0.86	-0.01	0.05	0.06	0.06	0.94	0.03	0.05	0.06	0.05	0.91
Logistic, R_y=0.5X	Stacking X fully obs.	0.00	0.04	0.04	0.05	0.99	-0.00	0.05	0.05	0.05	0.95	-0.00	0.05	0.05	0.05	0.96
Logistic, R_y=0.5X	NARFCS Shift SP	-0.04	0.11	0.12	0.06	0.69	-0.01	0.06	0.06	0.05	0.92	-0.02	0.06	0.06	0.05	0.88
Logistic, R_y=0.5X	NARFCS Shift full data	0.00	0.05	0.05	0.06	0.99	-0.00	0.05	0.06	0.05	0.94	-0.00	0.05	0.05	0.05	0.95
Logistic, R_y=0.5X	NARFCS Int. full data	0.00	0.04	0.04	0.06	1.00	-0.00	0.04	0.04	0.05	0.98	0.00	0.03	0.03	0.05	0.99
Logistic, R_y=0.5X	NARFCS Int. X fully obs.	0.00	0.03	0.03	0.05	1.00	0.00	0.03	0.03	0.05	0.99	-0.00	0.03	0.03	0.05	1.00
Logistic, R_y=0.5X	MI RI	0.02	0.09	0.09	0.27	1.00	-0.03	0.05	0.06	0.07	0.97	0.01	0.06	0.06	0.11	1.00
Logistic, R_y=0.5X	MI RI X fully obs.	-0.03	0.08	0.08	0.25	1.00	0.00	0.05	0.05	0.06	0.99	-0.01	0.05	0.05	0.10	1.00
Logistic, R_y=0.5Y	All cases	-0.00	0.03	0.03	0.03	0.95	-0.00	0.03	0.03	0.03	0.95	-0.00	0.03	0.03	0.03	0.93
Logistic, R_y=0.5Y	CC	0.17	0.06	0.18	0.06	0.24	-0.02	0.06	0.06	0.06	0.94	0.04	0.06	0.08	0.06	0.88
Logistic, R_y=0.5Y	MI MAR	0.26	0.06	0.27	0.06	0.00	-0.03	0.05	0.06	0.05	0.92	0.08	0.05	0.09	0.05	0.65
Logistic, R_y=0.5Y	ML HE X cc	-0.01	0.15	0.15	0.15	0.93	-0.00	0.06	0.06	0.06	0.95	0.00	0.07	0.07	0.07	0.94
Logistic, R_y=0.5Y	ML HE X excl.	0.01	0.14	0.14	0.14	0.94	-0.02	0.06	0.06	0.06	0.93	-0.01	0.08	0.08	0.07	0.94
Logistic, R_y=0.5Y	ML HE X fully obs.	0.01	0.12	0.12	0.11	0.95	-0.00	0.04	0.04	0.05	0.95	0.00	0.06	0.06	0.05	0.94
Logistic, R_y=0.5Y	UI X cc	-0.01	0.06	0.07	0.07	0.95	-0.00	0.06	0.06	0.06	0.96	-0.00	0.07	0.07	0.06	0.94
Logistic, R_y=0.5Y	UI X fully obs.	-0.01	0.05	0.05	0.05	0.95	-0.00	0.04	0.04	0.05	0.96	-0.00	0.05	0.05	0.05	0.95
Logistic, R_y=0.5Y	MI HE X cc	-0.00	0.15	0.15	0.15	0.92	-0.00	0.06	0.06	0.06	0.96	0.00	0.08	0.08	0.07	0.94
Logistic, R_y=0.5Y	MI HE	0.01	0.12	0.12	0.12	0.94	-0.01	0.05	0.06	0.06	0.95	0.00	0.06	0.06	0.06	0.94
Logistic, R_y=0.5Y	MI HE X fully obs.	0.01	0.12	0.12	0.12	0.93	-0.00	0.05	0.05	0.05	0.95	0.00	0.06	0.06	0.06	0.94
Logistic, R_y=0.5Y	Stacking full data	0.04	0.05	0.06	0.06	0.95	-0.01	0.05	0.06	0.05	0.94	0.02	0.06	0.06	0.05	0.93
Logistic, R_y=0.5Y	Stacking X MAR	0.04	0.05	0.06	0.06	0.94	-0.01	0.05	0.05	0.06	0.96	0.02	0.05	0.05	0.05	0.94
Logistic, R_y=0.5Y	Stacking X fully obs.	0.01	0.04	0.04	0.06	0.99	-0.00	0.04	0.04	0.05	0.96	0.00	0.05	0.05	0.05	0.94
Logistic, R_y=0.5Y	NARFCS Shift SP	0.35	0.10	0.36	0.06	0.01	-0.04	0.05	0.07	0.05	0.90	0.10	0.06	0.11	0.05	0.52
Logistic, R_y=0.5Y	NARFCS Shift full data	-0.00	0.04	0.04	0.06	0.99	-0.00	0.05	0.05	0.05	0.95	0.00	0.05	0.05	0.05	0.93
Logistic, R_y=0.5Y	NARFCS Int. full data	-0.00	0.03	0.03	0.06	1.00	-0.00	0.04	0.04	0.05	0.98	-0.00	0.04	0.04	0.05	0.99
Logistic, R_y=0.5Y	NARFCS Int. X fully obs.	-0.00	0.03	0.03	0.05	1.00	-0.00	0.03	0.03	0.05	0.99	-0.00	0.03	0.03	0.05	0.99
Logistic, R_y=0.5Y	MI RI	0.21	0.08	0.23	0.26	1.00	-0.05	0.05	0.07	0.07	0.92	0.06	0.05	0.08	0.09	0.96
Logistic, R_y=0.5Y	MI RI X fully obs.	0.20	0.07	0.21	0.24	1.00	-0.02	0.04	0.05	0.05	0.95	0.05	0.05	0.07	0.08	0.96
Logistic, R_y=0.5$X+0.5Y$	All cases	0.00	0.03	0.03	0.03	0.96	-0.00	0.03	0.03	0.03	0.95	-0.00	0.03	0.03	0.03	0.95
Logistic, R_y=0.5$X+0.5Y$	CC	0.17	0.06	0.18	0.07	0.26	-0.06	0.06	0.08	0.06	0.84	0.04	0.06	0.07	0.06	0.90
Logistic, R_y=0.5$X+0.5Y$	MI MAR	0.31	0.06	0.31	0.05	0.00	-0.08	0.05	0.10	0.06	0.72	0.09	0.05	0.11	0.05	0.53
Logistic, R_y=0.5$X+0.5Y$	ML HE X cc	-0.01	0.15	0.15	0.15	0.93	-0.00	0.07	0.07	0.07	0.95	-0.00	0.07	0.07	0.07	0.96
Logistic, R_y=0.5$X+0.5Y$	ML HE X excl.	0.01	0.14	0.14	0.14	0.96	-0.06	0.06	0.09	0.06	0.80	-0.01	0.07	0.07	0.07	0.96
Logistic, R_y=0.5$X+0.5Y$	ML HE X fully obs.	-0.00	0.11	0.11	0.12	0.95	-0.00	0.05	0.05	0.05	0.96	-0.00	0.05	0.05	0.05	0.95
Logistic, R_y=0.5$X+0.5Y$	UI X cc	-0.01	0.07	0.07	0.07	0.96	-0.00	0.06	0.06	0.06	0.95	-0.00	0.06	0.06	0.06	0.96
Logistic, R_y=0.5$X+0.5Y$	UI X fully obs.	-0.01	0.05	0.05	0.05	0.95	0.00	0.04	0.04	0.05	0.96	-0.01	0.05	0.05	0.05	0.95
Logistic, R_y=0.5$X+0.5Y$	MI HE X cc	-0.01	0.15	0.15	0.15	0.93	-0.00	0.07	0.07	0.07	0.95	-0.00	0.07	0.07	0.07	0.96
Logistic, R_y=0.5$X+0.5Y$	MI HE	-0.00	0.12	0.12	0.13	0.95	-0.01	0.06	0.06	0.06	0.96	-0.00	0.06	0.06	0.06	0.96
Logistic, R_y=0.5$X+0.5Y$	MI HE X fully obs.	0.00	0.12	0.12	0.12	0.94	-0.00	0.05	0.05	0.06	0.95	-0.00	0.06	0.06	0.06	0.94
Logistic, R_y=0.5$X+0.5Y$	Stacking full data	0.05	0.05	0.07	0.06	0.93	-0.04	0.06	0.07	0.06	0.91	0.02	0.05	0.06	0.06	0.93
Logistic, R_y=0.5$X+0.5Y$	Stacking X MAR	0.08	0.04	0.09	0.06	0.76	-0.04	0.05	0.07	0.06	0.92	0.03	0.05	0.06	0.05	0.91
Logistic, R_y=0.5$X+0.5Y$	Stacking X fully obs.	0.01	0.04	0.04	0.06	0.99	-0.00	0.05	0.05	0.05	0.96	0.00	0.05	0.05	0.05	0.95
Logistic, R_y=0.5$X+0.5Y$	NARFCS Shift SP	0.31	0.10	0.33	0.06	0.03	-0.09	0.06	0.11	0.06	0.59	0.08	0.06	0.10	0.05	0.63
Logistic, R_y=0.5$X+0.5Y$	NARFCS Shift full data	0.00	0.04	0.04	0.06	0.99	-0.01	0.06	0.06	0.06	0.94	-0.00	0.05	0.05	0.05	0.94
Logistic, R_y=0.5$X+0.5Y$	NARFCS Int. full data	0.00	0.04	0.04	0.06	1.00	-0.00	0.04	0.05	0.06	0.97	-0.00	0.04	0.04	0.05	0.99
Logistic, R_y=0.5$X+0.5Y$	NARFCS Int. X fully obs.	-0.00	0.03	0.03	0.05	0.99	-0.00	0.03	0.03	0.05	0.99	-0.00	0.03	0.03	0.05	0.99
Logistic, R_y=0.5$X+0.5Y$	MI RI	0.28	0.08	0.29	0.26	0.96	-0.10	0.05	0.11	0.09	0.90	0.08	0.05	0.10	0.09	0.92
Logistic, R_y=0.5$X+0.5Y$	MI RI X fully obs.	0.23	0.08	0.24	0.24	0.98	-0.06	0.05	0.08	0.08	0.95	0.06	0.05	0.08	0.08	0.96

Table S13: (6)Performance of the methods in the simulation study with non-monotone missing. DGM: data-generating mechanism; empse: empirical standard error; rmse: root mean square error; modse: model standard error; cov: empirical coverage of the 95% confidence intervals; see the description of abbreviations in Table S2.

		Intercept					β_x					β_z				
DGM	Method	bias	empse	rmse	modse	cov	bias	empse	rmse	modse	cov	bias	empse	rmse	modse	cov
Logistic, $R_x=0.5X$, $R_y=0.5X$	All cases	0.00	0.03	0.03	0.03	0.94	0.00	0.03	0.03	0.03	0.95	0.00	0.03	0.03	0.03	0.95
Logistic, $R_x=0.5X$, $R_y=0.5X$	CC	0.00	0.07	0.07	0.07	0.94	-0.00	0.06	0.06	0.06	0.95	0.00	0.06	0.06	0.06	0.96
Logistic, $R_x=0.5X$, $R_y=0.5X$	MI MAR	-0.04	0.06	0.07	0.06	0.89	-0.00	0.05	0.05	0.06	0.96	-0.00	0.05	0.05	0.05	0.95
Logistic, $R_x=0.5X$, $R_y=0.5X$	ML HE X cc	0.01	0.16	0.16	0.16	0.93	-0.00	0.06	0.06	0.06	0.95	0.00	0.08	0.08	0.08	0.95
Logistic, $R_x=0.5X$, $R_y=0.5X$	ML HE X excl.	0.01	0.14	0.14	0.14	0.93	-0.00	0.06	0.06	0.06	0.95	0.00	0.08	0.08	0.08	0.95
Logistic, $R_x=0.5X$, $R_y=0.5X$	ML HE X fully obs.	0.01	0.12	0.12	0.12	0.94	-0.00	0.05	0.05	0.05	0.95	0.00	0.06	0.06	0.06	0.95
Logistic, $R_x=0.5X$, $R_y=0.5X$	UI X cc	-0.18	0.07	0.19	0.07	0.28	0.03	0.06	0.06	0.06	0.94	-0.06	0.06	0.09	0.06	0.85
Logistic, $R_x=0.5X$, $R_y=0.5X$	UI X fully obs.	-0.22	0.05	0.22	0.05	0.01	0.04	0.05	0.06	0.05	0.90	-0.08	0.05	0.09	0.05	0.69
Logistic, $R_x=0.5X$, $R_y=0.5X$	MI HE X cc	0.01	0.16	0.16	0.16	0.91	-0.00	0.06	0.06	0.06	0.95	0.00	0.08	0.08	0.08	0.94
Logistic, $R_x=0.5X$, $R_y=0.5X$	MI HE	-0.00	0.13	0.13	0.13	0.94	-0.01	0.06	0.06	0.06	0.94	0.00	0.07	0.07	0.07	0.96
Logistic, $R_x=0.5X$, $R_y=0.5X$	MI HE X fully obs.	0.01	0.13	0.13	0.12	0.94	-0.00	0.05	0.05	0.05	0.96	0.00	0.06	0.06	0.06	0.94
Logistic, $R_x=0.5X$, $R_y=0.5X$	Stacking full data	-0.01	0.05	0.05	0.06	0.98	-0.00	0.05	0.05	0.06	0.96	0.00	0.05	0.05	0.06	0.96
Logistic, $R_x=0.5X$, $R_y=0.5X$	Stacking X MAR	-0.04	0.05	0.06	0.06	0.95	-0.00	0.05	0.05	0.06	0.95	-0.00	0.05	0.05	0.05	0.95
Logistic, $R_x=0.5X$, $R_y=0.5X$	Stacking X fully obs.	0.00	0.04	0.04	0.05	0.99	-0.00	0.04	0.04	0.05	0.95	0.00	0.05	0.05	0.05	0.96
Logistic, $R_x=0.5X$, $R_y=0.5X$	NARFCS Shift SP	-0.17	0.11	0.20	0.06	0.34	-0.03	0.06	0.06	0.06	0.91	-0.06	0.06	0.08	0.05	0.77
Logistic, $R_x=0.5X$, $R_y=0.5X$	NARFCS Shift full data	0.00	0.04	0.04	0.06	0.99	-0.00	0.05	0.05	0.05	0.95	0.00	0.05	0.05	0.05	0.95
Logistic, $R_x=0.5X$, $R_y=0.5X$	NARFCS Int. full data	0.00	0.04	0.04	0.06	1.00	-0.00	0.04	0.04	0.05	0.98	0.00	0.03	0.03	0.05	1.00
Logistic, $R_x=0.5X$, $R_y=0.5X$	NARFCS Int. X fully obs.	0.00	0.03	0.03	0.05	1.00	0.00	0.03	0.03	0.05	0.99	0.00	0.03	0.03	0.05	1.00
Logistic, $R_x=0.5X$, $R_y=0.5X$	MI RI	-0.08	0.09	0.12	0.28	1.00	-0.02	0.05	0.06	0.07	0.97	-0.02	0.05	0.06	0.11	1.00
Logistic, $R_x=0.5X$, $R_y=0.5X$	MI RI X fully obs.	-0.03	0.08	0.09	0.25	1.00	0.00	0.05	0.05	0.06	0.98	-0.01	0.05	0.05	0.10	1.00
Logistic, $R_x=0.5X$, $R_y=0.5Y$	All cases	0.00	0.03	0.03	0.03	0.94	0.00	0.03	0.03	0.03	0.95	-0.00	0.03	0.03	0.03	0.94
Logistic, $R_x=0.5X$, $R_y=0.5Y$	CC	0.17	0.06	0.18	0.07	0.25	-0.01	0.06	0.06	0.06	0.95	0.04	0.06	0.07	0.06	0.90
Logistic, $R_x=0.5X$, $R_y=0.5Y$	MI MAR	0.16	0.06	0.17	0.06	0.18	-0.01	0.05	0.06	0.05	0.94	0.04	0.05	0.06	0.05	0.88
Logistic, $R_x=0.5X$, $R_y=0.5Y$	ML HE X cc	0.00	0.15	0.15	0.15	0.92	-0.00	0.06	0.06	0.06	0.95	-0.00	0.07	0.07	0.07	0.96
Logistic, $R_x=0.5X$, $R_y=0.5Y$	ML HE X excl.	0.02	0.14	0.14	0.14	0.94	-0.02	0.06	0.06	0.06	0.94	-0.01	0.07	0.07	0.07	0.96
Logistic, $R_x=0.5X$, $R_y=0.5Y$	ML HE X fully obs.	0.01	0.11	0.11	0.12	0.95	-0.00	0.05	0.05	0.05	0.95	-0.00	0.05	0.05	0.05	0.95
Logistic, $R_x=0.5X$, $R_y=0.5Y$	UI X cc	-0.01	0.07	0.07	0.07	0.96	-0.00	0.06	0.06	0.06	0.95	-0.00	0.06	0.06	0.06	0.96
Logistic, $R_x=0.5X$, $R_y=0.5Y$	UI X fully obs.	-0.01	0.05	0.05	0.05	0.95	0.00	0.04	0.04	0.05	0.96	-0.01	0.05	0.05	0.05	0.96
Logistic, $R_x=0.5X$, $R_y=0.5Y$	MI HE X cc	0.00	0.15	0.15	0.15	0.92	-0.00	0.06	0.06	0.06	0.95	-0.00	0.07	0.07	0.07	0.96
Logistic, $R_x=0.5X$, $R_y=0.5Y$	MI HE	0.00	0.12	0.12	0.13	0.95	-0.01	0.05	0.06	0.06	0.95	-0.01	0.06	0.06	0.06	0.96
Logistic, $R_x=0.5X$, $R_y=0.5Y$	MI HE X fully obs.	0.01	0.11	0.11	0.12	0.94	-0.00	0.05	0.05	0.05	0.95	-0.00	0.05	0.05	0.06	0.95
Logistic, $R_x=0.5X$, $R_y=0.5Y$	Stacking full data	0.01	0.05	0.05	0.06	0.97	-0.02	0.05	0.06	0.06	0.94	0.01	0.05	0.05	0.06	0.95
Logistic, $R_x=0.5X$, $R_y=0.5Y$	Stacking X MAR	-0.07	0.05	0.08	0.06	0.87	-0.00	0.05	0.05	0.06	0.96	-0.03	0.05	0.06	0.05	0.94
Logistic, $R_x=0.5X$, $R_y=0.5Y$	Stacking X fully obs.	0.01	0.04	0.04	0.06	0.99	-0.00	0.04	0.04	0.05	0.96	-0.00	0.05	0.05	0.05	0.96
Logistic, $R_x=0.5X$, $R_y=0.5Y$	NARFCS Shift SP	0.22	0.10	0.24	0.06	0.17	-0.03	0.05	0.06	0.05	0.92	0.05	0.06	0.07	0.05	0.81
Logistic, $R_x=0.5X$, $R_y=0.5Y$	NARFCS Shift full data	0.00	0.04	0.04	0.06	0.98	-0.00	0.05	0.05	0.05	0.95	-0.00	0.05	0.05	0.05	0.95
Logistic, $R_x=0.5X$, $R_y=0.5Y$	NARFCS Int. full data	0.00	0.03	0.03	0.06	1.00	-0.00	0.04	0.04	0.05	0.98	-0.00	0.04	0.04	0.05	0.99
Logistic, $R_x=0.5X$, $R_y=0.5Y$	NARFCS Int. X fully obs.	0.00	0.03	0.03	0.05	0.99	0.00	0.03	0.03	0.04	0.99	-0.00	0.03	0.03	0.05	0.99
Logistic, $R_x=0.5X$, $R_y=0.5Y$	MI RI	0.12	0.08	0.14	0.26	1.00	-0.04	0.05	0.07	0.06	0.93	0.03	0.05	0.06	0.09	0.99
Logistic, $R_x=0.5X$, $R_y=0.5Y$	MI RI X fully obs.	0.20	0.07	0.21	0.24	1.00	-0.02	0.04	0.05	0.05	0.96	0.05	0.05	0.07	0.08	0.98
Logistic, $R_x=0.5X$, $R_y=0.5X+0.5Y$	All cases	-0.00	0.03	0.03	0.03	0.96	0.00	0.03	0.03	0.03	0.95	0.00	0.03	0.03	0.03	0.94
Logistic, $R_x=0.5X$, $R_y=0.5X+0.5Y$	CC	0.18	0.07	0.19	0.07	0.25	-0.04	0.06	0.07	0.06	0.89	0.04	0.06	0.07	0.06	0.88
Logistic, $R_x=0.5X$, $R_y=0.5X+0.5Y$	MI MAR	0.22	0.06	0.22	0.06	0.04	-0.05	0.05	0.08	0.06	0.86	0.06	0.05	0.08	0.05	0.73
Logistic, $R_x=0.5X$, $R_y=0.5X+0.5Y$	ML HE X cc	-0.01	0.15	0.15	0.15	0.94	0.00	0.07	0.07	0.07	0.96	0.00	0.07	0.07	0.07	0.95
Logistic, $R_x=0.5X$, $R_y=0.5X+0.5Y$	ML HE X excl.	0.02	0.14	0.14	0.14	0.95	-0.06	0.06	0.08	0.06	0.84	-0.00	0.07	0.07	0.07	0.95
Logistic, $R_x=0.5X$, $R_y=0.5X+0.5Y$	ML HE X fully obs.	0.01	0.12	0.12	0.12	0.95	-0.00	0.05	0.05	0.05	0.97	0.00	0.06	0.06	0.05	0.94
Logistic, $R_x=0.5X$, $R_y=0.5X+0.5Y$	UI X cc	-0.01	0.07	0.07	0.07	0.96	0.00	0.06	0.06	0.06	0.96	0.00	0.06	0.06	0.06	0.95
Logistic, $R_x=0.5X$, $R_y=0.5X+0.5Y$	UI X fully obs.	-0.01	0.05	0.05	0.05	0.95	0.01	0.04	0.04	0.05	0.96	-0.00	0.05	0.05	0.05	0.95
Logistic, $R_x=0.5X$, $R_y=0.5X+0.5Y$	MI HE X cc	-0.00	0.15	0.15	0.15	0.94	0.00	0.07	0.07	0.07	0.96	0.00	0.07	0.07	0.07	0.95
Logistic, $R_x=0.5X$, $R_y=0.5X+0.5Y$	MI HE	-0.00	0.13	0.13	0.13	0.94	-0.02	0.06	0.06	0.06	0.94	-0.01	0.06	0.06	0.06	0.94
Logistic, $R_x=0.5X$, $R_y=0.5X+0.5Y$	MI HE X fully obs.	0.01	0.12	0.12	0.12	0.92	-0.00	0.05	0.05	0.06	0.96	0.00	0.06	0.06	0.06	0.94
Logistic, $R_x=0.5X$, $R_y=0.5X+0.5Y$	Stacking full data	0.03	0.05	0.06	0.07	0.97	-0.03	0.06	0.06	0.06	0.93	0.02	0.05	0.06	0.06	0.94
Logistic, $R_x=0.5X$, $R_y=0.5X+0.5Y$	Stacking X MAR	-0.02	0.04	0.05	0.06	0.98	-0.02	0.05	0.06	0.06	0.94	0.00	0.05	0.05	0.05	0.95
Logistic, $R_x=0.5X$, $R_y=0.5X+0.5Y$	Stacking X fully obs.	0.01	0.04	0.04	0.06	0.99	0.00	0.04	0.04	0.05	0.97	0.00	0.05	0.05	0.05	0.95
Logistic, $R_x=0.5X$, $R_y=0.5X+0.5Y$	NARFCS Shift SP	0.21	0.10	0.23	0.06	0.21	-0.09	0.06	0.11	0.06	0.59	0.04	0.06	0.07	0.05	0.82
Logistic, $R_x=0.5X$, $R_y=0.5X+0.5Y$	NARFCS Shift full data	0.00	0.04	0.04	0.06	0.99	0.00	0.05	0.05	0.06	0.95	0.00	0.05	0.05	0.05	0.94
Logistic, $R_x=0.5X$, $R_y=0.5X+0.5Y$	NARFCS Int. full data	0.00	0.03	0.03	0.06	1.00	0.00	0.04	0.04	0.06	0.98	0.00	0.03	0.04	0.05	0.99
Logistic, $R_x=0.5X$, $R_y=0.5X+0.5Y$	NARFCS Int. X fully obs.	-0.00	0.03	0.03	0.05	1.00	0.00	0.03	0.03	0.05	1.00	0.00	0.03	0.03	0.05	0.99
Logistic, $R_x=0.5X$, $R_y=0.5X+0.5Y$	MI RI	0.19	0.08	0.21	0.27	1.00	-0.07	0.05	0.09	0.08	0.92	0.06	0.05	0.08	0.09	0.98
Logistic, $R_x=0.5X$, $R_y=0.5X+0.5Y$	MI RI X fully obs.	0.23	0.08	0.24	0.24	0.99	-0.06	0.05	0.07	0.08	0.97	0.06	0.05	0.08	0.08	0.94

Table S14: (7)Performance of the methods in the simulation study with non-monotone missing. DGM: data-generating mechanism; empse: empirical standard error; rmse: root mean square error; modse: model standard error; cov: empirical coverage of the 95% confidence intervals; see the description of abbreviations in Table S2.

		Intercept					β_x					β_z				
DGM	Method	bias	empse	rmse	modse	cov	bias	empse	rmse	modse	cov	bias	empse	rmse	modse	cov
Logistic, R_x=0.5Y, R_y=0.5X	All cases	-0.00	0.03	0.03	0.03	0.95	0.00	0.03	0.03	0.03	0.96	-0.00	0.03	0.03	0.03	0.95
Logistic, R_x=0.5Y, R_y=0.5X	CC	0.17	0.07	0.19	0.07	0.24	-0.01	0.06	0.06	0.06	0.94	0.04	0.06	0.08	0.06	0.89
Logistic, R_x=0.5Y, R_y=0.5X	MI MAR	0.04	0.06	0.07	0.06	0.89	0.01	0.05	0.05	0.05	0.94	0.02	0.05	0.06	0.05	0.93
Logistic, R_x=0.5Y, R_y=0.5X	ML HE X cc	0.00	0.15	0.15	0.15	0.94	0.02	0.06	0.06	0.06	0.94	-0.01	0.08	0.08	0.08	0.95
Logistic, R_x=0.5Y, R_y=0.5X	ML HE X excl.	0.02	0.14	0.14	0.14	0.95	-0.02	0.06	0.06	0.06	0.94	-0.01	0.07	0.07	0.07	0.96
Logistic, R_x=0.5Y, R_y=0.5X	ML HE X fully obs.	0.01	0.12	0.12	0.12	0.95	0.00	0.05	0.05	0.05	0.95	0.00	0.06	0.06	0.06	0.96
Logistic, R_x=0.5Y, R_y=0.5X	UI X cc	-0.01	0.07	0.07	0.07	0.95	0.02	0.06	0.06	0.06	0.93	-0.02	0.06	0.06	0.06	0.95
Logistic, R_x=0.5Y, R_y=0.5X	UI X fully obs.	-0.22	0.05	0.22	0.05	0.01	0.04	0.05	0.06	0.05	0.87	-0.08	0.05	0.09	0.05	0.66
Logistic, R_x=0.5Y, R_y=0.5X	MI HE X cc	0.00	0.15	0.15	0.15	0.93	0.02	0.06	0.07	0.06	0.93	-0.01	0.08	0.08	0.08	0.95
Logistic, R_x=0.5Y, R_y=0.5X	MI HE	0.01	0.12	0.12	0.13	0.95	-0.00	0.06	0.06	0.06	0.95	0.00	0.06	0.06	0.07	0.95
Logistic, R_x=0.5Y, R_y=0.5X	MI HE X fully obs.	0.01	0.12	0.12	0.12	0.93	0.00	0.05	0.05	0.05	0.95	0.00	0.06	0.06	0.06	0.94
Logistic, R_x=0.5Y, R_y=0.5X	Stacking full data	0.02	0.05	0.05	0.06	0.96	-0.01	0.05	0.05	0.06	0.95	0.02	0.05	0.06	0.06	0.95
Logistic, R_x=0.5Y, R_y=0.5X	Stacking X MAR	0.04	0.05	0.06	0.06	0.93	0.01	0.05	0.05	0.05	0.94	0.02	0.05	0.05	0.05	0.94
Logistic, R_x=0.5Y, R_y=0.5X	Stacking X fully obs.	-0.00	0.04	0.04	0.05	0.99	0.00	0.05	0.05	0.05	0.96	-0.00	0.05	0.05	0.05	0.96
Logistic, R_x=0.5Y, R_y=0.5X	NARFCS Shift SP	0.07	0.10	0.12	0.06	0.65	-0.03	0.06	0.07	0.06	0.91	0.03	0.06	0.07	0.05	0.86
Logistic, R_x=0.5Y, R_y=0.5X	NARFCS Shift full data	0.00	0.04	0.04	0.06	0.99	0.00	0.05	0.05	0.06	0.95	0.00	0.05	0.05	0.05	0.95
Logistic, R_x=0.5Y, R_y=0.5X	NARFCS Int. full data	0.00	0.03	0.03	0.06	1.00	-0.00	0.04	0.04	0.06	0.99	-0.00	0.04	0.04	0.05	1.00
Logistic, R_x=0.5Y, R_y=0.5X	NARFCS Int. X fully obs.	-0.00	0.03	0.03	0.05	1.00	0.00	0.03	0.03	0.05	0.99	-0.00	0.03	0.03	0.05	1.00
Logistic, R_x=0.5Y, R_y=0.5X	MI RI	-0.02	0.08	0.09	0.27	1.00	-0.02	0.05	0.06	0.07	0.97	-0.00	0.05	0.05	0.11	1.00
Logistic, R_x=0.5Y, R_y=0.5X	MI RI X fully obs.	-0.03	0.08	0.09	0.25	1.00	0.01	0.05	0.05	0.06	0.98	-0.01	0.05	0.05	0.10	1.00
Logistic, R_x=0.5Y, R_y=0.5Y	All cases	-0.00	0.03	0.03	0.03	0.94	-0.00	0.03	0.03	0.03	0.95	-0.00	0.03	0.03	0.03	0.95
Logistic, R_x=0.5Y, R_y=0.5Y	CC	0.33	0.06	0.34	0.06	0.00	-0.03	0.06	0.06	0.06	0.92	0.09	0.06	0.10	0.06	0.69
Logistic, R_x=0.5Y, R_y=0.5Y	MI MAR	0.25	0.06	0.25	0.05	0.01	-0.02	0.05	0.06	0.05	0.93	0.07	0.05	0.08	0.05	0.71
Logistic, R_x=0.5Y, R_y=0.5Y	ML HE X cc	0.02	0.13	0.13	0.13	0.95	-0.00	0.06	0.06	0.06	0.94	0.01	0.07	0.07	0.07	0.94
Logistic, R_x=0.5Y, R_y=0.5Y	ML HE X excl.	0.04	0.13	0.13	0.13	0.93	-0.04	0.06	0.07	0.05	0.89	0.00	0.07	0.07	0.07	0.95
Logistic, R_x=0.5Y, R_y=0.5Y	ML HE X fully obs.	0.00	0.11	0.11	0.11	0.94	0.00	0.05	0.05	0.05	0.95	-0.00	0.05	0.05	0.05	0.95
Logistic, R_x=0.5Y, R_y=0.5Y	UI X cc	0.16	0.06	0.17	0.06	0.26	-0.02	0.06	0.06	0.06	0.94	0.04	0.06	0.07	0.06	0.89
Logistic, R_x=0.5Y, R_y=0.5Y	UI X fully obs.	-0.01	0.05	0.05	0.05	0.94	0.00	0.04	0.04	0.05	0.96	-0.01	0.05	0.05	0.05	0.95
Logistic, R_x=0.5Y, R_y=0.5Y	MI HE X cc	0.03	0.12	0.13	0.13	0.94	-0.00	0.06	0.06	0.06	0.95	0.01	0.07	0.07	0.07	0.95
Logistic, R_x=0.5Y, R_y=0.5Y	MI HE	0.01	0.12	0.12	0.12	0.94	-0.01	0.06	0.06	0.06	0.93	-0.00	0.06	0.06	0.06	0.94
Logistic, R_x=0.5Y, R_y=0.5Y	MI HE X fully obs.	0.00	0.11	0.11	0.11	0.93	0.00	0.05	0.05	0.05	0.95	-0.00	0.06	0.06	0.06	0.95
Logistic, R_x=0.5Y, R_y=0.5Y	Stacking full data	0.05	0.05	0.07	0.06	0.91	-0.02	0.05	0.06	0.06	0.94	0.02	0.05	0.06	0.05	0.92
Logistic, R_x=0.5Y, R_y=0.5Y	Stacking X MAR	0.02	0.05	0.05	0.06	0.97	-0.01	0.05	0.05	0.06	0.96	0.00	0.05	0.05	0.05	0.96
Logistic, R_x=0.5Y, R_y=0.5Y	Stacking X fully obs.	0.01	0.04	0.04	0.06	0.99	-0.00	0.05	0.05	0.05	0.97	-0.00	0.05	0.05	0.05	0.97
Logistic, R_x=0.5Y, R_y=0.5Y	NARFCS Shift SP	0.44	0.09	0.45	0.06	0.00	-0.04	0.06	0.07	0.05	0.87	0.12	0.05	0.13	0.05	0.33
Logistic, R_x=0.5Y, R_y=0.5Y	NARFCS Shift full data	0.00	0.04	0.04	0.06	0.99	-0.00	0.06	0.06	0.05	0.94	-0.00	0.05	0.05	0.05	0.94
Logistic, R_x=0.5Y, R_y=0.5Y	NARFCS Int. full data	0.00	0.03	0.03	0.06	1.00	-0.00	0.04	0.04	0.05	0.98	-0.00	0.03	0.03	0.05	0.99
Logistic, R_x=0.5Y, R_y=0.5Y	NARFCS Int. X fully obs.	-0.00	0.03	0.03	0.05	1.00	-0.00	0.03	0.03	0.05	0.99	-0.00	0.03	0.03	0.05	0.99
Logistic, R_x=0.5Y, R_y=0.5Y	MI RI	0.18	0.08	0.20	0.26	1.00	-0.05	0.05	0.07	0.06	0.93	0.05	0.05	0.07	0.09	0.97
Logistic, R_x=0.5Y, R_y=0.5Y	MI RI X fully obs.	0.19	0.07	0.21	0.24	1.00	-0.02	0.04	0.05	0.05	0.95	0.05	0.05	0.07	0.08	0.97
Logistic, R_x=0.5Y, R_y=0.5$X+0.5Y$	All cases	-0.00	0.03	0.03	0.03	0.95	0.00	0.03	0.03	0.03	0.95	0.00	0.03	0.03	0.03	0.96
Logistic, R_x=0.5Y, R_y=0.5$X+0.5Y$	CC	0.33	0.06	0.34	0.06	0.00	-0.06	0.06	0.08	0.06	0.83	0.08	0.06	0.10	0.06	0.70
Logistic, R_x=0.5Y, R_y=0.5$X+0.5Y$	MI MAR	0.28	0.05	0.29	0.05	0.00	-0.04	0.05	0.07	0.05	0.86	0.08	0.05	0.09	0.05	0.63
Logistic, R_x=0.5Y, R_y=0.5$X+0.5Y$	ML HE X cc	0.03	0.13	0.14	0.13	0.94	0.03	0.07	0.07	0.07	0.93	0.01	0.06	0.06	0.07	0.95
Logistic, R_x=0.5Y, R_y=0.5$X+0.5Y$	ML HE X excl.	0.03	0.14	0.14	0.14	0.93	-0.07	0.06	0.09	0.06	0.73	-0.00	0.07	0.07	0.07	0.95
Logistic, R_x=0.5Y, R_y=0.5$X+0.5Y$	ML HE X fully obs.	0.01	0.12	0.12	0.12	0.94	-0.00	0.05	0.05	0.05	0.96	0.00	0.05	0.05	0.05	0.97
Logistic, R_x=0.5Y, R_y=0.5$X+0.5Y$	UI X cc	0.16	0.06	0.17	0.06	0.30	-0.01	0.06	0.06	0.06	0.95	0.04	0.06	0.07	0.06	0.90
Logistic, R_x=0.5Y, R_y=0.5$X+0.5Y$	UI X fully obs.	-0.01	0.05	0.05	0.05	0.95	0.00	0.05	0.05	0.05	0.97	-0.00	0.04	0.04	0.05	0.97
Logistic, R_x=0.5Y, R_y=0.5$X+0.5Y$	MI HE X cc	0.03	0.13	0.13	0.13	0.94	0.03	0.07	0.07	0.07	0.93	0.01	0.06	0.07	0.07	0.96
Logistic, R_x=0.5Y, R_y=0.5$X+0.5Y$	MI HE	0.01	0.13	0.13	0.13	0.94	-0.01	0.06	0.06	0.06	0.95	0.00	0.06	0.06	0.06	0.96
Logistic, R_x=0.5Y, R_y=0.5$X+0.5Y$	MI HE X fully obs.	0.01	0.12	0.12	0.12	0.94	-0.00	0.05	0.05	0.06	0.96	0.00	0.05	0.05	0.06	0.96
Logistic, R_x=0.5Y, R_y=0.5$X+0.5Y$	Stacking full data	0.06	0.05	0.08	0.06	0.90	-0.04	0.06	0.07	0.06	0.89	0.03	0.05	0.05	0.06	0.94
Logistic, R_x=0.5Y, R_y=0.5$X+0.5Y$	Stacking X MAR	0.06	0.04	0.07	0.06	0.88	-0.01	0.05	0.06	0.06	0.95	0.02	0.05	0.05	0.05	0.95
Logistic, R_x=0.5Y, R_y=0.5$X+0.5Y$	Stacking X fully obs.	0.01	0.04	0.04	0.06	0.99	-0.00	0.05	0.05	0.05	0.97	0.00	0.04	0.04	0.05	0.96
Logistic, R_x=0.5Y, R_y=0.5$X+0.5Y$	NARFCS Shift SP	0.41	0.09	0.42	0.06	0.00	-0.12	0.06	0.14	0.06	0.44	0.10	0.05	0.12	0.05	0.44
Logistic, R_x=0.5Y, R_y=0.5$X+0.5Y$	NARFCS Shift full data	-0.00	0.04	0.04	0.06	0.99	0.00	0.06	0.06	0.06	0.95	-0.00	0.05	0.05	0.05	0.96
Logistic, R_x=0.5Y, R_y=0.5$X+0.5Y$	NARFCS Int. full data	-0.00	0.03	0.03	0.06	1.00	0.00	0.04	0.04	0.06	0.99	-0.00	0.03	0.03	0.05	1.00
Logistic, R_x=0.5Y, R_y=0.5$X+0.5Y$	NARFCS Int. X fully obs.	-0.00	0.03	0.03	0.05	1.00	0.00	0.03	0.03	0.05	0.99	0.00	0.03	0.03	0.05	0.99
Logistic, R_x=0.5Y, R_y=0.5$X+0.5Y$	MI RI	0.24	0.07	0.26	0.26	0.99	-0.06	0.05	0.08	0.07	0.92	0.07	0.05	0.08	0.08	0.96
Logistic, R_x=0.5Y, R_y=0.5$X+0.5Y$	MI RI X fully obs.	0.23	0.07	0.24	0.24	0.99	-0.06	0.05	0.08	0.08	0.96	0.06	0.05	0.07	0.08	0.96

Table S15: (8)Performance of the methods in the simulation study with non-monotone missing. DGM: data-generating mechanism; empse: empirical standard error; rmse: root mean square error; modse: model standard error; cov: empirical coverage of the 95% confidence intervals; see the description of abbreviations in Table S2.

		Intercept					β_x					β_z				
DGM	Method	bias	empse	rmse	modse	cov	bias	empse	rmse	modse	cov	bias	empse	rmse	modse	cov
Logistic, $R_x=0.5X+0.5Y$, $R_y=0.5X$	All cases	0.00	0.03	0.03	0.03	0.94	-0.00	0.03	0.03	0.03	0.95	0.00	0.03	0.03	0.03	0.95
Logistic, $R_x=0.5X+0.5Y$, $R_y=0.5X$	CC	0.17	0.07	0.19	0.07	0.27	-0.04	0.06	0.07	0.06	0.89	0.04	0.06	0.07	0.06	0.89
Logistic, $R_x=0.5X+0.5Y$, $R_y=0.5X$	MI MAR	-0.04	0.06	0.07	0.06	0.87	-0.01	0.06	0.06	0.06	0.94	-0.00	0.05	0.05	0.05	0.95
Logistic, $R_x=0.5X+0.5Y$, $R_y=0.5X$	ML HE X cc	0.01	0.15	0.15	0.15	0.93	-0.02	0.06	0.06	0.06	0.94	-0.01	0.08	0.08	0.08	0.94
Logistic, $R_x=0.5X+0.5Y$, $R_y=0.5X$	ML HE X excl.	0.03	0.14	0.14	0.14	0.93	-0.06	0.06	0.08	0.06	0.85	-0.01	0.08	0.08	0.07	0.95
Logistic, $R_x=0.5X+0.5Y$, $R_y=0.5X$	ML HE X fully obs.	-0.00	0.12	0.12	0.12	0.96	0.00	0.05	0.05	0.05	0.95	-0.00	0.06	0.06	0.06	0.95
Logistic, $R_x=0.5X+0.5Y$, $R_y=0.5X$	UI X cc	-0.01	0.07	0.07	0.07	0.95	-0.02	0.06	0.06	0.06	0.95	-0.02	0.06	0.06	0.06	0.95
Logistic, $R_x=0.5X+0.5Y$, $R_y=0.5X$	UI X fully obs.	-0.21	0.05	0.22	0.05	0.01	0.04	0.05	0.06	0.05	0.87	-0.08	0.05	0.09	0.05	0.67
Logistic, $R_x=0.5X+0.5Y$, $R_y=0.5X$	MI HE X cc	0.01	0.15	0.15	0.15	0.92	-0.02	0.06	0.07	0.06	0.94	-0.01	0.08	0.08	0.08	0.94
Logistic, $R_x=0.5X+0.5Y$, $R_y=0.5X$	MI HE	-0.01	0.12	0.12	0.13	0.95	-0.04	0.06	0.07	0.06	0.93	0.00	0.07	0.07	0.07	0.95
Logistic, $R_x=0.5X+0.5Y$, $R_y=0.5X$	MI HE X fully obs.	-0.00	0.12	0.12	0.12	0.94	0.00	0.05	0.05	0.05	0.94	-0.00	0.06	0.06	0.06	0.95
Logistic, $R_x=0.5X+0.5Y$, $R_y=0.5X$	Stacking full data	0.01	0.05	0.05	0.07	0.98	-0.03	0.06	0.06	0.06	0.95	0.01	0.05	0.05	0.06	0.96
Logistic, $R_x=0.5X+0.5Y$, $R_y=0.5X$	Stacking X MAR	-0.05	0.04	0.06	0.06	0.93	-0.01	0.06	0.06	0.06	0.94	-0.01	0.05	0.05	0.05	0.96
Logistic, $R_x=0.5X+0.5Y$, $R_y=0.5X$	Stacking X fully obs.	0.00	0.04	0.04	0.05	0.99	0.00	0.05	0.05	0.05	0.95	-0.00	0.05	0.05	0.05	0.96
Logistic, $R_x=0.5X+0.5Y$, $R_y=0.5X$	NARFCS Shift SP	-0.03	0.10	0.11	0.06	0.76	-0.09	0.07	0.11	0.06	0.65	0.00	0.06	0.06	0.05	0.91
Logistic, $R_x=0.5X+0.5Y$, $R_y=0.5X$	NARFCS Shift full data	0.00	0.04	0.04	0.06	0.99	0.00	0.05	0.05	0.05	0.94	0.00	0.05	0.05	0.05	0.96
Logistic, $R_x=0.5X+0.5Y$, $R_y=0.5X$	NARFCS Int. full data	0.00	0.03	0.03	0.06	1.00	0.00	0.04	0.04	0.05	0.99	0.00	0.03	0.03	0.05	0.99
Logistic, $R_x=0.5X+0.5Y$, $R_y=0.5X$	NARFCS Int. X fully obs.	0.00	0.03	0.03	0.05	1.00	0.00	0.03	0.03	0.05	0.99	-0.00	0.03	0.03	0.05	0.99
Logistic, $R_x=0.5X+0.5Y$, $R_y=0.5X$	MI RI	-0.09	0.09	0.13	0.27	1.00	-0.04	0.05	0.07	0.07	0.96	-0.02	0.05	0.06	0.11	1.00
Logistic, $R_x=0.5X+0.5Y$, $R_y=0.5X$	MI RI X fully obs.	-0.03	0.08	0.08	0.25	1.00	0.01	0.05	0.05	0.06	0.99	-0.01	0.05	0.05	0.10	1.00
Logistic, $R_x=0.5X+0.5Y$, $R_y=0.5Y$	All cases	0.00	0.03	0.03	0.03	0.95	-0.00	0.03	0.03	0.03	0.95	-0.00	0.03	0.03	0.03	0.96
Logistic, $R_x=0.5X+0.5Y$, $R_y=0.5Y$	CC	0.34	0.06	0.34	0.06	0.00	-0.06	0.06	0.08	0.06	0.81	0.08	0.06	0.10	0.06	0.69
Logistic, $R_x=0.5X+0.5Y$, $R_y=0.5Y$	MI MAR	0.16	0.05	0.17	0.06	0.17	-0.04	0.06	0.07	0.06	0.88	0.05	0.05	0.07	0.05	0.84
Logistic, $R_x=0.5X+0.5Y$, $R_y=0.5Y$	ML HE X cc	0.04	0.13	0.14	0.13	0.94	-0.05	0.06	0.07	0.06	0.87	0.01	0.07	0.07	0.07	0.96
Logistic, $R_x=0.5X+0.5Y$, $R_y=0.5Y$	ML HE X excl.	0.05	0.14	0.14	0.13	0.93	-0.08	0.06	0.10	0.06	0.72	-0.00	0.07	0.07	0.07	0.95
Logistic, $R_x=0.5X+0.5Y$, $R_y=0.5Y$	ML HE X fully obs.	0.01	0.12	0.12	0.11	0.94	-0.00	0.05	0.05	0.05	0.95	0.00	0.06	0.06	0.05	0.95
Logistic, $R_x=0.5X+0.5Y$, $R_y=0.5Y$	UI X cc	0.16	0.06	0.17	0.06	0.27	-0.05	0.06	0.08	0.06	0.86	0.04	0.06	0.07	0.06	0.92
Logistic, $R_x=0.5X+0.5Y$, $R_y=0.5Y$	UI X fully obs.	-0.01	0.05	0.05	0.05	0.95	-0.00	0.04	0.04	0.05	0.96	-0.00	0.05	0.05	0.05	0.96
Logistic, $R_x=0.5X+0.5Y$, $R_y=0.5Y$	MI HE X cc	0.05	0.13	0.14	0.13	0.94	-0.05	0.06	0.08	0.06	0.89	0.01	0.07	0.07	0.07	0.96
Logistic, $R_x=0.5X+0.5Y$, $R_y=0.5Y$	MI HE	0.00	0.12	0.12	0.13	0.93	-0.05	0.06	0.07	0.06	0.86	0.00	0.06	0.06	0.06	0.94
Logistic, $R_x=0.5X+0.5Y$, $R_y=0.5Y$	MI HE X fully obs.	0.01	0.12	0.12	0.12	0.93	-0.00	0.05	0.05	0.05	0.95	0.00	0.06	0.06	0.06	0.95
Logistic, $R_x=0.5X+0.5Y$, $R_y=0.5Y$	Stacking full data	0.04	0.05	0.06	0.07	0.95	-0.05	0.05	0.07	0.06	0.88	0.02	0.05	0.05	0.06	0.95
Logistic, $R_x=0.5X+0.5Y$, $R_y=0.5Y$	Stacking X MAR	-0.07	0.05	0.08	0.06	0.86	-0.04	0.06	0.07	0.06	0.92	-0.02	0.05	0.05	0.05	0.95
Logistic, $R_x=0.5X+0.5Y$, $R_y=0.5Y$	Stacking X fully obs.	0.01	0.04	0.04	0.06	0.99	-0.00	0.05	0.05	0.05	0.95	0.00	0.05	0.05	0.05	0.97
Logistic, $R_x=0.5X+0.5Y$, $R_y=0.5Y$	NARFCS Shift SP	0.33	0.09	0.34	0.06	0.01	-0.07	0.06	0.10	0.06	0.74	0.09	0.05	0.11	0.05	0.55
Logistic, $R_x=0.5X+0.5Y$, $R_y=0.5Y$	NARFCS Shift full data	0.00	0.04	0.04	0.06	0.99	-0.00	0.05	0.05	0.05	0.95	0.00	0.05	0.05	0.05	0.96
Logistic, $R_x=0.5X+0.5Y$, $R_y=0.5Y$	NARFCS Int. full data	0.00	0.03	0.03	0.06	1.00	-0.00	0.04	0.04	0.05	0.98	-0.00	0.03	0.03	0.05	1.00
Logistic, $R_x=0.5X+0.5Y$, $R_y=0.5Y$	NARFCS Int. X fully obs.	0.00	0.03	0.03	0.05	1.00	-0.00	0.03	0.03	0.05	0.99	-0.00	0.03	0.03	0.05	0.99
Logistic, $R_x=0.5X+0.5Y$, $R_y=0.5Y$	MI RI	0.10	0.08	0.13	0.26	1.00	-0.07	0.06	0.09	0.07	0.88	0.03	0.05	0.06	0.09	0.99
Logistic, $R_x=0.5X+0.5Y$, $R_y=0.5Y$	MI RI X fully obs.	0.19	0.07	0.21	0.24	0.99	-0.02	0.05	0.05	0.05	0.95	0.05	0.05	0.07	0.08	0.97
Logistic, $R_x=0.5X+0.5Y$, $R_y=0.5X+0.5Y$	All cases	-0.00	0.03	0.03	0.03	0.96	0.00	0.03	0.03	0.03	0.94	0.00	0.03	0.03	0.03	0.95
Logistic, $R_x=0.5X+0.5Y$, $R_y=0.5X+0.5Y$	CC	0.34	0.06	0.34	0.06	0.00	-0.08	0.06	0.10	0.06	0.72	0.08	0.06	0.10	0.06	0.69
Logistic, $R_x=0.5X+0.5Y$, $R_y=0.5X+0.5Y$	MI MAR	0.21	0.06	0.22	0.06	0.05	-0.05	0.05	0.07	0.06	0.85	0.06	0.05	0.08	0.05	0.77
Logistic, $R_x=0.5X+0.5Y$, $R_y=0.5X+0.5Y$	ML HE X cc	0.03	0.13	0.13	0.13	0.95	-0.01	0.06	0.06	0.07	0.95	0.01	0.06	0.06	0.07	0.95
Logistic, $R_x=0.5X+0.5Y$, $R_y=0.5X+0.5Y$	ML HE X excl.	0.04	0.14	0.14	0.14	0.93	-0.11	0.06	0.12	0.06	0.54	0.00	0.07	0.07	0.07	0.95
Logistic, $R_x=0.5X+0.5Y$, $R_y=0.5X+0.5Y$	ML HE X fully obs.	0.01	0.11	0.11	0.12	0.95	0.00	0.05	0.05	0.05	0.96	0.00	0.05	0.05	0.05	0.95
Logistic, $R_x=0.5X+0.5Y$, $R_y=0.5X+0.5Y$	UI X cc	0.16	0.07	0.18	0.07	0.30	-0.04	0.06	0.07	0.06	0.90	0.04	0.06	0.07	0.06	0.90
Logistic, $R_x=0.5X+0.5Y$, $R_y=0.5X+0.5Y$	UI X fully obs.	-0.01	0.05	0.05	0.05	0.95	0.01	0.05	0.05	0.05	0.96	-0.00	0.05	0.05	0.05	0.96
Logistic, $R_x=0.5X+0.5Y$, $R_y=0.5X+0.5Y$	MI HE X cc	0.04	0.13	0.13	0.13	0.93	-0.01	0.06	0.06	0.07	0.96	0.01	0.06	0.06	0.07	0.96
Logistic, $R_x=0.5X+0.5Y$, $R_y=0.5X+0.5Y$	MI HE	-0.00	0.12	0.12	0.13	0.95	-0.05	0.06	0.08	0.06	0.89	-0.00	0.06	0.06	0.06	0.95
Logistic, $R_x=0.5X+0.5Y$, $R_y=0.5X+0.5Y$	MI HE X fully obs.	0.01	0.12	0.12	0.12	0.95	0.00	0.05	0.05	0.06	0.96	0.00	0.05	0.05	0.06	0.95
Logistic, $R_x=0.5X+0.5Y$, $R_y=0.5X+0.5Y$	Stacking full data	0.06	0.05	0.07	0.07	0.92	-0.06	0.05	0.08	0.06	0.86	0.03	0.05	0.06	0.06	0.96
Logistic, $R_x=0.5X+0.5Y$, $R_y=0.5X+0.5Y$	Stacking X MAR	-0.03	0.05	0.05	0.06	0.98	-0.03	0.05	0.06	0.06	0.94	-0.00	0.05	0.05	0.05	0.97
Logistic, $R_x=0.5X+0.5Y$, $R_y=0.5X+0.5Y$	Stacking X fully obs.	0.01	0.04	0.04	0.06	0.99	0.00	0.05	0.05	0.05	0.96	0.00	0.05	0.05	0.05	0.96
Logistic, $R_x=0.5X+0.5Y$, $R_y=0.5X+0.5Y$	NARFCS Shift SP	0.32	0.10	0.33	0.06	0.03	-0.16	0.06	0.17	0.06	0.26	0.08	0.05	0.10	0.05	0.59
Logistic, $R_x=0.5X+0.5Y$, $R_y=0.5X+0.5Y$	NARFCS Shift full data	-0.00	0.04	0.04	0.06	0.99	0.01	0.05	0.05	0.05	0.94	0.00	0.05	0.05	0.05	0.96
Logistic, $R_x=0.5X+0.5Y$, $R_y=0.5X+0.5Y$	NARFCS Int. full data	0.00	0.03	0.03	0.06	1.00	0.00	0.04	0.04	0.05	0.98	0.00	0.03	0.03	0.05	1.00
Logistic, $R_x=0.5X+0.5Y$, $R_y=0.5X+0.5Y$	NARFCS Int. X fully obs.	-0.00	0.03	0.03	0.05	1.00	0.00	0.03	0.03	0.05	0.99	0.00	0.03	0.03	0.05	0.99
Logistic, $R_x=0.5X+0.5Y$, $R_y=0.5X+0.5Y$	MI RI	0.17	0.08	0.19	0.26	1.00	-0.07	0.05	0.09	0.07	0.88	0.05	0.05	0.07	0.08	0.98
Logistic, $R_x=0.5X+0.5Y$, $R_y=0.5X+0.5Y$	MI RI X fully obs.	0.23	0.08	0.24	0.24	0.98	-0.06	0.05	0.07	0.08	0.96	0.06	0.05	0.08	0.08	0.96

Table S16: (9)Performance of the methods in the simulation study with non-monotone missing. DGM: data-generating mechanism; empse: empirical standard error; rmse: root mean square error; modse: model standard error; cov: empirical coverage of the 95% confidence intervals; see the description of abbreviations in Table S2.

		Intercept					β_x					β_z				
DGM	Method	bias	empse	rmse	modse	cov	bias	empse	rmse	modse	cov	bias	empse	rmse	modse	cov
Pattern-mixture	All cases	-0.00	0.06	0.06	0.05	0.95	-0.00	0.07	0.07	0.05	0.86	-0.00	0.07	0.07	0.05	0.87
Pattern-mixture	CC	0.00	0.06	0.06	0.06	0.95	1.00	0.06	1.00	0.06	0.00	1.00	0.06	1.00	0.06	0.00
Pattern-mixture	MI MAR	-0.00	0.06	0.06	0.06	0.96	1.00	0.06	1.00	0.06	0.00	1.00	0.06	1.00	0.06	0.00
Pattern-mixture	ML HE X cc	0.00	1.26	1.26	Inf	0.66	1.00	0.14	1.01	Inf	0.07	1.00	0.15	1.01	Inf	0.07
Pattern-mixture	ML HE X excl.	-0.08	5.28	5.28	Inf	0.71	0.99	0.26	1.03	Inf	0.10	1.00	0.32	1.05	Inf	0.10
Pattern-mixture	ML HE X fully obs.	0.03	1.19	1.19	Inf	0.67	1.00	0.11	1.00	Inf	0.06	1.00	0.13	1.01	Inf	0.06
Pattern-mixture	UI X cc	-0.25	0.06	0.25	0.07	0.04	1.00	0.06	1.00	0.07	0.00	1.00	0.06	1.00	0.07	0.00
Pattern-mixture	UI X fully obs.	-0.25	0.05	0.25	0.05	0.00	1.00	0.05	1.00	0.05	0.00	1.00	0.05	1.00	0.05	0.00
Pattern-mixture	MI HE X cc	-0.01	0.50	0.50	0.47	0.66	1.00	0.07	1.00	0.08	0.00	1.00	0.07	1.00	0.08	0.00
Pattern-mixture	MI HE	-0.01	0.35	0.35	0.52	0.92	0.99	0.06	1.00	0.07	0.00	1.00	0.06	1.00	0.07	0.00
Pattern-mixture	MI HE X fully obs.	-0.01	0.44	0.44	0.46	0.68	1.00	0.05	1.00	0.05	0.00	1.00	0.05	1.00	0.05	0.00
Pattern-mixture	Stacking full data	-0.00	0.05	0.05	0.05	0.94	1.00	0.06	1.00	0.05	0.00	1.00	0.06	1.00	0.05	0.00
Pattern-mixture	Stacking X MAR	-0.00	0.05	0.05	0.06	0.96	1.00	0.06	1.00	0.06	0.00	1.00	0.06	1.00	0.06	0.00
Pattern-mixture	Stacking X fully obs.	-0.00	0.04	0.04	0.05	0.96	1.00	0.04	1.00	0.05	0.00	1.00	0.05	1.00	0.05	0.00
Pattern-mixture	NARFCS Shift SP	-0.00	0.11	0.11	0.06	0.70	0.99	0.06	1.00	0.06	0.00	1.00	0.06	1.00	0.06	0.00
Pattern-mixture	NARFCS Shift full data	0.00	0.07	0.07	0.06	0.90	0.99	0.06	0.99	0.06	0.00	1.00	0.06	1.00	0.06	0.00
Pattern-mixture	NARFCS Int. full data	-0.00	0.07	0.07	0.09	0.99	-0.35	0.14	0.38	0.12	0.22	-0.00	0.08	0.08	0.09	0.97
Pattern-mixture	NARFCS Int. X fully obs.	-0.00	0.06	0.06	0.06	0.98	-0.00	0.07	0.07	0.06	0.91	-0.00	0.07	0.07	0.06	0.92
Pattern-mixture	MI RI	-0.00	0.09	0.09	0.30	1.00	0.96	0.06	0.96	0.07	0.00	1.00	0.06	1.00	0.06	0.00
Pattern-mixture	MI RI X fully obs.	-0.00	0.07	0.07	0.22	1.00	1.00	0.04	1.00	0.05	0.00	1.00	0.05	1.00	0.05	0.00
Pattern-mixture, $R_x \sim X$	All cases	-0.00	0.06	0.06	0.06	0.96	0.00	0.07	0.07	0.05	0.86	-0.00	0.07	0.07	0.06	0.90
Pattern-mixture, $R_x \sim X$	CC	-0.00	0.09	0.09	0.09	0.95	1.00	0.06	1.00	0.06	0.00	1.00	0.06	1.00	0.06	0.00
Pattern-mixture, $R_x \sim X$	MI MAR	-0.36	0.08	0.36	0.08	0.01	1.09	0.06	1.09	0.06	0.00	1.00	0.06	1.00	0.06	0.00
Pattern-mixture, $R_x \sim X$	ML HE X cc	0.01	3.27	3.27	Inf	0.70	0.96	1.66	1.92	Inf	0.08	1.00	0.29	1.04	Inf	0.09
Pattern-mixture, $R_x \sim X$	ML HE X excl.	-0.25	6.55	6.55	Inf	0.72	0.96	2.11	2.32	Inf	0.08	0.98	0.56	1.13	Inf	0.12
Pattern-mixture, $R_x \sim X$	ML HE X fully obs.	-0.02	1.66	1.66	Inf	0.70	1.00	0.48	1.11	Inf	0.07	1.00	0.10	1.00	Inf	0.06
Pattern-mixture, $R_x \sim X$	UI X cc	-0.25	0.09	0.26	0.10	0.25	1.00	0.06	1.00	0.07	0.00	1.00	0.06	1.00	0.07	0.00
Pattern-mixture, $R_x \sim X$	UI X fully obs.	-0.25	0.05	0.25	0.05	0.00	1.00	0.04	1.00	0.04	0.00	1.00	0.05	1.00	0.05	0.00
Pattern-mixture, $R_x \sim X$	MI HE X cc	-0.00	0.50	0.50	0.48	0.70	1.00	0.07	1.00	0.08	0.00	1.00	0.07	1.00	0.08	0.00
Pattern-mixture, $R_x \sim X$	MI HE	-0.31	0.30	0.43	0.44	0.88	1.08	0.08	1.09	0.11	0.00	1.00	0.06	1.00	0.07	0.00
Pattern-mixture, $R_x \sim X$	MI HE X fully obs.	-0.02	0.45	0.45	0.44	0.68	1.00	0.04	1.00	0.05	0.00	1.00	0.05	1.00	0.05	0.00
Pattern-mixture, $R_x \sim X$	Stacking full data	-0.26	0.08	0.28	0.09	0.10	1.01	0.06	1.01	0.07	0.00	1.00	0.06	1.00	0.06	0.00
Pattern-mixture, $R_x \sim X$	Stacking X MAR	-0.52	0.08	0.53	0.09	0.00	1.09	0.06	1.09	0.06	0.00	1.00	0.06	1.00	0.06	0.00
Pattern-mixture, $R_x \sim X$	Stacking X fully obs.	-0.15	0.05	0.16	0.05	0.15	1.00	0.04	1.00	0.04	0.00	1.00	0.05	1.00	0.05	0.00
Pattern-mixture, $R_x \sim X$	NARFCS Shift SP	-0.53	0.15	0.55	0.08	0.00	1.08	0.06	1.08	0.06	0.00	1.00	0.06	1.00	0.06	0.00
Pattern-mixture, $R_x \sim X$	NARFCS Shift full data	0.00	0.08	0.08	0.07	0.89	0.77	0.05	0.77	0.05	0.00	1.00	0.06	1.00	0.06	0.00
Pattern-mixture, $R_x \sim X$	NARFCS Int. full data	0.11	0.09	0.15	0.12	0.90	-0.27	0.12	0.30	0.11	0.34	0.00	0.08	0.08	0.10	0.97
Pattern-mixture, $R_x \sim X$	NARFCS Int. X fully obs.	-0.00	0.06	0.06	0.07	0.98	0.00	0.07	0.07	0.06	0.91	-0.00	0.07	0.07	0.07	0.93
Pattern-mixture, $R_x \sim X$	MI RI	-0.44	0.11	0.46	0.33	0.89	1.04	0.06	1.04	0.09	0.00	1.00	0.06	1.00	0.07	0.00
Pattern-mixture, $R_x \sim X$	MI RI X fully obs.	0.00	0.07	0.07	0.22	1.00	1.00	0.04	1.00	0.04	0.00	1.00	0.05	1.00	0.05	0.00

Table S17: (10)Performance of the methods in the simulation study with non-monotone missing. DGM: data-generating mechanism; empse: empirical standard error; rmse: root mean square error; modse: model standard error; cov: empirical coverage of the 95% confidence intervals; see the description of abbreviations in Table S2.

S3.3 Simulation study 2 additional results for Uuncertainty intervals assuming $\rho \in [-0.99, 0.99]$

Tables S18 and S19 provide the empirical coverage and mean width of the uncertainty intervals in simulation study 2. The uncertainty intervals were calculated assuming the correlation between the errors in the outcome and selection models ranges from -0.99 to 0.99. For all data-generating mechanisms, except the ones generated from a pattern-mixture model, the empirical coverage of uncertainty intervals is close to 1, as expected, because the assumed range for ρ is close to the maximal possible range of [-1, 1]. High empirical coverage comes at the cost of wide confidence intervals.

		Empirical coverage			Mean width of UI		
Data-generating mechanism	Method	Intercept	β_x	β_z	Intercept	β_x	β_z
MAR	All cases	0.95	0.95	0.95	0.1	0.1	0.1
MAR	UI X cc	1	0.99	1	1.2	0.3	0.6
MAR	UI X fully observed	1	1	1	1.6	0.3	0.9
Heckman, $R_x \sim X$	All cases	0.94	0.94	0.95	0.1	0.1	0.1
Heckman, $R_x \sim X$	UI X cc	1	1	1	1.1	0.3	0.6
Heckman, $R_x \sim X$	UI X fully observed	1	1	1	1.6	0.3	0.9
Heckman, $R_x \sim Y$	All cases	0.95	0.94	0.95	0.1	0.1	0.1
Heckman, $R_x \sim Y$	UI X cc	1	0.99	1	1.1	0.3	0.6
Heckman, $R_x \sim Y$	UI X fully observed	1	1	1	1.6	0.3	0.9
Heckman, $R_x \sim X$ and Y	All cases	0.95	0.95	0.94	0.1	0.1	0.1
Heckman, $R_x \sim X$ and Y	UI X cc	1	0.99	1	1.1	0.3	0.6
Heckman, $R_x \sim X$ and Y	UI X fully observed	1	0.99	1	1.6	0.3	0.9
Heckman, $R_y \sim X$	All cases	0.95	0.95	0.94	0.1	0.1	0.1
Heckman, $R_y \sim X$	UI X cc	1	1	1	1.1	0.4	0.6
Heckman, $R_y \sim X$	UI X fully observed	1	1	1	1.6	0.4	0.9
Heckman, $R_y \sim Y$	All cases	0.95	0.94	0.94	0.1	0.1	0.1
Heckman, $R_y \sim Y$	UI X cc	1	0.99	1	1.1	0.3	0.6
Heckman, $R_y \sim Y$	UI X fully observed	1	1	1	1.6	0.3	0.9
Heckman, $R_y \sim X$ and Y	All cases	0.95	0.96	0.94	0.1	0.1	0.1
Heckman, $R_y \sim X$ and Y	UI X cc	1	1	1	1.1	0.4	0.6
Heckman, $R_y \sim X$ and Y	UI X fully observed	1	1	1	1.6	0.4	0.9
Heckman, $R_x \sim X$, $R_y \sim X$	All cases	0.95	0.95	0.94	0.1	0.1	0.1
Heckman, $R_x \sim X$, $R_y \sim X$	UI X cc	1	1	1	1.1	0.3	0.6
Heckman, $R_x \sim X$, $R_y \sim X$	UI X fully observed	1	1	1	1.6	0.4	0.9
Heckman, $R_x \sim X$, $R_y \sim Y$	All cases	0.95	0.95	0.95	0.1	0.1	0.1
Heckman, $R_x \sim X$, $R_y \sim Y$	UI X cc	1	0.99	1	1.1	0.3	0.6
Heckman, $R_x \sim X$, $R_y \sim Y$	UI X fully observed	1	1	1	1.6	0.3	0.9
Heckman, $R_x \sim X$, $R_y \sim X$ and Y	All cases	0.94	0.94	0.95	0.1	0.1	0.1
Heckman, $R_x \sim X$, $R_y \sim X$ and Y	UI X cc	1	1	1	1.1	0.3	0.6
Heckman, $R_x \sim X$, $R_y \sim X$ and Y	UI X fully observed	1	1	1	1.6	0.4	0.9
Heckman, $R_x \sim Y$, $R_y \sim X$	All cases	0.96	0.95	0.95	0.1	0.1	0.1
Heckman, $R_x \sim Y$, $R_y \sim X$	UI X cc	1	1	1	1.1	0.4	0.6
Heckman, $R_x \sim Y$, $R_y \sim X$	UI X fully observed	1	1	1	1.6	0.4	0.9
Heckman, $R_x \sim Y$, $R_y \sim Y$	All cases	0.96	0.95	0.95	0.1	0.1	0.1
Heckman, $R_x \sim Y$, $R_y \sim Y$	UI X cc	1	0.99	1	1.1	0.3	0.6
Heckman, $R_x \sim Y$, $R_y \sim Y$	UI X fully observed	1	1	1	1.6	0.3	0.9
Heckman, $R_x \sim Y$, $R_y \sim X$ and Y	All cases	0.95	0.95	0.95	0.1	0.1	0.1
Heckman, $R_x \sim Y$, $R_y \sim X$ and Y	UI X cc	1	1	1	1.1	0.4	0.6
Heckman, $R_x \sim Y$, $R_y \sim X$ and Y	UI X fully observed	1	1	1	1.6	0.4	0.9
Heckman, $R_x \sim X$ and Y, $R_y \sim X$	All cases	0.94	0.94	0.95	0.1	0.1	0.1
Heckman, $R_x \sim X$ and Y, $R_y \sim X$	UI X cc	1	1	1	1.1	0.3	0.6
Heckman, $R_x \sim X$ and Y, $R_y \sim X$	UI X fully observed	1	1	1	1.6	0.4	0.9
Heckman, $R_x \sim X$ and Y, $R_y \sim Y$	All cases	0.94	0.94	0.95	0.1	0.1	0.1
Heckman, $R_x \sim X$ and Y, $R_y \sim Y$	UI X cc	1	0.99	1	1.1	0.3	0.6
Heckman, $R_x \sim X$ and Y, $R_y \sim Y$	UI X fully observed	1	1	1	1.6	0.3	0.9
Heckman, $R_x \sim X$ and Y, $R_y \sim X$ and Y	All cases	0.96	0.95	0.96	0.1	0.1	0.1
Heckman, $R_x \sim X$ and Y, $R_y \sim X$ and Y	UI X cc	1	1	1	1.1	0.3	0.6
Heckman, $R_x \sim X$ and Y, $R_y \sim X$ and Y	UI X fully observed	1	1	1	1.6	0.4	0.9

Table S18: Empirical coverage and mean width of uncertainty intervals assuming $\rho \in [-0.99, 0.99]$ in simulation study 2. UI: uncertainty interval; UI X fully observed: uncertainty intervals assuming the predictor X is fully observed.

		Empirical coverage			Mean width of UI		
Data-generating mechanism	Method	Intercept	β_x	β_z	Intercept	β_x	β_z
Logistic, R_x=0.5X	All cases	0.95	0.94	0.96	0.1	0.1	0.1
Logistic, R_x=0.5X	UI X cc	1	1	1	1.9	0.4	0.8
Logistic, R_x=0.5X	UI X fully observed	1	1	1	2.1	0.3	1.0
Logistic, R_x=0.5Y	All cases	0.96	0.94	0.96	0.1	0.1	0.1
Logistic, R_x=0.5Y	UI X cc	1	1	1	1.9	0.4	0.9
Logistic, R_x=0.5Y	UI X fully observed	1	1	1	2.1	0.3	1.0
Logistic, R_x= 0.5X+0.5Y	All cases	0.94	0.95	0.95	0.1	0.1	0.1
Logistic, R_x= 0.5X+0.5Y	UI X cc	1	1	1	1.9	0.4	0.9
Logistic, R_x= 0.5X+0.5Y	UI X fully observed	1	1	1	2.1	0.3	1.0
Logistic, R_y=0.5X	All cases	0.95	0.95	0.95	0.1	0.1	0.1
Logistic, R_y=0.5X	UI X cc	1	1	1	1.9	0.6	0.8
Logistic, R_y=0.5X	UI X fully observed	1	1	1	2.1	0.6	0.9
Logistic, R_y=0.5Y	All cases	0.95	0.95	0.93	0.1	0.1	0.1
Logistic, R_y=0.5Y	UI X cc	1	1	1	1.9	0.5	0.7
Logistic, R_y=0.5Y	UI X fully observed	1	1	1	2.1	0.5	0.8
Logistic, R_y= 0.5X+0.5Y	All cases	0.95	0.95	0.95	0.1	0.1	0.1
Logistic, R_y= 0.5X+0.5Y	UI X cc	1	1	1	1.9	0.8	0.7
Logistic, R_y= 0.5X+0.5Y	UI X fully observed	1	1	1	2.1	0.8	0.8
Logistic, R_x=0.5X, R_y=0.5X	All cases	0.93	0.95	0.95	0.1	0.1	0.1
Logistic, R_x=0.5X, R_y=0.5X	UI X cc	1	1	1	1.8	0.5	0.8
Logistic, R_x=0.5X, R_y=0.5X	UI X fully observed	1	1	1	2.1	0.6	0.9
Logistic, R_x=0.5X, R_y=0.5Y	All cases	0.94	0.95	0.93	0.1	0.1	0.1
Logistic, R_x=0.5X, R_y=0.5Y	UI X cc	1	1	1	1.9	0.4	0.7
Logistic, R_x=0.5X, R_y=0.5Y	UI X fully observed	1	1	1	2.1	0.5	0.8
Logistic, R_x=0.5X, R_y= 0.5X+0.5Y	All cases	0.96	0.95	0.94	0.1	0.1	0.1
Logistic, R_x=0.5X, R_y= 0.5X+0.5Y	UI X cc	1	1	1	1.9	0.7	0.7
Logistic, R_x=0.5X, R_y= 0.5X+0.5Y	UI X fully observed	1	1	1	2.1	0.8	0.8
Logistic, R_x=0.5Y, R_y=0.5X	All cases	0.95	0.95	0.94	0.1	0.1	0.1
Logistic, R_x=0.5Y, R_y=0.5X	UI X cc	1	1	1	1.8	0.6	0.8
Logistic, R_x=0.5Y, R_y=0.5X	UI X fully observed	1	1	1	2.1	0.6	0.9
Logistic, R_x=0.5Y, R_y=0.5Y	All cases	0.94	0.95	0.95	0.1	0.1	0.1
Logistic, R_x=0.5Y, R_y=0.5Y	UI X cc	1	1	1	1.8	0.4	0.7
Logistic, R_x=0.5Y, R_y=0.5Y	UI X fully observed	1	1	1	2.1	0.5	0.8
Logistic, R_x=0.5Y, R_y= 0.5X+0.5Y	All cases	0.95	0.95	0.96	0.1	0.1	0.1
Logistic, R_x=0.5Y, R_y= 0.5X+0.5Y	UI X cc	1	1	1	1.8	0.7	0.7
Logistic, R_x=0.5Y, R_y= 0.5X+0.5Y	UI X fully observed	1	1	1	2.1	0.8	0.8
Logistic, R_x= 0.5X+0.5Y, R_y=0.5X	All cases	0.94	0.95	0.95	0.1	0.1	0.1
Logistic, R_x= 0.5X+0.5Y, R_y=0.5X	UI X cc	1	1	1	1.8	0.5	0.8
Logistic, R_x= 0.5X+0.5Y, R_y=0.5X	UI X fully observed	1	1	1	2.1	0.6	0.9
Logistic, R_x= 0.5X+0.5Y, R_y=0.5Y	All cases	0.95	0.95	0.96	0.1	0.1	0.1
Logistic, R_x= 0.5X+0.5Y, R_y=0.5Y	UI X cc	1	0.99	1	1.8	0.4	0.7
Logistic, R_x= 0.5X+0.5Y, R_y=0.5Y	UI X fully observed	1	1	1	2.1	0.5	0.8
Logistic, R_x= 0.5X+0.5Y, R_y= 0.5X+0.5Y	All cases	0.96	0.93	0.95	0.1	0.1	0.1
Logistic, R_x= 0.5X+0.5Y, R_y= 0.5X+0.5Y	UI X cc	1	1	1	1.8	0.7	0.7
Logistic, R_x= 0.5X+0.5Y, R_y= 0.5X+0.5Y	UI X fully observed	1	1	1	2.1	0.8	0.8
Pattern-mixture	All cases	0.95	0.86	0.87	0.2	0.2	0.2
Pattern-mixture	UI X cc	1	0.00	0.00	3.1	0.6	0.6
Pattern-mixture	UI X fully observed	1	0.00	0.00	2.9	0.4	0.4
Pattern-mixture, $R_x \sim X$	All cases	0.96	0.86	0.90	0.3	0.2	0.2
Pattern-mixture, $R_x \sim X$	UI X cc	1	0.00	0.00	3.3	0.6	0.6
Pattern-mixture, $R_x \sim X$	UI X fully observed	1	0.00	0.00	3.0	0.4	0.4

Table S19: (Continued)Empirical coverage and mean width of uncertainty intervals assuming $\rho \in [-0.99, 0.99]$ in simulation study 2. UI: uncertainty interval; UI X fully observed: uncertainty intervals assuming the predictor X is fully observed.

S4 Empirical study

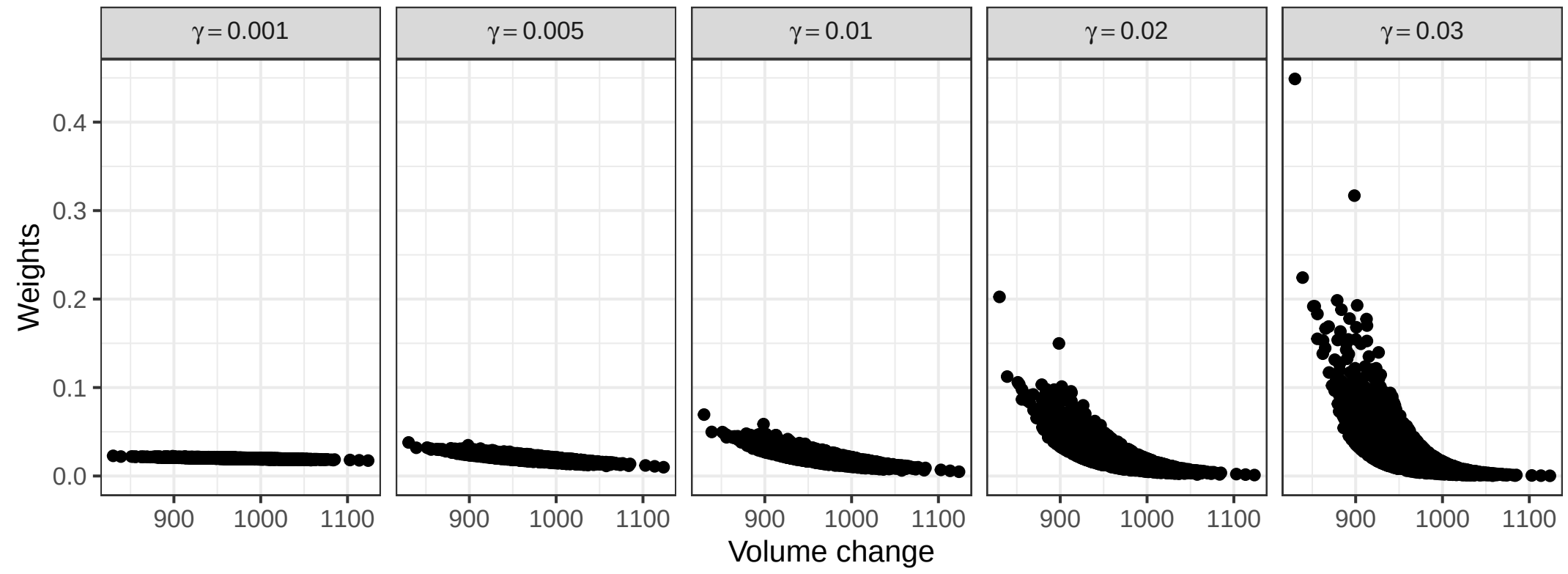

Figure S4: Weights for imputed Volume change versus imputed Volume change for imputation stacking assuming memory change is missing at random in the empirical study.

S4.1 Details of imputation stacking assuming MNAR volume and memory changes

According to Beesley and Taylor,(5) if assuming missing not at random memory and brain changes, the following parameters and models should be specified to define the weights (we use Z to denote fully observed predictors Age and Hypertension):

1. $P(R_{\text{volume change}} = 1|\text{volume change}, \text{memory change}, Z, R_{\text{memory change}} = 0)$.

2. the coefficient for volume change in $P(R_{\text{volume change}} = 1|\text{volume change}, \text{ memory change}, Z)$, assuming a logistic model for this probability, otherwise the whole model should be specified.

3. $P(R_{\text{memory change}} = 1|\text{volume change}, \text{memory change}, Z, R_{\text{volume change}} = 0)$.

4. the coefficient for memory change in $P(R_{\text{memory change}} = 1|\text{volume change}, \text{memory change}, Z)$ assuming a logistic model for this probability, otherwise the whole model should be specified.

5. $P(R_{\text{volume change}} = 0, R_{\text{memory change}} = 0|\text{volume change}, \text{ memory change}, Z)$.

6. $P(R_{\text{volume change}} = 1, R_{\text{memory change}} = 1|\text{volume change}, \text{ memory change}, Z)$.

To limit the number of parameters that require specification, we assume that response indicators in 1, 3, 5, 6 depend only on fully observed Age and Hypertension, that is, the coefficients for memory and volume changes are 0. This implies that, for the cases missing

both memory and volume change, all imputed values are given equal weight. As before, we use 0.001, 0.005, 0.01, 0.02, and 0.03 as the values for the coefficient for volume change in the logistic regression for the response indicator for volume change in 2. Similarly, given that the observed memory changes vary from -10 to 7 approximately, we choose 0.001, 0.5, 1, 2, and 5 as the values of the coefficient for memory change in the logistic regression for the response indicator for memory change in 4. Supplementary Table S20 presents point estimates and 95% confidence intervals assuming a normal distribution approximation as point estimate ± 1.96× `StackImpute` jackknife modification of the standard error estimate. For all considered combinations of the parameters, the 95% confidence intervals for the coefficient for memory change do not include 0.

γ_{memory}	γ_{volume}	Intercept	Memory change	Age	Hypertension
0.001	0.001	1099.40(1054.26,1144.54)	2.53(0.67,4.40)	-1.81(-2.52,-1.10)	-6.64(-17.37,4.10)
0.001	0.005	1101.08(1054.96,1147.19)	2.53(0.65,4.41)	-1.84(-2.57,-1.11)	-6.68(-17.53,4.17)
0.001	0.01	1103.36(1054.35,1152.36)	2.53(0.63,4.43)	-1.89(-2.67,-1.11)	-6.74(-17.88,4.40)
0.001	0.02	1108.53(1043.98,1173.07)	2.54(0.50,4.57)	-2.00(-3.05,-0.94)	-6.84(-19.42,5.74)
0.001	0.03	1114.04(1018.07,1210.01)	2.55(0.21,4.89)	-2.11(-3.71,-0.51)	-6.94(-22.78,8.91)
0.5	0.001	1099.82(1054.63,1145.01)	2.62(0.77,4.47)	-1.81(-2.52,-1.10)	-6.64(-17.38,4.11)
0.5	0.005	1101.49(1055.36,1147.62)	2.61(0.76,4.47)	-1.85(-2.57,-1.12)	-6.69(-17.55,4.17)
0.5	0.01	1103.77(1054.79,1152.75)	2.61(0.73,4.49)	-1.89(-2.67,-1.12)	-6.76(-17.91,4.40)
0.5	0.02	1108.93(1044.47,1173.39)	2.60(0.59,4.61)	-2.00(-3.05,-0.95)	-6.88(-19.48,5.73)
0.5	0.03	1114.42(1018.54,1210.30)	2.60(0.29,4.91)	-2.11(-3.71,-0.51)	-7.00(-22.89,8.89)
1	0.001	1100.23(1055.10,1145.36)	2.68(0.86,4.50)	-1.82(-2.53,-1.11)	-6.68(-17.44,4.08)
1	0.005	1101.91(1055.85,1147.97)	2.67(0.85,4.49)	-1.85(-2.58,-1.13)	-6.73(-17.61,4.15)
1	0.01	1104.19(1055.30,1153.09)	2.66(0.81,4.50)	-1.90(-2.68,-1.12)	-6.80(-17.99,4.39)
1	0.02	1109.37(1044.99,1173.74)	2.64(0.68,4.60)	-2.01(-3.06,-0.95)	-6.92(-19.59,5.74)
1	0.03	1114.87(1019.00,1210.73)	2.63(0.39,4.86)	-2.12(-3.72,-0.52)	-7.05(-23.01,8.91)
2	0.001	1100.34(1055.23,1145.44)	2.70(0.90,4.51)	-1.82(-2.53,-1.11)	-6.67(-17.44,4.11)
2	0.005	1102.02(1055.99,1148.05)	2.69(0.88,4.50)	-1.85(-2.58,-1.13)	-6.72(-17.62,4.18)
2	0.01	1104.32(1055.46,1153.18)	2.68(0.85,4.50)	-1.90(-2.68,-1.12)	-6.78(-17.99,4.43)
2	0.02	1109.52(1045.17,1173.86)	2.65(0.72,4.59)	-2.01(-3.06,-0.96)	-6.90(-19.59,5.80)
2	0.03	1115.04(1019.16,1210.93)	2.63(0.44,4.82)	-2.12(-3.73,-0.52)	-7.01(-23.02,8.99)
5	0.001	1100.27(1055.12,1145.43)	2.71(0.91,4.50)	-1.82(-2.53,-1.11)	-6.67(-17.45,4.11)
5	0.005	1101.96(1055.89,1148.04)	2.69(0.89,4.49)	-1.85(-2.58,-1.12)	-6.72(-17.62,4.19)
5	0.01	1104.27(1055.38,1153.16)	2.68(0.85,4.50)	-1.90(-2.68,-1.12)	-6.78(-18.00,4.43)
5	0.02	1109.47(1045.11,1173.83)	2.65(0.72,4.58)	-2.01(-3.06,-0.95)	-6.90(-19.60,5.80)
5	0.03	1115.01(1019.10,1210.91)	2.63(0.44,4.82)	-2.12(-3.73,-0.52)	-7.01(-23.03,9.01)

Table S20: Point estimates and 95% confidence intervals for regression parameters in the empirical study provided by imputation stacking assuming memory and volume changes might be missing not at random. γ_{memory}: the coefficient for memory change in $P(R_{\text{memory change}} = 1|\text{volume change}, \text{memory change}, Z)$ assuming a logistic model for this probability; γ_{volume}: the coefficient for volume change in $P(R_{\text{volume change}} = 1|\text{volume change}, \text{ memory change}, Z)$, assuming a logistic model for this probability.

References

[1] van Buuren S and Groothuis-Oudshoorn K. mice: Multivariate Imputation by Chained Equations in R. *Journal of Statistical Software* 2011; 45(3): 1–67. DOI:10.18637/jss.v045.i03. URL https://www.jstatsoft.org/index.php/jss/article/view/v045i03.

[2] Toomet O and Henningsen A. Sample selection models in R: Package sampleSelection. *Journal of Statistical Software* 2008; 27(7). URL https://www.jstatsoft.org/v27/i07/.

[3] Genbäck M, Stanghellini E and de Luna X. Uncertainty intervals for regression parameters with non-ignorable missingness in the outcome. *Statistical Papers* 2015; 56: 829–847.

[4] Galimard JE, Chevret S, Curis E et al. Heckman imputation models for binary or continuous MNAR outcomes and MAR predictors. *BMC Medical Research Methodology* 2018; 18(1): 1–13.

[5] Beesley LJ and Taylor JM. Accounting for not-at-random missingness through imputation stacking. *Statistics in Medicine* 2021; 40(27): 6118–6132.

[6] Tompsett DM, Leacy F, Moreno-Betancur M et al. On the use of the not-at-random fully conditional specification (NARFCS) procedure in practice. *Statistics in Medicine* 2018; 37(15): 2338–2353.

[7] Jolani S and van Buuren S. Random indicator imputation for missing not at random data. *arXiv preprint* 2024; arXiv: 2404.14534.

[8] van Buuren S and Groothuis-Oudshoorn CG. Multivariate imputation by chained equations, 2000.

[9] van Buuren S, Brand JP, Groothuis-Oudshoorn CG et al. Fully conditional specification in multivariate imputation. *Journal of Statistical Computation and Simulation* 2006; 76(12): 1049–1064.

[10] van Buuren S. Multiple imputation of discrete and continuous data by fully conditional specification. *Statistical Methods in Medical Research* 2007; 16(3): 219–242.

[11] Moreno-Betancur M, Leacy F, Tompsett D et al. mice: The NARFCS procedure for sensitivity analyses (Available at: https://github.com/moreno-betancur/NARFCS), 2019.
